# AI-enabled discovery and biochemical optimization of minibinders targeting cancer cell-surface proteins

**DOI:** 10.1038/s41467-026-76760-5

**Published:** 2026-08-20

**Authors:** Bianca Broske, Benjamin A. McEnroe, Sophie C. Frechen, Tim N. Kempchen, Caroline I. Fandrey, Elisabeth Tan, Dominic Ferber, Michelle C. R. Yong, Marie Kleinert, Julia M. Messmer, Peter Konopka, Alexander Hoch, Katja Blumenstock, Jan M. P. Tödtmann, Johannes Oldenburg, Heiko Rühl, Alexander Semaan, Marieta I. Toma, Kristina Markova, Sebastian Kobold, Tim Rollenske, Matthias Geyer, Stephan Menzel, Tobias Bald, Jonathan L. Schmid-Burgk, Gregor Hagelueken, Michael Hölzel

**Affiliations:** 1 Institute for Experimental Oncology, University of Bonn, University Hospital Bonn, Bonn, Germany; 2 Institute of Structural Biology, University of Bonn, University Hospital Bonn, Bonn, Germany; 3 Institute for Clinical Chemistry and Clinical Pharmacology, University of Bonn, University Hospital Bonn, Bonn, Germany; 4 Core Facility Nanobodies, University of Bonn, University Hospital Bonn, Bonn, Germany; 5 Institute for Experimental Hematology and Transfusion Medicine, University of Bonn, University Hospital Bonn, Bonn, Germany; 6 Institute for Clinical Hemostaseology and Transfusion Medicine, Saarland University, Homburg, Germany; 7 Department of Surgery, University of Bonn, University Hospital Bonn, Bonn, Germany; 8 Department of General and Visceral Surgery, Charité – University Medicine Berlin, Campus Benjamin Franklin, Berlin, Germany; 9 Institute for Pathology, University of Bonn, University Hospital Bonn, Bonn, Germany; 10 Institute for Molecular Medicine and Experimental Immunology, University of Bonn, University Hospital Bonn, Bonn, Germany; 11 Institute of Clinical Pharmacology, LMU University Hospital, LMU Munich, Munich, Germany; 12 German Cancer Consortium (DKTK), Partner Site Munich, A Partnership Between the DKFZ Heidelberg and the LMU University Hospital, LMU Munich, Munich, Germany; 13 Einheit für Klinische Pharmakologie (EKLiP), Helmholtz Zentrum München – German Research Center for Environmental Health Neuherberg, Munich, Germany; 14 German Center for Lung Research (DZL), Partner site Munich, Munich, Germany; 15 Institute for Innate Immunity, University of Bonn, University Hospital Bonn, Bonn, Germany

**Keywords:** Drug discovery, Cancer therapy, Protein design, Molecular biophysics, High-throughput screening

## Abstract

Experimental validation and functional optimization remain bottlenecks in AI-based protein design. We present a scalable workflow for developing AI-designed minibinders against cancer-associated surface proteins. Screening thousands of designs using mammalian cell-surface display identifies several high-affinity PD-L1 minibinders but far fewer for CD276 (B7-H3) and VTCN1 (B7-H4), highlighting substantial target dependence. Interface predicted template modeling (ipTM) scores generated by Chai-1 with ESM embeddings correlate with binding success and capture deleterious effects of interface mutations. Fluorophore-labeled AI-minibinders enable flow-cytometric staining comparable to conventional antibodies. However, when incorporated into chimeric antigen receptors (CAR), some show poor cell-surface trafficking and limited functionality. Redesign through a genetic algorithm-based diversification strategy that preserves the binding interface while changing non-binding surfaces experimentally reveals an isoelectric point (pI) window that improves CAR expression and enhances target-selective tumor cell killing. Our findings identify biochemical optimization beyond the binding interface as a critical requirement for translating AI-minibinders into functional applications.

## Abstract (web summary)

In this work the authors present a scalable mammalian cell-display workflow to identify AI-designed minibinders against cancer surface targets. AI-guided optimization beyond the binding interface improves their expression as chimeric antigen receptors and target-selective killing.

## Introduction

For many decades, structural biology has provided detailed insights into the mechanisms of molecular recognition that govern the interplay between the multitude of large and small molecules within living cells^1^. The corresponding interaction interfaces have been shaped by evolution to achieve context-specific affinities and specificities. Ever since the first structures of such complexes were determined experimentally, scientists have been studying ways to engineer such interfaces artificially, for example by designing or selecting small-molecule inhibitors or antibodies against a target protein.

Artificial intelligence (AI) has dramatically changed structural biology in recent years as the introduction of AI-based algorithms such as AlphaFold (AF2, AF3)^2,3^, RoseTTAFold^4,5^, or ESMfold^6^ has made precise protein structure prediction possible. Additionally, algorithms such as RFdiffusion^7^, AlphaProteo^8^, BindCraft^9^, and Chroma^10^ are able to design either completely artificial proteins or proteins that adhere to user-defined design principles, such as following a particular fold or binding to a target molecule of interest. Demonstrating the accessibility of such workflows is key to unlocking the broader potential of AI-based protein design, particularly for therapeutic applications such as cancer immunotherapy.

In this work, we describe our developed accessible pipeline for designing small artificial protein binders (AI-minibinders) against three cancer surface targets of the B7-H family and a scalable experimental workflow for their validation. To this end, we adapt the open-source algorithms RFdiffusion and AF2 to our specific use case, by implementing custom filter scripts that employ available software tools such as the CCP4^11^ suite or Biopython^12^. Using this pipeline, we design thousands of candidate minibinders and identify functional hits through a combination of mammalian cell-surface display screening, fluorescence-activated cell sorting (FACS), and next-generation sequencing. The resulting AI-minibinders are biophysically characterized and converted into useful research tools, including open-source flow cytometry detection reagents and chimeric antigen receptors (CAR) for CAR-T cells. However, translation into the CAR format reveals an additional engineering challenge, as some de novo designed minibinders show poor cell-surface trafficking. Therefore, we investigate how biochemical properties beyond the binding interface influence CAR surface expression and function. Our analysis identifies the isoelectric point (pI) as a key determinant of CAR surface trafficking and leads to a scalable optimization strategy to improve the functional performance of AI-minibinders in CAR formats.

## Results

### Pipeline for AI-minibinder design and functional screening

**Fig. 1:**
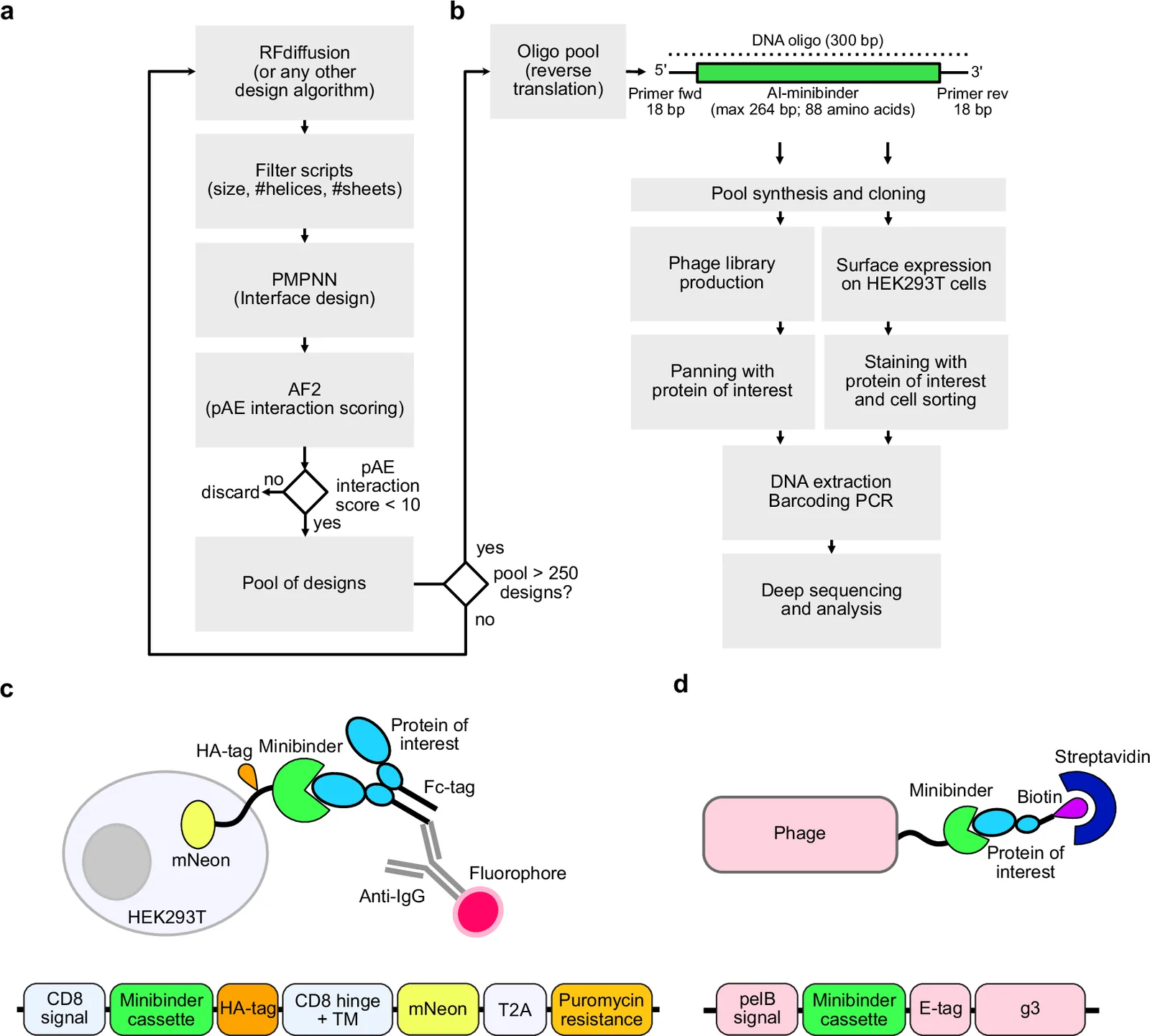
Computational design and functional screening strategy for AI-designed protein minibinders. **a** Outline of the RFdiffusion-based design pipeline with structural filtering steps. **b** Outline of the functional screening pipelines. **c** Scheme illustrating the principle of mammalian cell-surface display and the construct used for AI-minibinder presentation. **d** Scheme illustrating the principle of phage display and the construct used for AI-minibinder presentation. Schemes were generated using Affinity Publisher 2.

Following the methodology developed by Watson et al.^7^, we generated poly‑glycine scaffolds using RFdiffusion (length between 70 and 88 amino acids), then applied ProteinMPNN (PMPNN)^13,14^ to design amino‑acid side chains (two variants per scaffold), and finally evaluated each sequence with the AlphaFold2 (AF2) initial‑guess algorithm^14^ (Fig. 1a). To triage designs, we employed the predicted aligned error (pAE) interaction score from AF2^7^, a per-residue-pair confidence metric that estimates the expected positional error for each pair of interface residues. In practice, the mean inter‑chain pAE has been shown to be a reliable proxy for interface accuracy, correlating with experimental success^7^.

Depending on the target, RFdiffusion produced designs with good pAE interaction scores (pAE <10)^7^ with strongly varying efficiency. Within our design constraints (70–88 amino acids), we noted that the algorithm often produced alpha-helical hairpins with two rather long alpha helices. Small-scale expression tests indicated poor expression or stability of such designs, probably because these designs do not have a defined hydrophobic core to provide stability. Nevertheless, we noticed that some of the designs had a much more compact fold, similar to affibodies^15^, with three or four short alpha helices that were predicted to form stable alpha-helical bundles with an extensive hydrophopic core based on recent literature^16–18^. To prevent restricting RFdiffusion by forcing it to adhere to a defined fold, we decided to implement a Biopython^12^ script that uses the dssp software^19,20^ to detect secondary structure elements and thereby simply discarded any designs that had less than three alpha-helices. Only the designs that passed this selection step were fed into the subsequent stages of our pipeline, thereby saving computation time in amino acid sequence design with ProteinMPNN and pAE interaction scoring with AF2. We were confident that the resulting designs should have a better chance to work in real-world applications. Thus, rather than improving design quality per se, our structural filtering step increases workflow efficiency by reducing the GPU hours needed to obtain experimentally promising designs, which is particularly relevant for scaling design campaigns and for researchers with limited HPC access.

Interestingly, we also observed that PMPNN frequently created sequences that exhibited surface-exposed patches of alanines (poly-Ala patches) that lie remotely from the binding interface. It should be noted that our pipeline is not restricted to RFdiffusion but also works with designs that are created with any other algorithm such as Bindcraft^9^ or Chroma^10^.

To identify functional AI-minibinders at scale, we implemented two pooled experimental screening approaches for targeted enrichment (Fig. 1b). For mammalian cell-surface display, we encoded each AI-minibinder in a chimeric antigen receptor-like construct (Fig. 1c), allowing its presentation on the mammalian cell surface. Cells bearing functional AI-minibinders are detected by staining with the target protein and isolated via fluorescence-activated cell sorting (FACS). In the second approach, phages displaying functional AI-minibinders are enriched by incubating with the target protein immobilized on streptavidin beads, followed by bead-based purification (Fig. 1d). In both workflows, enriched populations are subjected to DNA extraction, and the AI-minibinder coding sequences served as identifiers for next-generation sequencing (NGS) analysis of PCR-amplified fragments. We restricted our designed AI-minibinders to 88 amino acids (≙264 base pairs; bp) since 300 bp DNA oligo pools, including both the AI-minibinder and primer sequences (each 18 bp), proved to be the most cost-effective way to generate libraries with thousands of potential candidates for testing (Fig. 1b).

### Design and screening of AI-minibinders against B7-H family proteins

**Fig. 2:**
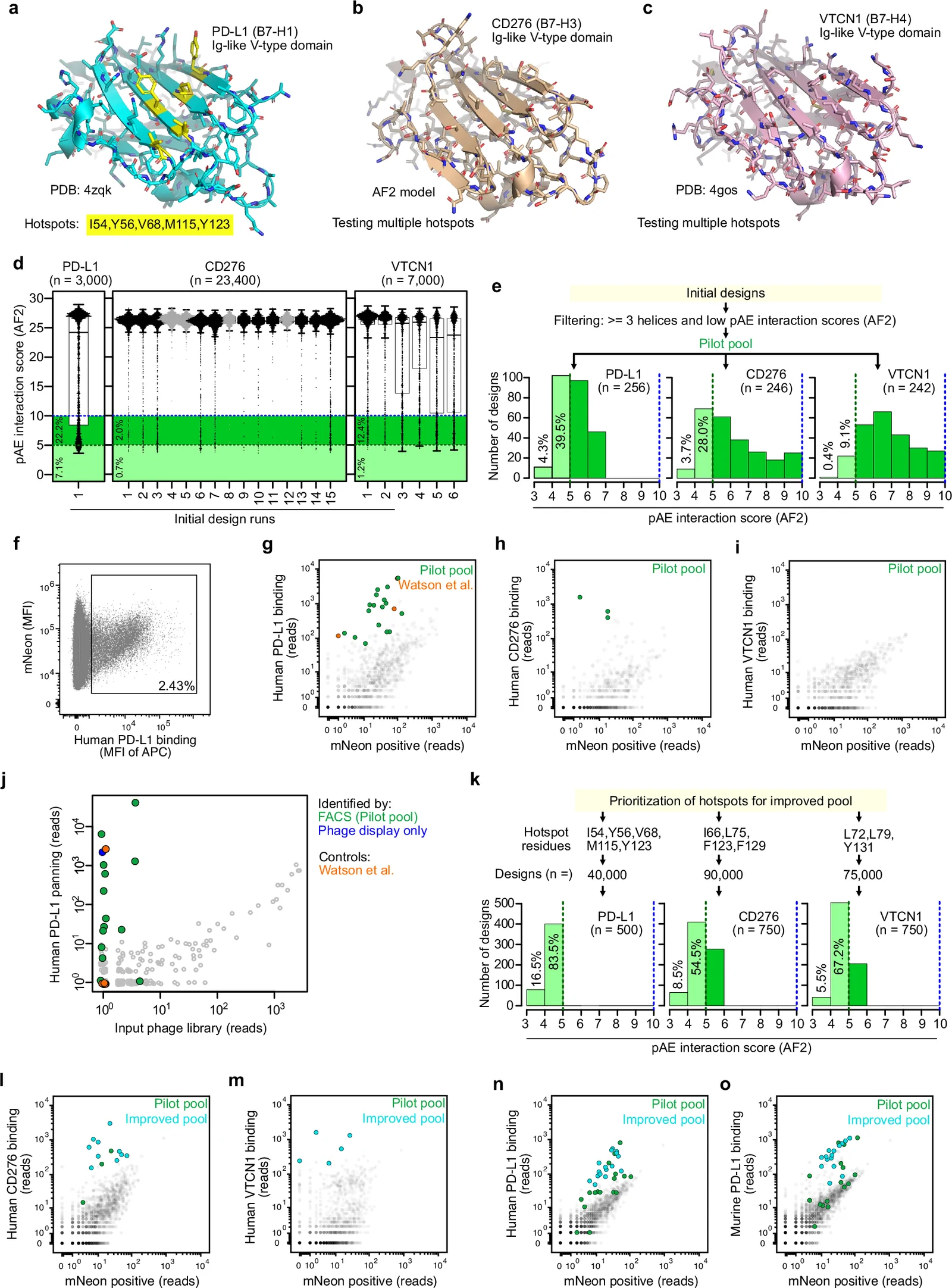
De novo design and experimental screening of AI-minibinders targeting B7-H family proteins. **a**–**c** Cartoons of the PD-L1 (B7-H1) crystal structure (PDB: 4zqk), CD276 (B7-H3) AlphaFold2 (AF2) model, and VTCN1 (B7-H4) crystal structure (PDB: 4gos). **d** Boxplots with data points summarizing AF2 pAE interaction scores of individual minibinders generated in indicated design runs. The total number of minibinder designs per target is indicated as well as the percentage of designs with low (good) scores. Boxplots show the median (center line), interquartile range (box; 25th–75th percentiles), and whiskers extending to the most extreme values within 1.5× IQR below the first quartile or above the third quartile. **e** Histogram depicting the distribution of pAE interaction scores of the best designs used for the pilot pool. **f** Representative scatter plot (from *n* = 3 sorts) illustrating a subpopulation of HEK293T cells expressing minibinders from the pilot pool binding to human PD-L1 Fc-fusion protein (produced in HEK293 cells). **g**–**i** Scatter plots showing enriched AI-minibinders (marked in indicated colors) for PD-L1, CD276, and VTCN1 identified by NGS analysis of the respective subpopulation binding the human target protein (Fc-fusion proteins produced in HEK293 cells). **j** NGS analysis of panning by phage display showing enriched PD-L1 AI-minibinders. Minibinders also identified by mammalian cell-surface display are marked in green, binders only identified by phage display in blue, and positive controls in orange. **k** Histograms showing the distribution of pAE interaction scores for designs selected into the improved pool (*n* = 500 or 750) from the total initial design sets (*n* = 40,000, 90,000, or 75,000). **l**–**o** Scatter plots showing enriched AI-minibinders from the improved pool for CD276, VTCN1, and PD-L1 identified by NGS analysis of the respective subpopulation binding the human or murine target protein (Fc-fusion proteins produced in HEK293 cells). Source data are provided as a Source Data file.

B7-H family proteins, including PD-L1 (B7-H1), CD276 (B7-H3), and VTCN1 (B7-H4), are well-established surface targets for cancer therapy^21–24^. We set out to design AI-minibinders against the Ig-like V-type domains leveraging our computational and experimental screening pipeline, using both crystal structures (PD-L1, VTCN1) and AF2 models (CD276) of these surface receptors as input (Fig. 2a–c). Human PD‑L1 was included as a benchmarking target, since Watson et al. previously designed AI-minibinders against PD‑L1 using RFdiffusion^7^, enabling us to select a patch of hydrophobic amino acids at the binding interface of their validated PD-L1 minibinders as hotspot for our design process. Furthermore, this hotspot was located opposite to an evolutionary glycosylation site (N-M-T, position 35 in PD-L1 Q9NZQ7-1) avoiding potential interferences by glycosylation. We confirmed that RFdiffusion generated a large number of candidate PD-L1 minibinders with promising pAE interaction scores below 10 (about 22%), and even below 5 in roughly 7% of the designs (Fig. 2d). In contrast, the design of candidate binders against human CD276 and VTCN1 was less efficient, despite testing multiple hotspots (Supplementary Data 1). These hotspots were defined as distinct combinations of hydrophobic amino acids that may form favorable interaction patches and thereby increase the likelihood of successful minibinder design, in line with the recommendations of the RFdiffusion developers^7^ (https://github.com/RosettaCommons/RFdiffusion). Furthermore, hotspots were intentionally positioned opposite glycosylation sites to minimize potential interference. After filtering for designs with at least three alpha-helices, we selected about 250 AI-minibinder designs for each target (Fig. 2e). We also added AI-minibinder designs from Watson et al.^7^ against PD-L1, TrkA (NTRK1), and insulin receptor (INSR) as controls to obtain a pilot pool with a total of 1000 designs (Supplementary Data 2, 3). Human HEK293T cells were transduced with the minibinder library, stained with human PD‑L1, CD276, or VTCN1 Fc‑fusion proteins produced in human HEK293 cells, and sorted by FACS (*n* = 3 independent experiments) (Supplementary Fig. 1a). We detected a 2.4% positive PD‑L1-binding population, compared with below 1% for both CD276 and VTCN1 (Fig. 2f, Supplementary Fig. 1b, c). NGS analyses confirmed these differences, identifying multiple enriched PD-L1 binders, fewer for CD276, and none for VTCN1 (Fig. 2g–i). The mNeon positive-sorted cell population was used as reference to normalize for differences in AI-minibinder representation within the pool. Enriched AI‑minibinder candidates deviate above the diagonal toward the top of the plot and separate from the main cloud. Notably, our screen also identified positive control PD-L1 minibinders previously designed and validated by Watson et al. Phage display generated largely congruent enrichments for PD‑L1 minibinders (Fig. 2j). No clear outliers were identified for CD276 and VTCN1 in the phage‑display screen, consistent with the mammalian cell‑surface display results (Supplementary Fig. 1d, e).

Given that the CD276 and VTCN1 libraries exhibited higher (i.e., worse) pAE interaction scores than the PD‑L1 library, we hypothesized that expanding the number of designs to include more candidates with better scores would improve the success rates for CD276 and VTCN1 (Fig. 2k). For the second round of computational design, we selected those hotspots for CD276 and VTCN1 identified in the first screening pool (Supplementary Fig. 1f, g; Supplementary Data 4–6) that yielded the highest number of designs with pAE scores below 5. Screening of the improved pool (*n* = 2000 designs) resulted in additional hits for CD276 and few candidate VTCN1 minibinders (Fig. 2l, m). For PD‑L1, we likewise identified additional minibinders, and comparative staining with murine PD‑L1 Fc-fusion protein produced in human HEK293 cells indicated that our library contains cross‑reactive, as well as human‑ and mouse-specific PD-L1 minibinders (Fig. 2n, o).

### Characterization of de novo designed PD-L1 minibinders

**Fig. 3:**
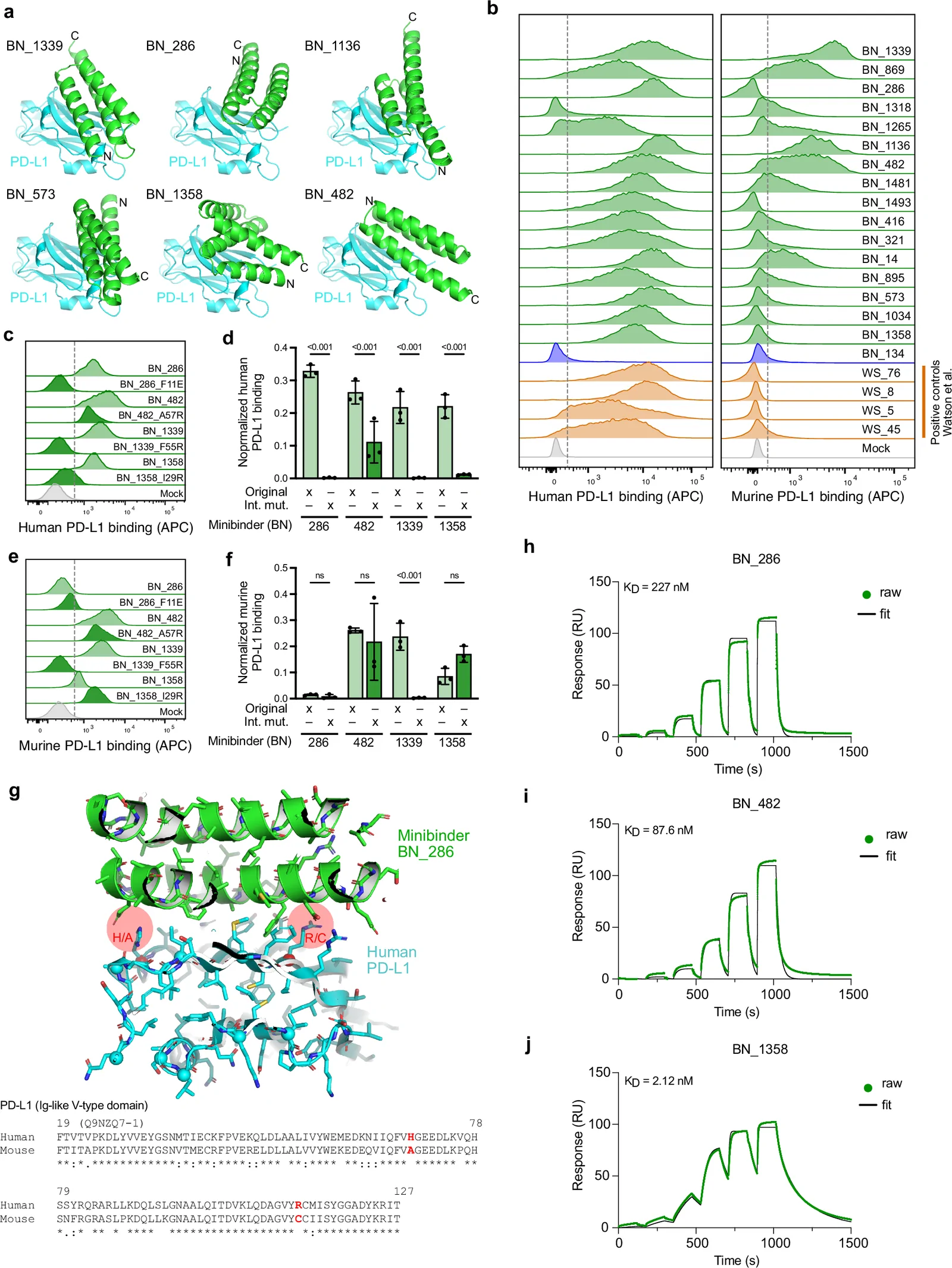
Characterization of de novo designed PD-L1 AI-minibinders reveals both species selectivity and cross-reactivity. **a** Cartoon representation of the predicted complexes between PD-L1 AI-minibinders and the Ig-like V-type domain of human PD-L1. N, C: N- or C-terminus. **b** Flow cytometric analyses evaluating the binding of individual PD-L1 minibinders by HEK293T cell-surface display to human and murine PD-L1 Fc-fusion proteins (produced in HEK293 cells). Minibinders identified by mammalian cell-surface display are marked in green; only identified by phage display in blue, and positive controls in orange. **c** Experiment and analysis as described in (**b**) but for PD-L1 AI-minibinder interface mutants binding to human and murine PD-L1. Representative histograms (**c**, **e**) and quantification (**d**, **f**) of the experiments from biological triplicates (*n* = 3 experiments). Geometric mean fluorescence intensity (MFI) of binding is normalized to HA-tag signal (cell-surface expression of AI-minibinder). **g** Cartoon representation with side chains of the predicted complex between PD-L1 minibinder BN_286 (green) and human PD-L1 (cyan). Residues highlighted in red might explain selective binding to human but not murine PD-L1. **h**–**j** SPR (surface plasmon resonance) sensorgrams showing raw binding data (green) and fitted curves (black) used to determine binding affinities (K_D_) of PD-L1 minibinders (produced in *E.coli*) BN_286, BN_482, and BN_1358 binding to human PD-L1 (produced in HEK293 cells). Five-fold concentration series were used ranging from 1.6 nM to 5000 nM (BN_286), 0.32 nM to 1000 nM (BN_482), 0.064 nM to 200 nM (BN_1358). ns, non-significant; two-way ANOVA with Šídák’s multiple comparisons test. Error bars indicate mean ±SD. Source data are provided as a Source Data file.

Figure 3a shows the predicted structures of a subset of identified PD-L1 minibinders from the pilot pool in complex with the Ig-like V-type domain of human PD-L1. For validation, individual PD-L1 minibinders were expressed on HEK293T cells, including the positive control minibinders from Watson et al. (WS_76, WS_8, WS_5, WS_45)^7^, and assessed by flow cytometry for binding to human and murine PD-L1 (Fig. 3b; Supplementary Fig. 2a). Efficient cell-surface expression was confirmed by detection of the HA-tag incorporated in the extracellular part of the construct (Fig. 1c, Supplementary Fig. 2b). Consistent with the screening results, 15 of 17 PD-L1 minibinders confirmed binding to human PD-L1. Interestingly, several were cross-reactive to the murine ortholog of PD-L1.

To confirm that the designed interfaces formed as predicted, we introduced mutations at the binding interface of our PD-L1 minibinders and tested whether these alterations would disrupt the interaction with their target. Specifically, non-conservative substitutions were introduced by replacing predicted key hydrophobic residues with bulky polar amino acids, expected to induce steric clashes and disrupt hydrophobic interactions critical for binding. The resulting AI-minibinder interface mutants were expressed on the surface of HEK293T cells, stained with human PD-L1 Fc-fusion protein and analyzed by flow cytometry (Fig. 3c, d, Supplementary Fig. 2c). Interface mutations abolished human PD‑L1 binding in three of the four AI‑minibinders (BN_286_F11E, BN_1339_F55R, BN_1358_I29R), while the fourth showed only a partial reduction in binding (BN_482_A57R). Additionally, we stained with murine PD-L1 Fc-fusion protein and obtained similar results for BN_1339_F55R and BN_482_A57R. Interestingly, we observed improved binding to murine PD-L1 for BN_1358_I29R (Fig. 3e, f). Furthermore, we confirmed selective binding of BN_286 to human PD-L1, which is supported by inspection of the predicted structure of the complex, revealing that two prominent interactions are not possible with murine PD-L1 (Fig. 3g).

Equivalent to PD-L1, we expressed individual CD276 and VTCN1 minibinders, as well as their interface mutants, on HEK293T cells and stained with the respective Fc-tagged target proteins. While binding patterns were weaker than those of PD-L1 minibinders, the interface mutants confirmed minibinder-target interactions (Supplementary Fig. 3).

In order to thoroughly study the biophysical properties of our AI-minibinders, we expressed the proteins in *E. coli*. We considered that the aforementioned surface alanine patches might promote aggregation of the proteins during expression and therefore mutated selected alanine sidechains to polar residues. Supplementary Fig. 4a shows that the AI-minibinder proteins, including the original variants with alanine patches, expressed very well in *E. coli*. Analytical gel filtration experiments during purification yielded peaks at the expected elution volumes for monomeric proteins with no visible aggregates (Supplementary Fig. 4b). We conducted nanoscale differential scanning fluorimetry (nanoDSF) experiments of Trp or Tyr containing AI-minibinders BN_482 and BN_1358 to determine their thermal stability at elevated temperatures and found that the designs remained stable and even refolded when the temperature was reduced again (Supplementary Fig. 4c). To confirm this observation and to further assess the thermal stability of the designed proteins, we heated lysates of AI-minibinder expressing *E. coli* at 94 °C for 20 min and found substantial amounts of AI-minibinders in the cleared supernatant of all constructs (Supplementary Fig. 4d). Of note for functional assays, *E. coli* lacks the N-glycosylation machinery, which may lead to binding differences, however, none of the purified PD-L1 minibinders contained a canonical N-X-S/T N-glycosylation motif (Supplementary Data 2). Analyzing the entire pool of minibinders revealed a marked underrepresentation of asparagines compared to the normal human proteome (1.48% vs. 4.3–4.4%; binomial test, *p* ≪ 0.001) in line with recent analyses benchmarking various protein design algorithms^25^, thus minibinders designed by RFdiffusion exhibit an underrepresentation of potential N-X-S/T N-glycosylation motifs.

Next, we used surface plasmon resonance (SPR) spectroscopy to determine binding characteristics. We immobilized a fragment of human PD-L1 containing the extracellular domain purified from HEK293 cells to the sensor surface and injected concentration series of the mini binders BN_286, BN_482, and BN_1358 purified from *E. coli*. Concordant with flow cytometry data, we detected strong interactions between PD-L1 and the AI-minibinders with dissociation constants in the range of 2–230 nM (Fig. 3h–j). Here, BN_1358 was identified as the strongest binder (K_D_ = 2 nM) with a relatively slow off-rate, compared to binders BN_482 (K_D_ = 87 nM) and BN_286 (K_D_ = 227 nM). Notably, replacing the poly-Ala patches outside the binding interface with hydrophilic residues resulted in only modest changes in both the association and dissociation rates compared to the original AI‑binders (Supplementary Fig. 5).

### Computational scores versus experimental success rates

**Fig. 4:**
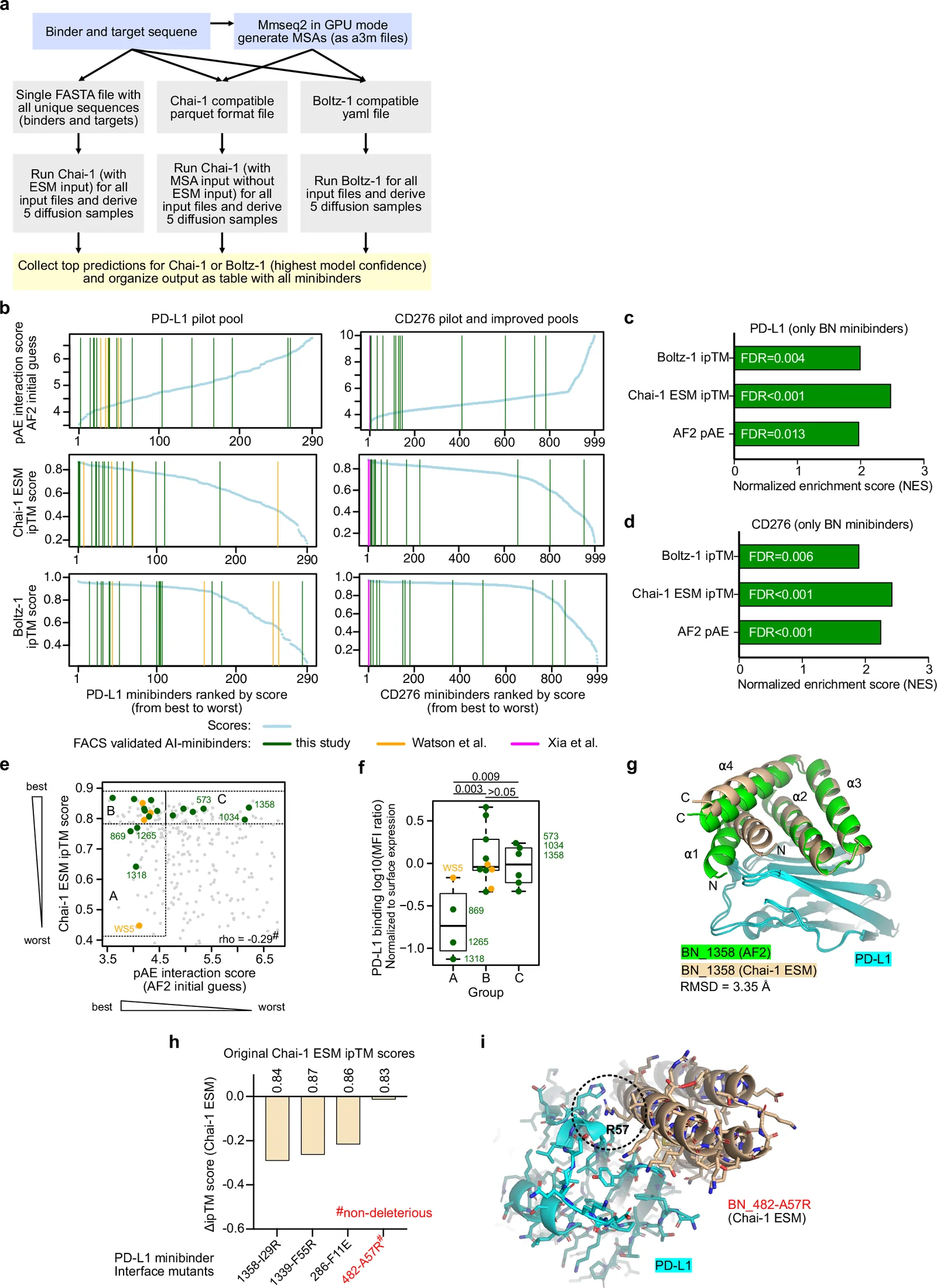
Association between interaction scores generated by different open-source structure prediction models and experimental success of AI-minibinders. **a** Outline of computational strategy for re-scoring of AI-minibinder pools and generation of ipTM scores by Chai-1 and Boltz-1. **b** Enrichment plots visualizing correlation between depicted interaction scores (predicted by AlphaFold2 (AF2), Chai-1, or Boltz-1) and experimental success. AI-minibinders from indicated pools ranked from left to right by interaction scores (best to worst). Colored vertical lines indicate functional AI-minibinders from our study (green), or from other studies (orange, magenta). **c**, **d** Normalized enrichment scores (NES) reflecting the association between interaction scores and experimental success in the PD-L1 AI-minibinder pilot pool and the CD276 AI-minibinder pool (combined pilot and improved pool). Permutation (*n* = 1000)-based significance testing. FDR (false discovery rate) values corrected for multiple testing are indicated. **e** Scatter plot showing the correlation between pAE (AF2) and ipTM (Chai-1 ESM) interaction scores for the PD-L1 pilot pool. Functional PD-L1 AI-minibinders from our study highlighted in green, and some labeled with identifiers. Positive controls from other study in orange. Rho: Spearman’s rank correlation coefficient. ^#^*p* = 6.38e−07, two-sided. Groups A, B, and C indicate concordant or discordant scores. **f** Log10-transformed geometric mean fluorescence intensity (MFI) ratio of PD-L1 binding normalized to AI-minibinder surface expression (HA-tag), compared by groups described in **e**. The groups consist of *n* = 4 (A), *n* = 10 (B), and *n* = 6 (C) different minibinders. Boxplots show the median (center line), interquartile range (box; 25th–75th percentiles), and whiskers extending to the most extreme values within 1.5× IQR below the first quartile or above the third quartile. Two-sided pairwise *t*-test, corrected for multiple testing (FDR). **g** Overlay of BN_1358 bound to PD-L1, shown in cartoon representation, with structures predicted by AlphaFold2 (AF2) (green) and Chai-1 with ESM embeddings (beige). Root mean square deviation (RMSD) determined for binder structure predictions only. Å Ångström. α-helices are numbered from N-terminus onwards. N, C: N- or C-termini. **h** Change in Chai-1 ESM ipTM score caused by interface mutations in PD-L1 AI-minibinders. **i** Cartoon representation of the BN_A482-A57R interface mutant in complex with Ig-like V-type domain of human PD-L1, as predicted by Chai-1 with ESM embeddings. The outward position of R57 is highlighted with a dashed circle. Source data are provided as a Source Data file.

Using RFdiffusion, the efficiency and success rate of designing functional AI‑minibinders varied widely among our three targets, although they all belong to the same family of B7-H proteins (about 30% amino acid identity in their Ig-like V-type domains). Thus, we asked how well different in silico interaction scores predict experimental binding and if they could help to streamline the pipeline. Besides the AF2 pAE interaction score generated during the initial design with RFdiffusion, we re-scored the designs with two recently released open-source models, Chai-1^26^ and Boltz-1^27^. Figure 4a outlines our computational re-scoring strategy that uses protein sequences (FASTA format) as input data, which are extracted from PDB files. Multiple sequence alignments (MSA) for Boltz-1 were locally generated with Mmseq2 in GPU mode^28^. We used Chai-1 with ESM protein language model embeddings as input^29^. Both models generate ipTM scores (interface predicted template modeling) that range between 0 (worst) and 1 (best), where ipTM scores higher than 0.7 were considered promising. All designs from the PD-L1, CD276, and VTCN1 pools, together with PD-L1^7^ and CD276^30^ minibinder controls, were ranked based on the respective scores from best to worst (Fig. 4b). Highlighting that the experimentally validated AI-minibinders indeed showed a clear correlation between better scores and experimental success, though this analysis was limited for VTCN1 due to the low numbers of functional AI-minibinders (Supplementary Fig. 6a). Of note, the positive control CD276 minibinders mb and HM9^30^ were ranked highest by all three models. In a side-by-side comparison, the Chai-1 ESM ipTM score showed the highest normalized enrichment scores (NES), and thus best performance, albeit the difference was modest (Fig. 4c, d).

Although the AF2 pAE interaction and the Chai-1 ESM ipTM scores correlated well, we also observed disagreement when analyzing the scores of the PD-L1 minibinders. As highlighted in Fig. 4e, our experimentally validated PD‑L1 minibinders segregated into two discordant groups (A and C) and one concordant group (B). Integration of normalized flow cytometry data revealed that group C binders exhibited higher PD-L1 binding than group A, suggesting that the Chai‑1 ESM ipTM score outperforms the pAE interaction score as a predictor of experimental binding strength, at least in our limited dataset (Fig. 4f). In this context, BN_1358 was a notable example, given its high affinity (K_D_ = 2 nM) determined by SPR spectroscopy (Fig. 3j), the relatively high (poor) AF2 pAE interaction score of 6.2, and the high (good) Chai-1 ESM ipTM score of 0.84. Overlay of the complexes predicted by AF2 initial guess within RFdiffusion and Chai-1 ESM showed a marked shift of the N-terminal alpha helix, with Chai-1 ESM predicting a more compact conformation of BN_1358 (Fig. 4g).

### Prediction of interface mutation effects by Chai-1

Intriguingly, re-scoring of PD‑L1 minibinder interface mutants using Chai‑1 ESM revealed that three of the four mutants exhibited a clear decrease in their ipTM scores (ΔipTM) relative to their matched original PD‑L1 minibinders (Fig. 4h; Supplementary Data 7). Notably, the fourth mutant (BN_482_A57R) retained an ipTM score comparable to the original minibinder and, in line with this prediction, had shown significant residual binding activity by flow cytometry (Fig. 3c, d). According to the Chai‑1 prediction, R57 undergoes an outward twist instead of imposing the intended steric hindrance at the binding interface, giving a possible explanation for our experimental observation (Fig. 4i). When rescoring the CD276 minibinder interface mutants, we also observed reduction in ipTM scores for 11 of the 12 mutants, with changes (ΔipTM) ranging from 0.01 (BN_486-1043_M23E) to 0.57 (BN_616-606_L10R), possibly reflecting differences in the binding strength of the matched original minibinders and accuracy of the initial prediction (Supplementary Fig. 6b).

We then compared ΔipTM scores for deleterious interface mutants predicted by AlphaFold3 (AF3) and Chai-1 ESM (Supplementary Fig. 6c). Interestingly, the two sets of ΔipTM scores did not significantly correlate, highlighting differences in the prediction methodologies (Supplementary Fig. 6d). Nonetheless, both AF3 and Chai-1 ESM ΔipTM scores captured the deleterious impact of interface mutations, though to variable degrees, with Chai-1 ESM ΔipTM scores being slightly more pronounced (Supplementary Fig. 6e).

### Quattrobinders as open-source flow cytometry reagents

**Fig. 5:**
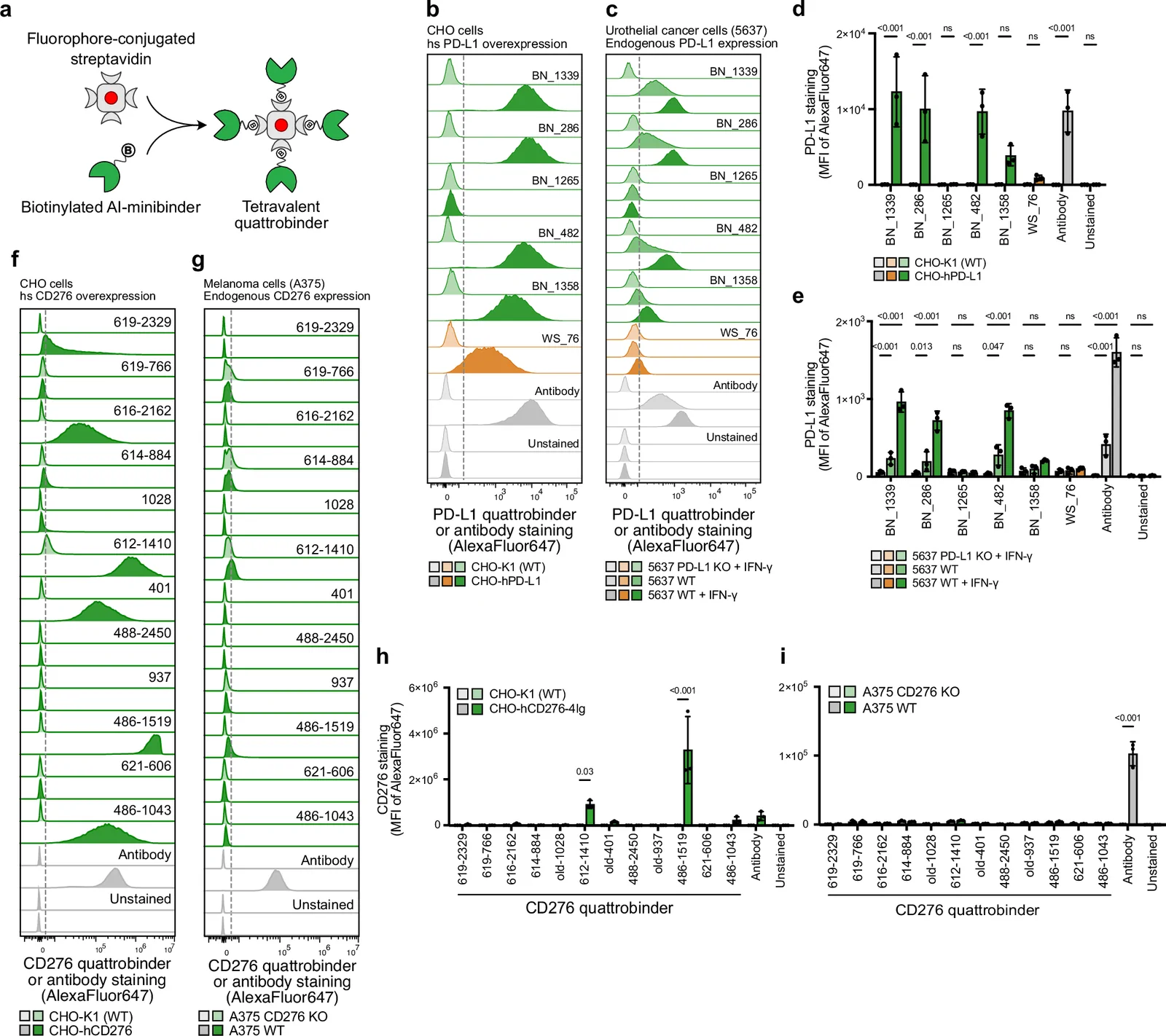
PD-L1 AI-minibinders assembled as fluorescent quattrobinders specifically detect endogenous PD-L1 expression on cancer cells. **a** Cartoon representing the process of quattrobinder assembly of biotinylated minibinders produced in *E.coli*. Generated using Affinity Publisher 2. **b**–**i** Flow cytometric analyses showing detection of human PD-L1 and CD276 by AF647-conjugated quattrobinders compared to AF647-conjugated anti-PD-L1 or anti-CD276 antibodies as representative histograms (**b**, **c**, **f**, **g**) and quantified results of the geometric mean fluorescence intensity (MFI) (**d**, **e**, **h**, **i**) from biological triplicates (*n* = 3 experiments). Target detection of overexpressed human PD-L1 (**b**, **d**) or human CD276 (**f**, **h**) is shown on Chinese hamster ovarian (CHO) cells. Detection of endogenous PD-L1 expression (**c**, **e**) is shown on human 5637 urothelial cancer cells, either untreated (WT) or stimulated with IFN-γ (WT + IFN-γ). 5637 CRISPR-Cas9-engineered PD-L1 knockout (KO) cells are shown as a reference for specificity of the signals. Detection of endogenous CD276 expression (**g**, **i**) is shown on human A375 melanoma cells endogenously expressing CD276 (WT) or CRISPR-Cas9-engineered CD276 knockout (KO) cells. ns non-significant; two-way ANOVA with Šídák’s multiple comparisons test. Error bars indicate mean ±SD. Source data are provided as a Source Data file.

The ability to design specific, high-affinity AI-minibinders has many practical implications and may help address long-standing problems, such as the widespread demand for open‑source protein detection tools^31–33^. These are particularly valuable for applications like flow cytometry experiments. Hence, we decided to assess the usability of AI-minibinders as staining reagents. To enable site‑specific biotinylation, we introduced a single cysteine residue into the minibinder surface, opposite of its binding interface. The modified minibinders were then expressed in *E. coli* (Supplementary Fig. 4a), biotinylated, and complexed with fluorophore‑conjugated streptavidin to form a tetravalent staining reagent, which we have termed ‘quattrobinder’ (Fig. 5a). Expression of the majority of our cysteine‑engineered minibinders in *E. coli* proved highly efficient, producing up to 120 mg per 2 L of culture. We selected four of our best PD-L1 minibinders for quattrobinder formation, as well as one of our poorest PD-L1 minibinders (BN_1265) to span a broad performance range. We then stained Chinese hamster ovarian (CHO) cells overexpressing human PD-L1 with either AF647-labeled PD-L1 quattrobinders or a conventional AF647-labeled PD-L1 antibody (Supplementary Fig. 2d). As shown in Fig. 5b, our best PD‑L1 quattrobinders produced signal intensities on par with the commercial antibody and exhibited negligible background by flow cytometry.

We then evaluated the ability of the PD-L1 quattrobinders to detect endogenous PD‑L1 in the human urothelial carcinoma cell line 5637, which displays low basal PD‑L1 expression that is upregulated upon IFN-γ stimulation. To confirm target specificity and rule out residual background, we generated a CRISPR‑Cas9-engineered PD‑L1 knockout cell line (5637 PD‑L1 KO) and directly compared PD-L1 quattrobinder staining in wild‑type (WT), IFN-γ stimulated, and knockout cells (Fig. 5c). The PD‑L1 quattrobinders exhibited no detectable background staining on 5637 PD‑L1 KO cells. 5637 WT cells exhibited robust labeling that was markedly enhanced after IFN‑γ stimulation. Overall, intensity and staining patterns of the PD-L1 quattrobinders closely mirrored those of the anti‑PD‑L1 antibody in a robust and reproducible manner (Fig. 5d, e). Similar results were seen on the thyroid cancer cell line FTC-133, however, BN_1339 showed background staining on the PD-L1 knockout cell line that was not observed for 5637 cells (Supplementary Fig. 7a). Unexpectedly, the BN_1358 quattrobinder produced weaker signals than other high-performing variants, despite its strong binding affinity (K_D_ = 2 nM) (Fig. 3j).

We also tested a subset of CD276 and VTCN1 minibinders as quattrobinders. Several CD276 quattrobinders clearly labeled CD276-overexpressing CHO cells (Fig. 5f, h). However, only BN_486-1519 produced a marginal, yet not significant, signal in 5637 WT cells, and no staining was observed in human A375 melanoma WT cells or human U87MG glioblastoma WT cells (Fig. 5g, i; Supplementary Fig. 7b, c). When benchmarked against a commercial anti‑CD276 antibody, the signals generated by our CD276 quattrobinders were negligible in endogenous settings. Furthermore, all VTCN1 quattrobinders exhibited poor performance with substantial background staining even on CHO WT cells (Supplementary Fig. 7d, e). Collectively, our data demonstrate that AI-quattrobinders can, in principle, be used equivalently to conventional antibodies in flow cytometry and enable specific detection of endogenous protein expression levels, although their quality and specificity require rigorous validation and the efficiency remains highly target dependent.

### Functional validation of AI-minibinders in CAR-T cells

Given the poor sensitivity of some quattrobinders against endogenous target proteins, we set out to leverage the chimeric antigen receptor (CAR)-T cell approach for evaluation of our AI-minibinders. CAR-T cells recognize surface antigens on cancer cells, therefore providing a complimentary approach for evaluating AI-minibinder functionality as recently shown by others^30^. The human PD-L1 reactive AI-minibinders BN_1358, BN_482, and BN_286 were cloned into a second-generation CAR expression construct with an additional HA-tag for standardized detection and quantification of CAR surface expression, and the RQR8 peptide separated by a P2A cleavage site (Supplementary Fig. 8a). RQR8 is displayed on the cell membrane and recognized by a CD34-specific antibody (clone QBEND10)^34^, enabling straightforward monitoring and enrichment of T cells transduced with the CAR construct (Supplementary Fig. 8b). CD28 was selected as the costimulatory domain because it is clinically validated (e.g., in Yescarta and Tecartus) and promotes stronger short-term cytotoxicity and cytokine secretion compared to 4-1BB^35,36^. We reasoned that this profile is well suited for in vitro assessment of antigen-specific activation and provides a lower activation threshold, accommodating the broad affinity range of AI-designed minibinders. Upon co-culture, murine T cells expressing the PD-L1 binder CAR selectively recognized CHO target cells overexpressing human PD-L1 compared to controls determined by intracellular cytokine staining (Supplementary Fig. 8c, d). Importantly, PD-L1 binder CAR-T cells also selectively recognized endogenously expressed PD-L1 on human cancer cells compared to matched CRISPR-Cas9-engineered PD-L1 knockout (KO) cells (Supplementary Fig. 8e, f). Together, these findings demonstrate that RFdiffusion‑designed AI‑minibinder CAR‑T cells can effectively target endogenously expressed cancer surface proteins.

### Variable cell surface trafficking of CD276 minibinder CARs

**Fig. 6:**
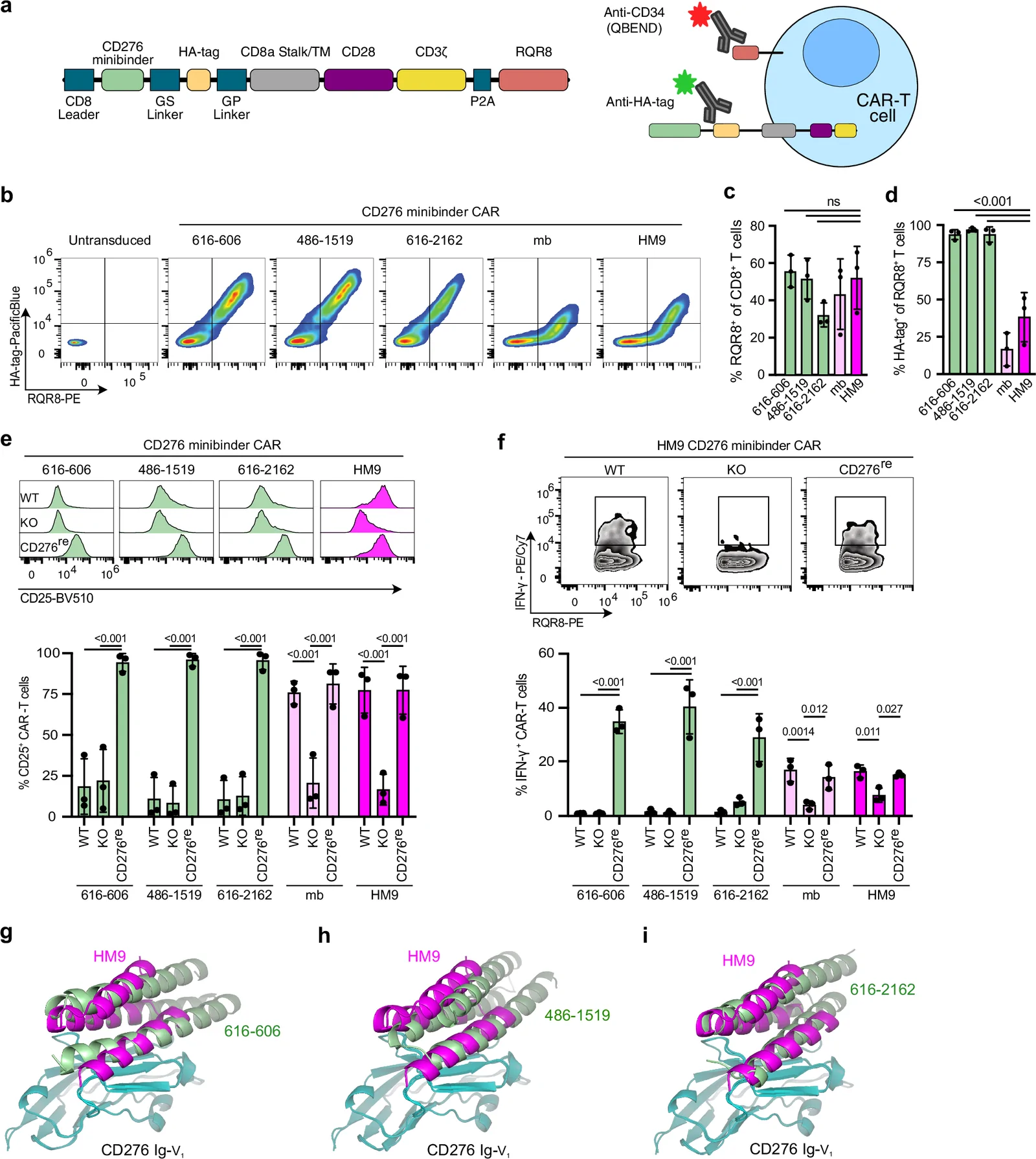
CD276 AI-minibinders deployed in CAR-T cells reveal critical roles of binding affinity and surface expression for endogenous target recognition. **a** Outline of deployed CAR construct and flow cytometric strategy to assess transduction efficiency (RQR8 via anti-CD34 antibody) and CAR cell surface expression (HA-tag). Cartoon generated using Affinity Publisher 2. **b** Representative flow cytometric plots showing surface expression of RQR8 and CARs (HA-tag) of indicated CD276 AI-minibinders on human T cells. **c** Corresponding quantification of transduction efficiency from experiments performed in biological triplicates (*n* = 3 PBMC donors). **d** Corresponding quantification of AI-minibinder CAR surface expression on transduced RQR8^+^ human T cells. Experiments performed in biological triplicates (*n* = 3 PBMC donors). **e** Flow cytometric analyses of CD25 surface expression on indicated CD276 AI-minibinder CAR-T cells co-cultured with A375 melanoma cells endogenously expressing CD276 (WT), CRISPR-Cas9-engineered CD276 knockout (KO) cells, or reconstituted CD276 KO cells (CD276^re^), respectively, with corresponding quantification below (*n* = 3 PBMC donors). **f** Co-culture as described in e. Representative flow cytometric plots showing IFN-γ induction in HM9 CD276 CAR-T cells as an example and corresponding quantification of indicated CD276 AI-minibinder CAR-T cells below (*n* = 3 PBMC donors). **g**–**i** Cartoon representation of AF3 structure models showing overlay of HM9 and CD276 AI-minibinders BN_616-606, BN_486-1519, and BN_616-2162 bound to the Ig-like V-type domain of human CD276. ns, non-significant; two-way ANOVA with Šídák’s multiple comparisons test. Error bars indicate mean ±SD. Source data are provided as a Source Data file.

Encouraged by these results, we applied the CAR‑T cell platform to assess our AI‑minibinders against CD276 (B7‑H3), an attractive target for CAR-T cell therapy^22,30,37^, using our three most promising CD276 minibinder candidates (BN_616-606, BN_486-1519, and BN_616-2162) (Fig. 6a). A recent study also reported AI-designed CD276 binders (mb and HM9)^30^, though a different design algorithm was used^30,38^, followed by multiple rounds of experimental affinity maturation. Of note, a picomolar binding affinity was reported for HM9^30^, so we included the mb and HM9 CD276 minibinders as positive controls in our assays. Transduction of human T cells was successful for all five CAR constructs achieving comparable efficiencies. However, we noted markedly reduced surface expression of mb and HM9 containing CARs compared to our in-house CD276 minibinder CARs (Fig. 6b–d). All five CD276 minibinder CARs selectively recognized CHO cells overexpressing human CD276 (CD276^oe^), though HM9 CAR-T cells were also activated by control CHO cells (Supplementary Fig. 9a–c). Since HM9 was the only minibinder that cross‑reacted with murine CD276 (Supplementary Fig. 9d), we believe that HM9 CAR-T cell recognition of wild-type CHO cells is a cross-species target‑specific binding rather than an off‑target effect. This conclusion is further supported by the structural models showing that all sequence differences between murine and hamster (*C. griseus*) CD276 lie outside the predicted binding interface (Supplementary Fig. 9e).

We then co‑cultured the various CD276 minibinder CAR-T cells with human A375 melanoma cells, which endogenously express CD276. In addition, we used CRISPR-Cas9 to generate matched A375 CD276 knockout (KO) cells and re-expressed human CD276 (CD276^re^) at levels higher than endogenous CD276 expression in A375 wild-type (WT) cells (Supplementary Fig. 9f). All five CD276 minibinder CAR-T cells were selectively activated by re-expressed CD276 on A375 CD276 KO cells (Fig. 6e, f), though mb and HM9 CAR-T cells produced less IFN-γ (Fig. 6f). Notably, only mb and HM9 CAR-T cells responded to endogenously expressed CD276 on A375 WT cells by CD25 upregulation and IFN-γ production, presumably due to their high binding affinity as previously reported^30^. Overlaying the structural models showed that our CD276 AI‑minibinders and HM9 occupy largely the same binding site on CD276 (Fig. 6g–i). From these findings, we conclude that deploying AI‑minibinders in CAR-T cell platforms requires careful evaluation of multiple factors, in particular target binding affinity and efficient cell‑surface presentation. In this context, incorporating mammalian cell‑surface display during binder discovery helps to select AI‑minibinders with the biochemical profiles needed for effective expression on CAR‑T cells.

### Minibinder isoelectric point determines CAR surface accessibility

**Fig. 7:**
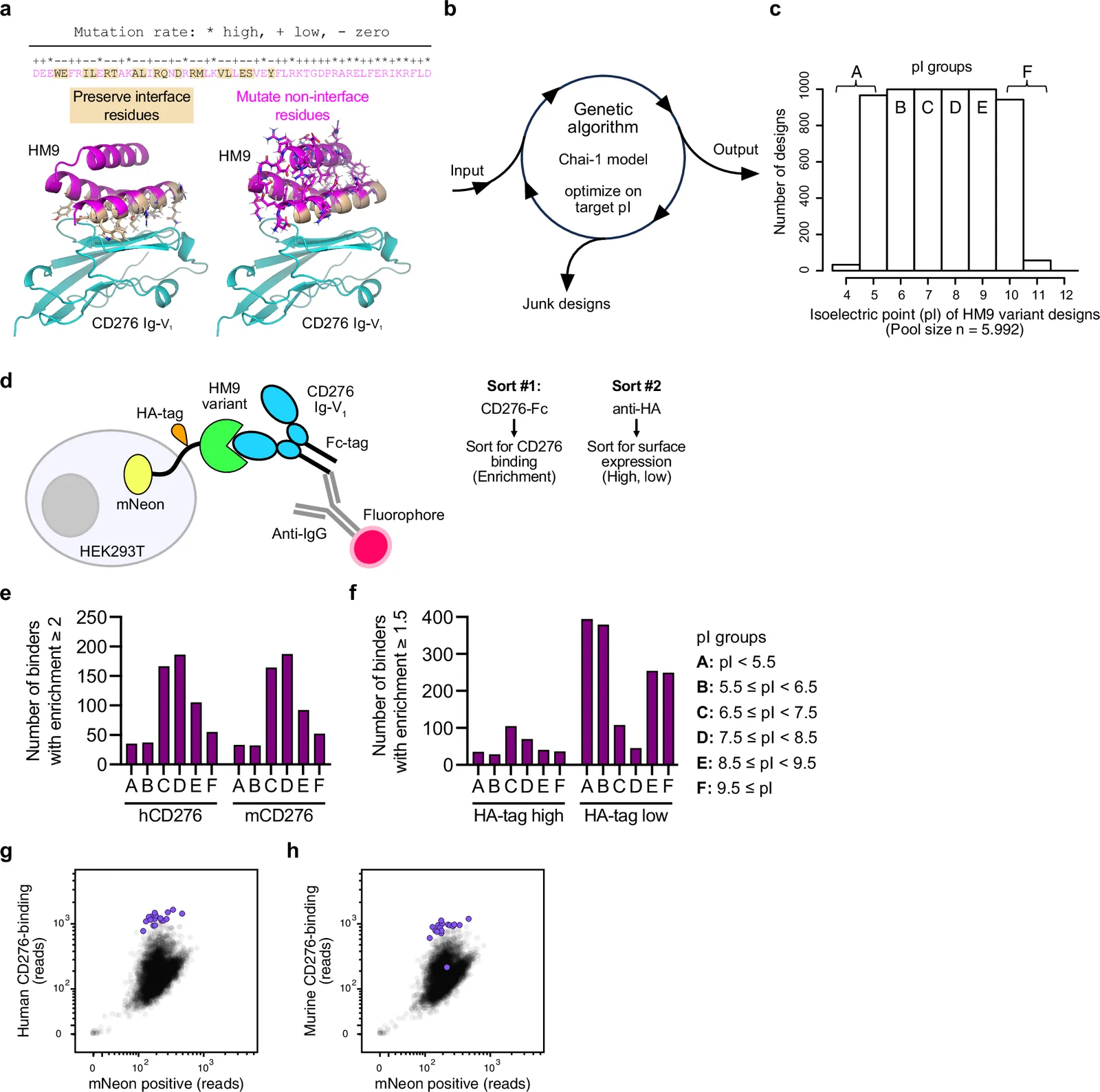
Optimal cell surface trafficking of AI-minibinder CARs is dependent on their isoelectric point. **a** Outline of computational design strategy to generate HM9 variants mutating only non-interface residues. The mask identifies residues to be preserved or mutated at different rates (^+^5%, *10%). **b** Cartoon showing principle of genetic algorithm based on the Chai-1 model with ESM2 protein LLM embedding as described in Tan et al.^39^. **c** HM9 variant pool (*n* = 5.992 designs) composition according to isoelectric point (pI). **d** Graphical summary of mammalian cell-surface display screen of the HM9 variant pool with sorting for CD276 binding and minibinder surface expression (CD276 Fc-fusion protein produced in HEK293 cells). Cartoon generated using Affinity Publisher 2. **e** CD276-binding HM9 variants identified by NGS per pI-value group based on at least 2-fold enrichment. **f** Same experiment as e but cells were sorted for high or low HA-tag staining on the cell surface and binders were counted with at least 1.5-fold enrichment. Scatter plots showing enriched HM9 variant CD276 minibinders identified by NGS analysis of subpopulations binding human CD276 (**g**) or murine CD276 (**h**). Selected minibinders evaluated in further experiments are marked in purple. Source data are provided as a Source Data file.

Although our in-house designed CD276 minibinders and the control minibinders targeted a similar site on CD276, they behaved differently in CAR-T cells, prompting us to investigate underlying biochemical differences. We found significant differences in the minibinders’ isoelectric points (pI) with HM9 and mb at 9.4 and 9.8, respectively, and our binders ranging from 4.7 to 5.2 while demonstrating better surface presentation (Supplementary Fig. 10a). Because the pI values of the minibinders spanned such a broad range, we decided to systematically investigate the role of minibinder pI in CAR cell-surface trafficking and function. Owing to the small size of minibinders, even single amino acid substitutions can substantially affect biochemical properties such as pI and hydrophobicity (GRAVY score). To this end, we applied our recently developed genetic algorithm employing the Chai-1 model with ESM embeddings^39^. As the input template, we used the CD276 minibinder HM9, which showed poor surface expression as CAR, fixed residues at the CD276-binding interface, and allowed non-interface residues to mutate (Fig. 7a). The algorithm uses a positional mask to specify which amino acid positions are mutable during sequence editing and can incorporate target pI values as an input parameter, enabling iterative evolutionary selection to enrich variants approaching the desired pI (Fig. 7b). This way, we generated a pool of nearly 6000 HM9-derived minibinder variants spanning a broad and evenly distributed pI range (Fig. 7c, Supplementary Fig. 10b, c, Supplementary Data 11).

We then used mammalian cell-surface display to screen for binding to human or murine CD276 (Fig. 7d) and additionally sorted for high versus low surface expression based on the HA-tag signal. Notably, despite preserving the binding interface and mutating only non-interface residues, we observed an unexpectedly low proportion of cells (~15–17%) positive for CD276 staining, indicating a strong influence of non-binding interface properties (Supplementary Fig. 10d). Motivated by this observation, NGS analysis of the CD276-binding cell population revealed strong enrichment of HM9 variant minibinders with pI values between 6.5 and 8.5 (pI groups C and D; Fig. 7e). Consistent with this, minibinders from these pI groups were rarely represented in the population with low HA-tag expression, showing that minibinders within the pI range of 6.5–8.5 are generally well expressed at the cell surface (Fig. 7f). Based on the enrichment plots for human CD276 binding, 17 HM9 variant minibinders were selected for further functional analyses in CAR-T cells (Fig. 7g, h).

### Minibinder isoelectric point influences target selectivity

**Fig. 8:**
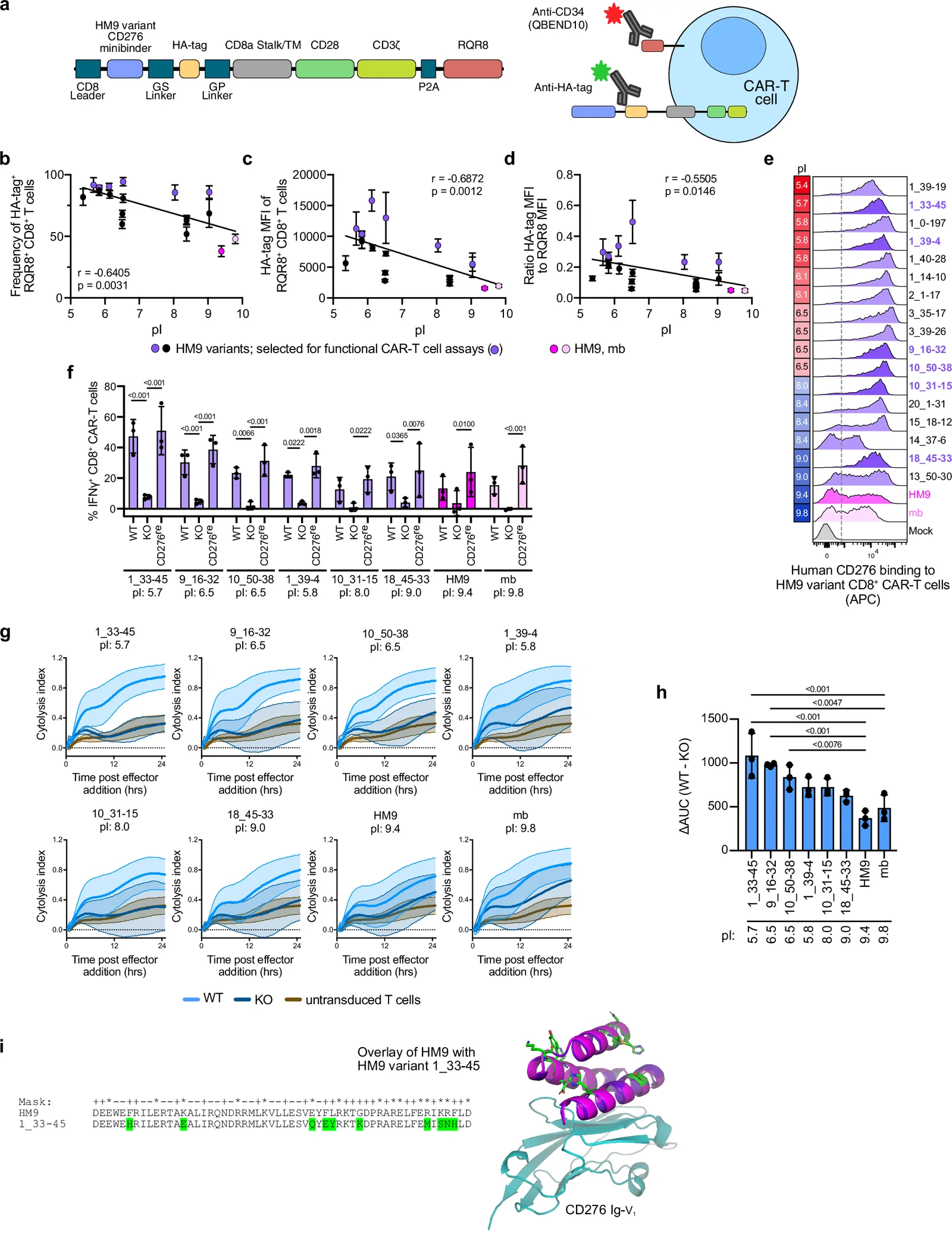
Isoelectric point optimization improves activity and selectivity of AI-minibinder CAR-T cells. **a** Outline of deployed CAR construct and flow cytometric strategy to assess transduction efficiency (RQR8 via anti-CD34 antibody) and CAR cell surface expression (HA-tag) on human T cells. Cartoon generated using Affinity Publisher 2. **b**–**d** Correlation of pI-values of selected HM9 variant CD276 minibinders with surface expression of the respective CAR on transduced (RQR8^+^) human T cells. Assessment of the frequency of HA-tag^+^ cells (**b**), the geometric mean fluorescence intensity (MFI) of the HA-tag (**c**), and the ratio of the MFIs of the HA-tag to RQR8 (normalized to expression, **d**). Binders used in functional assays are marked in purple. Experiments performed in biological triplicates (*n* = 3). r, Spearman’s correlation value. Lines indicate simple linear regression analysis. **e** Representative flow cytometric analysis showing the binding patterns against human CD276 of selected HM9-derived CD276 binders as CARs on transduced human CD8^+^ T cells. Respective pI-values are indicated on the left. **f** Quantification of IFN-γ induction in indicated CD276 AI-minibinder CAR-T cells co-cultured (1:1 E:T ratio) for 5 h with human A375 melanoma cells endogenously expressing CD276 (WT), CRISPR-Cas9-engineered CD276 knockout (KO) cells, or reconstituted CD276 KO cells (CD276^re^), respectively (*n* = 3 PBMC donors). **g** Representative graphs showing cytolysis indices derived from CD276 AI-minibinder CAR-T cells co-cultured for 24 h with target cells (1:1 E:T ratio) as described in (**f**) using the impedance-measurement-based xCELLigence platform. High cytolysis indices indicate cell death. **h** Quantification of g in biological triplicates (*n* = 3). The area under the curve (AUC) was calculated for each treatment and target cell condition to subtract the value of CD276 KO cells from the respective CD276 WT cell condition (ΔAUC). **i** Sequence alignment and structural model overlay (Chai-1) of HM9 (magenta) and top candidate variant HM9 1_33-45 (blue). Variant residues are highlighted in green. Two-way ANOVA with Šídák’s multiple comparisons test. Error bars indicate mean ±SD. Source data are provided as a Source Data file.

To determine whether pI optimization improved the performance of AI-minibinder CARs, we selected HM9-derived CD276 minibinder variants spanning a range of isoelectric points and expressed them as CARs on primary human T cells (Fig. 8a). CAR surface accessibility on transduced (RQR8^+^) human T cells strongly correlated with minibinder pI, as reflected by the frequency of HA-tag-positive cells (Fig. 8b), the geometric mean fluorescence intensity (MFI) of the HA-tag (Fig. 8c), and the HA-tag-to-RQR8 MFI ratio, which normalizes surface accessibility to overall expression levels (Fig. 8d). In particular, variants with more basic pI values tended to show impaired CAR surface presentation. As expected from the selection strategy, all variants retained binding to human CD276 when expressed in the CAR format (Fig. 8e). However, the highly basic minibinder variants displayed weaker target binding, in line with their reduced surface expression. In addition, their broader flow-cytometric staining profiles indicated greater heterogeneity within the CAR-T cell population, further supporting the notion that highly basic minibinders are less efficiently and less uniformly presented at the cell surface.

Next, we assessed the functional consequences of these differences in co-culture experiments using A375 melanoma cells with endogenous CD276 expression (WT), CRISPR-Cas9-engineered CD276 knockout (KO) cells, or reconstituted CD276-expressing cells (CD276^re^). The pI optimized HM9-derived variants induced stronger IFN-γ and TNF-α responses than the parental HM9 CAR upon exposure to CD276-positive target cells, while remaining largely inactive against CD276 knockout cells (Fig. 8f, Supplementary Fig. 10e). Impedance-based killing assays likewise demonstrated enhanced cytotoxicity of optimized variants against CD276 WT compared to CD276 KO melanoma cells (Fig. 8g). Untransduced T cells induced minor target cell killing, potentially due to baseline activation, target cell sensitivity to cytokines, and minor alloreactive effects. Notably, the highly basic minibinders showed a reduced selectivity window, as reflected by less pronounced discrimination between killing of WT and CD276 knockout target cells. Variants within the more favorable pI range combined stronger activity with improved target selectivity (Fig. 8h). Together with the experimental data, sequence alignment and structural modeling identified HM9-variant 1_33-45 as a top candidate carrying a defined set of non-interface substitutions (Fig. 8i). Finally, this supported the concept that tuning biochemical properties outside the binding interface can be critical to improve CAR surface accessibility, activity, and selectivity without compromising antigen recognition.

## Discussion

Recent advances in AI-based protein design open new opportunities for biomedical research^7–10,26^, including the development of research tools and next-generation therapeutics. We established a workflow that integrates de novo minibinder design with pooled experimental screening in formats directly relevant to biological applications. This enabled the generation and validation of minibinders against multiple cancer surface targets of the B7-H family and their evaluation as both, open-access detection reagents for flow cytometry and antigen-recognition domains in CAR-T cells. Our experimental success rates for PD-L1 minibinders, for example, aligned well with those reported previously for RFdiffusion^7^.

In our hands, RFdiffusion-based design was markedly more efficient for PD-L1 than for the related B7 family members CD276 and VTCN1. Given that RFdiffusion performs best when targeting hydrophobic hotspot patches, one possible explanation is that PD-L1 presents a more prominent and accessible hydrophobic binding interface for minibinders compared to CD276 and VTCN1, based on available structures and models. Notably, the PD-L1–PD-1 interaction is substantially stronger than most other immunoglobulin superfamily (IgSF) interactions, while natural ligands for CD276 and VTCN1 remain controversial^40,41^. In this context, the observed differences in design efficiency may reflect intrinsic properties of PD-L1, including an interface optimized for high-affinity interaction with PD-1, particularly as the binding interface of our minibinders overlaps with the PD-1 interaction site.

Beyond demonstrating the utility of available protein design tools for the broader research community, our study provides a systematic assessment of the requirements for translating AI-designed minibinders into functional molecules. In particular, our data demonstrate that target binding alone is insufficient, as a minibinder must also detect endogenous protein levels with sufficient sensitivity, reliably discriminate between wild-type and knockout cells, and retain selectivity and robustness in its intended application format. Based on our experience, CRISPR-Cas9-engineered isogenic pairs of target-proficient wild-type and target-deficient knockout cells should be incorporated into every minibinder validation campaign. Ideally, multiple independent isogenic cell line pairs should be used, as we observed cases in which a minibinder showed clean target-dependent staining in one pair but nonspecific background staining in another. These observations highlight the necessity of rigorous experimental quality control and emphasize that de novo designed minibinders must be carefully benchmarked similar to conventional antibodies^31^ before they can be considered reliable reagents or even therapeutic leads.

In our study, the need for rigorous quality control and minibinder optimization became particularly evident in the CAR-T cell setting. Although the previously reported high-affinity CD276 minibinder HM9 and our in-house designed minibinders target the same epitope, they behaved differently when expressed as CARs with regard to surface expression and detection of endogenous protein levels. To investigate this systematically, we employed our recently developed genetic algorithm^39^ based on the Chai-1 model with ESM embedding^26^. This approach enables sequence editing of non-interface residues while preserving the binding interface and allows to specify desired pI-values as an input parameter. By generating an extensive library of nearly 6000 HM9-derived variants, we experimentally validated that the isoelectric point is a key determinant of CAR surface accessibility, activity, and selectivity. The identified favorable pI range of 6.5–8.5 is also consistent with a human proteome-wide analysis of extracellular and plasma membrane proteins^42^. Of note, re-analyzing our PD-L1, CD276 and VCTN1 minibinder pools designed with RFdiffusion, we observed a bias towards acidic pI values (pI <7, 2.3 times more frequent) linked with a trend towards functional binding (odds ratio = 1.97, *p* = 0.096, Fisher’s exact test). CARs with highly basic minibinder pI values exhibited reduced target selectivity, potentially reflecting increased nonspecific electrostatic interactions with negatively charged cell surface components, which may contribute to apparent off-target cytotoxicity in addition to reduced surface expression. Together, our findings highlight the non-binding interface as a critical and still insufficiently understood dimension of AI-minibinder optimization. More broadly, they demonstrate that a high-affinity minibinder is not necessarily a lead candidate, as biochemical properties beyond target recognition can strongly influence intracellular trafficking, surface presentation, and downstream function in complex therapeutic formats such as CAR-T cells. In addition, glycosylation of AI-minibinders represents an important parameter that could be rationally incorporated during the design to further modulate stability, trafficking, and function. Of note, recent work by other groups has also examined determinants of minibinder performance in CAR formats, further underscoring the importance of this optimization step^43,44^. Thus, our work defines key determinants of CAR surface accessibility and supports the further evaluation of AI-designed minibinders for therapeutic applications.

A limitation of our study is that we do not provide in vivo validation of the minibinder-based CAR-T cells. However, proof-of-concept that de novo-designed minibinders can serve as CAR recognition domains in vivo has already been established by Xia et al., who demonstrated the activity of EGFR- and CD276-targeting minibinder (HM9) CAR-T cells in xenograft models in immunodeficient mice^30^. The most important next step is therefore to advance toward syngeneic models that can directly address the key question of minibinder immunogenicity, since these artificial proteins may be recognized as foreign by the immune system, and determine how this affects CAR-T cell persistence, tumor selectivity and on-target/off-tumor toxicity^45^. Optimal CAR function requires sufficient sensitivity toward tumor cells while avoiding reactivity against physiological antigen expression in normal tissues. Therefore, the highest-affinity minibinder may not necessarily confer the most favorable therapeutic window. In the case of CD276-targeting minibinders, such studies would require rigorous experimental designs that compare CD276-proficient and CD276-deficient tumors in both CD276-proficient and CD276-deficient hosts. This is particularly important since CD276 is expressed not only on tumor cells but also on other host cell populations, including tumor-associated vasculature^46^, making it necessary to distinguish tumor-cell-directed activity from effects mediated through stromal or vascular targeting. Conditional mouse models with cell-type-selective CD276 deletion could further dissect the contribution of stromal and vascular target expression. In addition, CD276 minibinders with distinct pI values would need to be compared side by side to determine how this biochemical parameter influences the therapeutic window, including CAR-T cell activity, persistence, and on-target/off-tumor effects. These considerations highlight the need for dedicated in vivo studies to define the above-mentioned therapeutic aspects of optimized minibinder-based CAR-T cells. Thus, rather than presenting our minibinder-based CARs already as optimized therapeutic candidates, our work highlights important parameters and potential caveats that should be considered when adapting AI-designed binders to CAR formats or other mammalian cell-based applications.

Alternative binding formats such as camelide-derived variable domains of heavy-chain only antibodies (VHH, also known as nanobodies) or DARPins (Designed Ankyrin Repeat Protein) have also been explored for CAR-T cell engineering^37,47,48^, offering smaller size compared to conventional scFvs, although they are generally larger than de novo minibinders. Interestingly, comparing the binding interface area of 15 different VHHs with Ig-like V-type domains (PDB IDs: 5e5m, 5iml, 5jds, 6rpj, 6rqm, 7czd, 8aok, 8aom, 8ew6, 9ezh, 9ezi, 9ezu, 9ezv, 9ezw, 9lme) showed that minibinders reported in our study may be capable of forming binding interfaces that are comparable to, or even larger than, those of VHHs despite their smaller size (mean VHH: 735 Å² ± 73; mean minibinder: 871 Å² ± 93; two-sided *t*-test, 1.11 × 10⁻⁶). On the other hand, established humanization strategies for nanobodies may provide advantages in terms of immunogenicity, whereas the immunogenic potential of minibinders will need to be carefully evaluated in future studies. Ciltacabtagene autoleucel is a VHH-based CAR-T cell therapy approved for the treatment of adult patients with relapsed and refractory multiple myeloma targeting B-cell maturation antigen^49^.

Poorly performing commercial antibodies remain a persistent problem despite ongoing efforts to improve reagent validation^31–33^. In this context, AI-designed protein binders offer an attractive opportunity, as their sequences and structural models are openly accessible and can be iteratively improved by the research community. They can be produced cost-effectively at high yield in microbial systems, and their small size enables rational design with defined binding interfaces. Moreover, their stability, including resistance to thermal stress, may facilitate improved shelf-life and handling. Together, these properties suggest that minibinders could serve as useful alternatives for applications such as flow cytometry. Our PD-L1 quattrobinder data provide proof of principle that AI-designed minibinders can perform on par with conventional antibodies in flow cytometry. However, the variability observed across targets, including limitations in specificity or sensitivity in certain settings, underscores the importance of stringent validation and further refinement.

In addition to RFdiffusion^7^, several recently developed design pipelines, including BindCraft^9^ and AlphaProteo^8^, report encouraging experimental validation rates for de novo binders. Such methods are likely to expand the range of accessible targets, particularly when combined with downstream functional screening. As experimentally characterized AI-minibinder datasets continue to grow, they will become an invaluable resource not only for benchmarking design algorithms but also for improving the prediction of biochemical features that determine deployability in specific application formats.

In summary, our study shows that AI-designed minibinders can be translated into useful open-access research reagents for flow cytometry or CAR-T cells, while also defining important current limitations. In particular, we identify biochemical properties, especially the isoelectric point, as key determinants of CAR surface accessibility and target selectivity. Together, these findings show that the non-binding interface of AI-designed proteins represents an important design space and that systematic functional profiling will be essential for converting AI-generated binders into robust biomedical reagents and therapeutics.

## Methods

### Ethics

All study procedures were performed in compliance with relevant laws and institutional guidelines and have been approved by the Ethics Committee of the Medical Faculty of the University of Bonn (Amendment to Az.285/21). Written informed consent was received prior to participation from the blood donors. Peripheral blood from healthy donors was provided by the Institute for Experimental Hematology and Transfusion Medicine at the University Hospital Bonn, Bonn, Germany. All procedures involving the use of mouse splenocytes were conducted in accordance with the German Animal Welfare Act (TierSchG) and all applicable ethical regulations and institutional guidelines. C57BL/6 mice that had not undergone any prior experimental procedures or treatments were euthanized solely for spleen collection, with death confirmed before organ removal; this procedure did not constitute an animal experiment requiring project-specific authorization and was performed, documented, and included in the annual statutory reporting in accordance with Section 4(3) TierSchG.

### Initial AI-minibinder design pipeline

Our AI-minibinder design pipeline was implemented as a series of chained command-line steps (bash script). For each design job, an output directory was created and the input PDB, configuration file, and user-specified parameters (number of designs, countig sequences, hotspot residues) were set. RFdiffusion was then used with default parameters according to the instructions (https://github.com/RosettaCommons/RFdiffusion) to generate de novo poly-Gly backbone models. The DSSP program from the CCP4 suite^19,20^ was used to filter the designs and only structures with three or more helices were funneled into the next steps of the pipeline. PMPNN was used to design two alternative interface sequences for each poly-Gly backbone (https://github.com/nrbennet/dl_binder_design). Each design was scored with AlphaFold2 initial guess and only designs with a pAE interaction score <10 were used in downstream experiments.

### AI-minibinder design pipeline using a genetic algorithm

A detailed description of the genetic algorithm (GA), which optimizes de novo designed minibinders based on basic principles of evolutionary selection, has been recently released^39^. HM9 variant minibinders were refined in silico using GA based on iterative fitness scoring, tournament selection, and diversification across multiple generations (*n* = 50). Diversification was achieved by direct sequence-level editing using Chai-1 with ESM embedding^26^. Fitness was evaluated using predicted structural and interface confidence metrics (ipTM) and could be supplemented with user-defined constraints, including isoelectric point or hydrophobicity (GRAVY score). Whether residues were allowed to mutate at higher (10%) or lower (5%) rate was defined by a provided mask (Fig. 7a). RFdiffusion partial diffusion followed by ProteinMPNN re-design is an additional option of the GA, which was not used in this study. GA-based HM9 variant CD276 minibinder refinement was computed on the Marvin HPC at the University of Bonn, Germany. Per GA computational run, we used one node of the scalable GPU applications (SGPU) partition with the following specifications: CPU: 2× AMD EPYC “Milan” 64-core/128-thread 2.00 GHz; RAM: 1024GB DDR4 3200 MHz; GPU: 4× NVIDIA A100 80GB (connected via NVLink within the node).

### Input pdb files or AlphaFold models

Target protein structures for RFDiffusion design were crystal structures: PD-L1 (PDB: 4zqk), VTCN1 (PDB: 4gos), or AlphaFold models: CD276 (Q5ZPR3-F1-model_v4, July 2023).

### Oligonucleotide pool design

Using our online pool design tool (jsb-lab.bio/AI-binders), AI-minibinder sequences were reverse-translated with random codon selection, omitting rare human codons and excluding randomly occurring Esp3I recognition sites. Overhangs, Esp3I sites, and common primer binding sites were added, and oligonucleotide sequences were filled up to the maximum oligonucleotide length using random DNA bases. Amino acid sequences, reverse translated cDNA sequences, and ordered DNA oligo sequences of PD-L1, CD276, and VCTN1 minibinders from the pilot pool, improved pool, and HM9 pool are provided in Supplementary Data 2, 5, and 10, respectively. Amino acid sequences, reverse translated cDNA sequences and ordered DNA oligo sequences of all minibinders including CD276 minibinder HM9 variants studied in greater detail are provided in Supplementary Data 12. Asparagine (N) residues and potential N-X-S/T glycosylation motifs are highlighted.

### DNA sequences

Primer sequences are provided in Supplementary Data 8.

### Phage display

To generate a library for phage display, the AI-minibinder library was amplified by PCR to introduce NotI and AscI restriction sites (primers fwd_NotI, rev_AscI) and the AI-minibinder library was cloned into a phagemid vector. The phage display was done using *E. coli* TG1 cells in combination with VCSM13 helper phages to enrich specific AI-minibinders. After phage production and purification by PEG/NaCl precipitation, the phages were precleared on magnetic streptavidin beads (Thermo Fisher, 6560). The bait proteins (human proteins produced in HEK293 cells) were biotinylated by ChromaLink Biotin (Solulink, B-1007-110) and immobilized to streptavidin beads. Two rounds of panning were performed using 20 µg and 2 µg of target protein for round one and two, respectively. *E. coli* ER2738 cells were infected with the enriched phages and plated on LB-agar with 2YT/2% (w/v) glucose/Amp/Tet. Glycerol stocks were prepared of all libraries and plasmid mini-purification was performed. Plasmid DNA of input, as well as output phages from two panning rounds were used as template for NGS library generation (primers NGS_phages_fwd and NGS_phages_rev, followed by a second PCR using Illumina barcode primers) and sequencing using the Illumina MiSeq platform.

### Library cloning for mammalian cell-surface display screening

The oligo pool (Twist Biosciences) was PCR amplified (primers pool-amplification_fwd, pool-amplification_rev) and cloned into the lentiviral target vector using Golden Gate assembly with the restriction enzyme Esp3I. The assembly was purified using a DNA Clean & Concentrator-5 column (Zymo) and was electroporated into 25 µL Endura electrocompetent *E. coli* (Biosearch Technologies) in 0.1-cm Bio-Rad cuvettes at 1800 V, 10 µF, and 600 Ω. Cells were resuspended into 975 µL recovery medium and incubated for 1 h at 37 °C, 300 rpm. The suspension was then transferred into 450 mL LB medium containing 100 µg/mL ampicillin and grown overnight at 37 °C, 180 rpm. Plasmid DNA was purified using the PureLink HiPure Plasmid Midiprep Kit (Invitrogen). AI-minibinder coverage of the plasmid library was determined by PCR amplification (primers NGS_cell-display_fwd and NGS_cell-display_rev, followed by a second PCR using barcode primers) and sequencing using the Illumina MiSeq platform.

### Library transduction for mammalian cell-surface display screening

With HEPES-buffered saline (HBS) and CaCl_2_, 1 × 10^6^ HEK293T cells were transiently transfected with the AI-minibinder plasmid library and the lentiviral packaging plasmids pMD2.G (Addgene, 12259) and psPAX2 (Addgene, 12260) at 2 µg, 0.65 µg, and 0.35 µg, respectively. The medium was changed 16 h and 24 h after transfection. 40 h post-transfection, the medium was collected and filtered through a 0.45 µm syringe filter. 2 × 10^6^ HEK293T cells were transduced with the filtered, virus-containing supernatant at a 1:10 dilution aiming at transduction rates <60%. Transduced cells were selected by supplementing the medium with 1 µg/mL puromycin.

The HEK293T AI-minibinder library was harvested and incubated with respective Fc-tagged recombinant target proteins at 10 µg/mL (Supplementary Data 9) and secondary APC-labeled anti-human IgG Fc antibody at 250 ng/mL (clone M1310G05, Biolegend). Cells were sorted immediately after staining for distinct APC-signal using the fluorescence-activated cell sorter Sony MA900. Sorted cells were lysed in direct lysis buffer (1 mM CaCl_2_, 3 mM MgCl_2_, 1 mM EDTA, 1% (v/v) Triton X-100, 10 mM Tris pH 7.5) with proteinase K at 65 °C for 10 min and 95 °C for 15 min. Genomically integrated AI-minibinder sequences were PCR amplified using 2× NEBNext High-Fidelity MasterMix (NEB) using primers NGS_cell-display_fwd and NGS_cell-display_rev. After second barcoding PCR (barcode primers), product purification, and NanoDrop-based quantification, AI-minibinders were sequenced on Illumina MiSeq or NextSeq platforms. Reads were filtered for a Q-Score > 30 and matched to AI-minibinder sequences. AI-minibinder counts were subjected to enrichment analysis (https://jsb-lab.bio/ai-binders/).

### Individual AI-minibinder cloning and cell-surface display validation

AI-minibinder sequences were ordered as individual gene fragments (Twist Biosciences) and cloned into the lentiviral target vector using Golden Gate assembly with the restriction enzyme Esp3I. Plasmid sequences were verified by Sanger sequencing (Microsynth).

HEK293T cells were transiently transfected with the AI-minibinder expression constructs using HBS and CaCl_2_. 36–48 h post-transfection, cells were harvested and incubated with respective Fc-tagged target proteins at 10 µg/mL (Supplementary Data 9) and secondary APC-labeled anti-human IgG Fc antibody at 250 ng/mL, or with APC-labeled anti-HA.11 epitope tag antibody (clone 16B12, Biolegend). Cells were acquired at the analyzers BD FACSCanto II or 4-laser Cytek Aurora Spectral Flow Cytometer, and analyzed using FlowJo. For statistical analysis, the geometric mean of the APC-signal MFI of protein binding was normalized to the respective HA.11 epitope tag APC-signal to account for differences in surface presentation.

### Cell culture

The human cell lines HEK293T (embryonic kidney, CVCL_0063), FTC-133 (thyroid carcinoma, CVCL-1219, kindly provided by Markus Essler, Nuclear Medicine, UKB), U87MG (glioblastoma, CVCL_0022), and their derivates were cultured in Dulbecco’s Modified Eagle Medium (DMEM) - GlutaMAX^TM^ medium (Gibco) supplemented with 10% (v/v) heat-inactivated FBS (Gibco) and 100 U/mL Penicillin-Streptomycin (Gibco). The human cell lines 5637 (bladder carcinoma, CVCL_0126), A375 (melanoma, CVCL_0132), the Chinese hamster cell line CHO-K1 (ovary, CVCL_0214), and their derivates were cultured in Roswell Park Memorial Institute Medium (RPMI) 1640 - GlutaMAX^TM^ medium (Gibco) supplemented with 10% heat-inactivated FBS (Gibco) and 100 U/mL Penicillin-Streptomycin. Platinum-E (Plat-E) cell lines were cultured in DMEM - GlutaMAX^TM^ medium, 10% (v/v) heat-inactivated FBS, 10 mM MEM Non-Essential Amino Acids Solution (Gibco), 100 U/mL Penicillin-Streptomycin, 1 mM Sodium Pyruvate (Gibco), and 10 mM HEPES (Roti). Primary human and murine cells were cultured in RPMI complete comprising RPMI 1640 - GlutaMAX^TM^ medium, 10% heat-inactivated FBS, MEM Non-Essential Amino Acids Solution 1X (Gibco), 100 U/mL Penicillin-Streptomycin, 1 mM Sodium Pyruvate (Gibco), 10 mM HEPES (Roti), and 1X β-mercaptoethanol (Gibco), as well as below indicated additional experiment-specific supplements. All cell lines were cultured at 37 °C in 5% CO_2_. Identity of all cell lines used in this study was confirmed by re-authentication using short tandem repeat (STR) profiling. All cell lines were routinely tested for mycoplasma by PCR and confirmed to be negative.

### Knockout (KO) cell line generation and validation

DNA templates for sgRNAs (Microsynth) against *CD274*, *CD276*, or *VTCN1* were cloned into the CRISPR-Cas9 target vector px458 (Addgene, 48138) using Golden Gate assembly with the restriction enzyme BbsI. Plasmids were transfected into respective cell lines using FuGENE transfection reagent (Promega; 3:1 FuGENE reagent to DNA). 7 to 10 days post-transfection, cells were harvested and stained using fluorescently tagged antibodies against PD-L1 (clone IPI652.rMAb, BD Biosciences), CD276 (clone 185504, R&D Systems), or VTCN1 (clone MIH43, Biolegend), respectively. Cells were sorted for surface protein negative knockout phenotype using the fluorescence-activated cell sorter Sony MA900. Target sequences for sgRNA constructs were: *CD274*, CGCTGCATGATCAGCTATGG; *CD276*, CAGCGCCTATGCCAACCGCA; and *VTCN1*, TCGGGGAGCCTCACACCGCA.

### Generation of overexpression cell lines

Overexpression cell lines were created for the proteins PD-L1 (human Q9NZQ7-1, residues 1–290), CD276 (human Q5ZPR3-1, residues 1–534), and VTCN1 (human Q7Z7D3-1, residues 1–282). Gene fragments (Twist Biosciences) were cloned into the pRP233 or pRP236 target vectors (derived from Addgene plasmid #41841). HEK293T cells were transiently transfected with the protein expression construct and the retroviral packaging plasmids Gag-Pol and VSV-G at a ratio of 3:2:1. The medium was changed after 16 h and 24 h. 40 h post-transfection, the virus-containing medium was collected, filtered through a 0.45 µm syringe filter, and immediately used for the transduction of CHO-K1, 5637, and A375 cells. Generated overexpression cell lines were sorted for purity using the fluorescence-activated cell sorter Sony MA900. cDNA sequences cloned into pRP233 or pRP236 vectors are listed in Supplementary Data 8.

### Cloning of AI-minibinders for expression in *E. coli*

For expression of AI-minibinders in *E. coli*, coding sequences were subcloned from the respective lentiviral vector into the pEHISTEV vector^50^, which contains an N-terminal hexahistidine tag, followed by a tobacco etch virus (TEV) protease cleavage site. Cysteine point mutations were introduced into plasmids by primer-directed mutagenesis. For constructs with low expression yield, the coding sequences were codon-optimized for *E. coli* K-12 substr. MG1655 using the VectorBuilder Codon Optimization Tool (https://en.vectorbuilder.com/tool/codon-optimization.html) and synthesized as gene fragments (Twist Biosciences).

### Expression and purification of AI-minibinders

Recombinant 6xHis-tagged AI-minibinders were expressed in *E. coli* BL21 (DE3) bacterial cells grown in LB medium containing 50 µg/mL kanamycin at 37 °C for 16 h (pre-culture). The following day, larger culture volumes were inoculated with the pre-culture and adjusted to an optical density (OD_600_) of 0.1–0.2. Cultures were grown at 37 °C to an OD_600_ of 0.8 and protein expression was induced by addition of IPTG to a final concentration of 0.5 mM. Proteins were expressed at 18 °C for 16 h. Bacteria were harvested by centrifugation at 4000 × *g* for 25 min. Cell pellets were flash-frozen in liquid nitrogen and stored at −20 °C or subjected to immediate cell lysis.

Cell pellets were resuspended in lysis buffer (20 mM Tris pH 8.0, 500 mM NaCl, 20 mM imidazole) and lysed by sonication. Cell debris was sedimented by centrifugation at 25,000 × *g* for 45 min at 10 °C. The lysate was filtered through a membrane filter with a 0.8 µm pore size and subjected to affinity chromatography.

For affinity chromatography, Ni^2+^-NTA resin was equilibrated with lysis buffer and incubated with lysate for at least 2 h at 4 °C or room temperature on a rolling incubator. The resin was transferred to a gravity column and washed extensively with lysis buffer and eluted with elution buffer (20 mM Tris pH 8.0, 500 mM NaCl, 500 mM imidazole). For removal of the 6xHis-tag, the elution fraction was dialyzed against dialysis buffer (20 mM Tris pH 8.0, 250 mM NaCl) for 16 h at 4 °C and supplemented with 7xHis-tagged tobacco etch virus (TEV) protease (1:20 w/w) for 16 h at 4 °C. For cysteine mutants, all buffers were additionally supplemented with 5 mM β-mercaptoethanol.

For further purification following affinity chromatography or TEV digest, the AI-minibinders were subjected to size exclusion chromatography (SEC) on a HiLoad 16/600 Superdex 75 pg column (Cytiva) equilibrated in dialysis buffer. For SEC runs of cysteine mutants, the dialysis buffer was supplemented with 1 mM Tris(2-carboxyethyl)phosphine (TCEP). For TEV-digested proteins, the SEC column was equipped with an additional 5 mL HisTrap column (Cytiva) for removal of residual tag, non-cleaved protein and TEV protease.

### Side-directed biotinylation of AI-minibinders

AI-minibinders containing cysteine point mutations were biotinylated using the maleimide-reactive PEG_2_-Biotin label (EZ-Link, ThermoScientific). Prior to biotinylation, AI-minibinders were incubated in dialysis buffer containing 2 mM TCEP for 60 min at room temperature to reduce potential disulfide bonds. Immediately before addition of the biotin label, TCEP was removed by buffer exchange into labeling buffer (20 mM Tris or 20 mM HEPES pH 7.0, 250 mM NaCl) using 7 K MWCO Zeba Spin desalting columns (ThermoScientific). The label was added at a 10-fold molar excess and incubated overnight at 4 °C. Excess label was removed by buffer exchange into labeling buffer using 7 K MWCO Zeba Spin desalting columns.

### Quattrobinder assembly and flow cytometry staining

*E. coli* produced and biotinylated AI-minibinders were step-wise mixed and incubated with Alexa Fluor® 647-conjugated Streptavidin (Invitrogen) at a molar ratio of 4:1. Assembled quattrobinders were stored at −80 °C. For staining procedures, target cells were harvested and incubated with Zombie Violet™ Fixable Viability Kit (Biolegend) at 1:1000 in PBS and quattrobinders at 150 nM in FACS buffer (PBS, 2% (v/v) FBS, 2 mM EDTA). Samples were acquired at the analyzers BD FACSCanto II or 4-laser Cytek Aurora Spectral Flow Cytometer, and analyzed using FlowJo software (BD Biosciences).

### Surface plasmon resonance measurements

Surface plasmon resonance (SPR) measurements were performed on a Series S CM5 sensor chip (Cytiva) using a Biacore™ 8 K instrument (GE Healthcare) operated at 25 °C. The chip was equilibrated in running buffer (20 mM HEPES pH 7.4, 150 mM NaCl, 1 mM TCEP, 0.05% (v/v) Tween-20). Human PD-L1 extracellular domain (ECD) containing a C-terminal LPETG-tag and 6xHis-tag (hPD-L1) purified from HEK293 cells was immobilized by amine-coupling using 1:1 (v/v) mixture of 0.1 M *N*-hydroxysuccinimide (NHS) and 0.1 M 3-(*N*,*N*-dimethylamino)propyl-*N*-ethylcarbodimide (EDC) for surface activation. For immobilization, 0.5 µM hPD-L1 in 10 mM sodium acetate buffer (pH 4.5) was injected over the surface of the second flow cell for 600 s at a flow rate of 10 µL/min. Free binding sites on the surface were saturated with 1 M ethanolamine (pH 8) for 420 s at a flow rate of 10 µL/min. Single-cycle kinetics were measured with injections of increasing concentrations of the analyte over both flow cells. The association time was set to 120 s and the dissociation time to 600 s at a flow rate of 30 µL/min.

For the non-optimized and alanine-optimized variants of BN_PDL1_286, a five-fold concentration series ranging from 1.6 nM to 5000 nM was used. For BN_PDL1_482, a five-fold concentration series ranging from 0.32 nM to 1000 nM was used. For both variants of BN_PDL1_1358 and the alanine-optimized variant of BN_PDL1_482, a five-fold concentration series ranging from 0.064 nM to 200 nM was used.

Data were collected at a rate of 10 Hz, and double-referenced by reference flow cell subtraction and blank cycles. Binding parameters were obtained from the kinetic binding measurements using a 1:1 interaction model, using the Biacore^™^ Insight Evaluation Software (version 6.0.7.1750, Cytiva). The fit for the steady state affinities was performed with the following parameters: global KD, global Rmax, global offset. The fit for the 1:1 binding kinetics was performed with the following parameters: global kon, koff, global Rmax, constant drift. For kinetic experiments, an overlay of the experimental and the fitted curves is shown. All relevant data including Rmax, Chi2 and standard errors is presented in Supplementary Data 10. No separate steady-state affinity experiments were performed. All data is based on single-cycle kinetic experiments shown in the manuscript. So termed kinetic affinities were calculated using the 1:1 binding fit, and the respective steady state affinities were determined fitting the same data using the steady state affinity fit.

### Protein thermal stability analyses

Thermal stability of *E.coli*-purified AI-minibinders was analyzed by nanoscale differential scanning fluorimetry using a Prometheus NT.48 (NanoTemper) device. Denaturation and renaturation of proteins was monitored by changes in internal fluorescence at wavelengths of 330 nm and 350 nm. Proteins were diluted to 10 µM in 20 mM HEPES pH 7.4, 150 mM NaCl. The changes in fluorescence were monitored from 20 °C to 90 °C (unfolding) followed by 90 °C to 20 °C (refolding) at a rate of 1.5 °C/min using the PR.ThermControl software (NanoTemper).

### Analytical size exclusion chromatography

Analytical size exclusion chromatography of *E.coli*-purified AI-minibinders was performed on a Superose 6 3.2/300 column (Cytiva) equilibrated in SEC buffer (20 mM HEPES pH 7.4, 150 mM NaCl) and operated at a flow-rate of 0.05 mL/min using the Infinity II HPLC System (Agilent).

### Generation of AI-minibinder CAR constructs

Selected AI-minibinders were ordered as gene fragments (Twist Bioscience) and cloned into our second-generation CAR constructs using BbsI and Esp3I restriction sites. Our murine second-generation CAR comprised a CD8α leader sequence (Mouse NP_001074579.1, residues 1–27), AI-minibinder, HA.11 epitope tag (YPYDVPDYA), CD8α hinge and transmembrane region (Mouse NP_001074579.1, residues 152–220), CD28 intracellular signaling domain (Mouse NP_031668.3, residues 178–218), and CD3ζ chain signaling domain (Mouse NP_001106862.1, residues 62–164). Similarly, the human second-generation CAR consisted of a CD8α leader sequence (Human NP_001139345.1, residues 1−21), AI-minibinder, HA.11 epitope tag (YPYDVPDYA), CD8α hinge and transmembrane region (Human NP_001139345, residues 128–210), CD28 intracellular signaling domain (Human NP_006130.1, residues 180–220), and CD3ζ chain signaling domain (Human NP_000725.1, residues 52–163). Both human and murine CD28 CAR were generated via gene synthesis and cloned into a pMSCV retroviral expression plasmid. CAR expression was coupled with a RQR8 reporter gene via a P2A sequence, as previously described^34^.

### Retroviral vector production

Gamma-retroviral supernatant for the transduction of primary human T cells was prepared as follows. HEK293T cells were transiently transfected with a mastermix containing Opti-MEM (Gibco), the respective AI-minibinder CAR plasmid (5 µg), packaging plasmids Gag-Pol (5 µg), RDF (3.33 µg), and FuGENE transfection reagent (3:1 FuGENE reagent to DNA) and cultured for 48 h at 37 °C in 5% CO_2_. Viral supernatant was filtered through 0.45 µM syringe filter and immediately used for the transduction of human T cells. For the generation of ecotropic gamma-retroviral supernatant for the transduction of primary murine T cells, Platinum-E (Plat-E) retroviral packaging cells were transiently transfected with a mastermix containing Opti-MEM medium, the respective AI-minibinder CAR plasmid (10 µg), and FuGENE transfection reagent (3:1 FuGENE reagent to DNA). Transfected cells were cultured at 37 °C in 5% CO_2_. 48 h post transfection the viral supernatant was harvested, filtered through 0.45 µM syringe filter and immediately used for the transduction of murine T cells.

### Generation of CAR-T cells

To generate human CAR-T cells, peripheral blood mononuclear cells (PBMC) were thawed, resuspended in RPMI complete +100 U/mL rhIL-2 (Peprotech) and activated for 48 h at 37 °C and 5% CO_2_ on CD3 and CD28-coated plates (1 µg/mL, OKT3, Biolegend; 3 µg/mL CD28.2, Biolegend). Next, 24-well non-tissue culture treated plates (Avantor) were pre-coated with 0.5 mL of 10 µg/mL RetroNectin (Takara Bio) at 4 °C overnight. Harvested retroviral supernatant was spun onto RetroNectin-coated wells (at 2000 × *g* for 2 h at 32 °C). Subsequently, activated PBMCs in RMPI complete with 100 U/mL rhIL-2 were added and spinoculated at 800 × *g* for 90 min at 32 °C onto virus-coated plates. PBMCs were subsequently cultured and expanded at a minimum density of 1 × 10^6^ in RPMI complete, supplemented with fresh rhIL-2 100 U/mL every 2 days. Cells were expanded for 7–10 days prior to use in functional assays. To generate murine CAR-T cells, splenocytes were harvested from C57BL/6 mice, mechanically dissociated through a 70-μm filter and red blood cell (RBC) depleted using RBC lysis buffer (Hybri-Max, Sigma-Aldrich). Splenocytes were then resuspended in RMPI complete with 100 U/mL rhIL-2 (PeproTech) +anti-mCD28 (2 µg/mL, 37.51, Biolegend) and seeded onto CD3 (5 µg/mL 17A2, Biolegend) coated plates for 24 h at 37 °C and 5 % CO_2._ Following activation, splenocytes were harvested and transduced via spinoculation (800 × *g* for 90 min at 32 °C) onto RetroNectin 10 µg/mL coated non-tissue culture treated plates covered with retroviral supernatant (2000 × *g* for 2 h at 32 °C). Splenocytes were subsequently expanded at a minimum density of 1 × 10^6^ in RPMI complete, supplemented with fresh 100 U/mL rhIL-2 for the first 48 h. Afterwards, rhIL-2 was replaced with rmIL-7 (10 ng/mL) (PeproTech) and rmIL-15 (10 ng/mL) (PeproTech). Cells were expanded for 7–10 days prior to use in functional assays.

### Assessment of CAR-T cell transduction efficacy

CAR surface expression on primary T cells was analyzed via flow cytometry. Transduced cells were washed with 4 °C PBS, then blocked with Fc receptor blocking solution (TruStain FcX^TM^, Biolegend) and stained with Live/Dead^TM^ Fixable Near-IR Dead Cell Stain Kit (Thermo Fisher) according to the manufacturer’s instructions. Cells were subsequently stained with fluorophore-coupled anti-hCD34 and anti-HA.11 antibodies to detect RQR8 and CAR expression on the surface of T cells, respectively. For the full surface staining, fluorophore-coupled antibodies against the following antigens were resuspended in FACS buffer (PBS, 2% (v/v) FBS, 2 mM EDTA): CD45 (clone HI30, BD Biosciences), CD3 (clone UCHT1, Biolegend), CD8 (clone RPA-T8, BD Biosciences), and CD4 (clone SK3, Biolegend). Samples were acquired using the 4-Laser Cytek Aurora Spectral Flow Cytometer or 5-Laser Sony ID7000 Spectral Cell Analyzer, and analyzed using FlowJo software.

### CAR-T cell activation assay

CAR-T cells were plated with indicated target cells at an effector to target ratio of 1:1 in 96-well U bottom plates in RPMI complete media for 5 h, to determine intracellular cytokine production, or for 24 h to measure the expression of surface CD25, at 37 °C and 5% CO_2_. To assess IFN-γ production, the protein transport inhibitors Monensin (GolgiStop^TM^, BD Biosciences) and Brefeldin (GolgiPlug^TM^, BD Biosciences) were added at 1:1000. First, a surface staining to identify CAR-T cells was performed as described above, followed by fixation and permeabilization for intracellular staining of IFN-γ (clones 4S.B3 or XMG1.2, Biolegend) and TNF-α (clones Mab11 or MP6-XT22, Biolegend) according to the eBioscience^TM^ Foxp3/Transcription Factor Stain Buffer Set (Thermo Fisher). To assess induction of CD25 on CAR-T cells, co-cultures were harvested and surface staining was performed as described above including an anti-CD25 (clone BC96, Biolegend) antibody. Samples were acquired with a 4-LaserCytek Aurora Spectral Flow Cytometer and analyzed using FlowJo software.

### CAR-T cell killing assay

Killing capacities of human CAR-T cells were evaluated using the label-free impedance-based xCELLigence RTCA SP platform (Agilent Technologies) in concordance with the protocol from Kanemaru et al.^51^. 50,000 target cells were seeded in RPMI complete per well in E-plates and incubated for 24 h before adding CD34-sorted CAR-T cells in 1:1 effector-to-target ratios. Cell growth was monitored by impedance-measurements in 15-min intervals for further 24 h and displayed as cell index values. Measurements were normalized to the time point of CAR-T cell addition and the respective condition of target cells alone to plot the cytolysis index. By computing the area under the curve (AUC) for every sample and subtracting the respective control with CD276 KO cells, biological triplicates were quantified for statistical analyses.

### Recombinant Fc-fusion protein expression and purification

The plasmid pD649-HAsp-CD276-Fc(DAPA)-AviTag-6xHis^41^ (Addgene, #156861) was used for expression of the extracellular domain of CD276 human Ig-Fc fusion protein. CD276 Fc-constructs were produced and purified as previously described^52^. Briefly, 30 µg eukaryotic expression vector for CD276 Fc-constructs were transiently transfected into HEK293F cells and recombinant CD276 Fc-fusion proteins were purified under sterile conditions from culture supernatants using Protein G beads (GE Healthcare). Glycine solution (0.1 M, pH 3.0) was used for elution from Protein G beads, and the solution was buffered to pH 7.4 using Tris buffer (1 M, pH 8). Concentrations were determined by ELISA. pD649-HAsp-CD276-Fc(DAPA)-AviTag-6xHis was a gift from Chris Garcia (Addgene plasmid #156861; http://n2t.net/addgene:156861; RRID:Addgene_156861)^41^. Commercially available Fc-fusion proteins, or provided by the Core Facility Nanobodies (CFN, UKB, Bonn), used in this study are listed in Supplementary Data 9.

### Re-scoring

To score the designed structures in complex with their target we used AlphaFold 2 (AF2) initial guess^14^, Chai-1^26^ and Boltz-1^27^. All models were run on a single NVIDIA L40S GPU. AF2 initial guess was used through the scripts provided by Bennet et al.^14^ and run with default parameters on all designs. pAE interaction scores were calculated after the initial design and filtering over the number of alpha-helices. For further rescoring, we assessed the results that were included in the AI-minibinder design libraries. Chai-1 (v.0.5.2) was used via the Python interface provided by the authors^26^. The model was run with sequence information and ESM2 embeddings as input. Sequences were obtained from the PDB files that were outputted from the AF2 initial guess pipeline. No multiple sequence alignments (MSAs) were provided to the model. For scoring, we decided on using the standard settings: 3 truck recycles, 200 diffusion time steps. Boltz-1 (v.0.4.0) was run with recycling_steps = 10, diffusion_samples = 5 as input parameters. MSAs were generated to provide evolutionary information for subsequent protein structure prediction. Protein sequences were compiled into a single FASTA format file. Each sequence contained a unique identifier in the header line followed by the amino acid sequence. Two complementary sequence databases were employed for comprehensive homology searching: uniref30_2302_db and colabfold_envdb_202108_db. MSAs were generated using the MMseqs2 suite with GPU acceleration^28^ through ColabFold^53^. The specific procedure was as follows: MMseqs2 GPU servers were initialized for both the UniRef30 and environmental databases with the following parameters: Maximum sequences per query: 10,000; Database load mode: 0; Prefilter mode: 1. Sequence similarity searches were performed using the ColabFold search tool with these key parameters: –gpu 1; –gpu-server 1; -s 8.5; –db-load-mode 0; –pairing_strategy 1; –use-env 1; –use-templates 0.

For each query sequence in the input FASTA file, homologous sequences were identified, aligned, and compiled into separate MSA files in A3M format. The quality of each MSA was assessed by quantifying the number of sequences captured, providing an indication of the depth of evolutionary information available for each query protein. The entire MSA generation process was executed in an automated fashion using a custom pipeline implemented in Bash. These MSAs were subsequently utilized as input for protein structure prediction via Boltz-1.

All used models and packages are available on GitHub under the following repositories: AF2 initial guess (https://github.com/nrbennet/dl_binder_design), Chai-1 (https://github.com/chaidiscovery/chai-lab), Boltz-1 (https://github.com/jwohlwend/boltz), ColabFold (https://github.com/sokrypton/ColabFold). Databases for MSA generation were obtained from https://colabfold.mmseqs.com.

### Statistical analysis

Statistical analyses were performed using GraphPad Prism (v10) and R. The statistical tests applied, including whether they were two-sided, are specified in the figure legends. For comparisons involving multiple groups, ANOVA was followed by pairwise comparisons with correction for multiple testing. In this case, significant differences are shown as indicated, whereas non-significant comparisons are not displayed for clarity.

### Sample size estimation

For pooled minibinder screening, library and design numbers were chosen based on prior large-scale AI-based protein binder screens and reported success rates, and adjusted based on success rate obtained throughout our study. Unless otherwise indicated, experiments were performed with at least three independent biological replicates, which were considered sufficient to assess reproducibility and estimate biological variability across independent experiments.

### Use of AI tools

ChatGPT (OpenAI, GPT-5.3) was used to support text editing and code development; all content was critically reviewed and verified by the authors.

## Supplementary information

Supplementary Information

Description of Additional Supplementary Data

Supplementary Data 1

Supplementary Data 2

Supplementary Data 3

Supplementary Data 4

Supplementary Data 5

Supplementary Data 6

Supplementary Data 7

Supplementary Data 8

Supplementary Data 9

Supplementary Data 10

Supplementary Data 11

Supplementary Data 12

Transparent Peer Review file

## Source data

Source Data

## Data Availability

Source are provided with this paper. Protein designs generated in this study have been deposited in the Zenodo database under 10.5281/zenodo.19512851, and 10.5281/zenodo.19167586. The NGS data have been deposited in the NCBI database under accession code PRJNA1494948 with raw sequencing files and processed files stating the read counts per binder. Other data generated in this study are provided in the Supplementary Data and Source Data file. All additional source data is provided as ExtendedSourceData.zip via Zenodo: 10.5281/zenodo.21399936. Applied accession codes: 4ZQK, 4GOS, CD276 AF model[https://alphafold.ebi.ac.uk/entry/AF-Q5ZPR3-F1], Q9NZQ7-1, Q5ZPR3-1, Q7Z7D3-1, NP_001074579.1, NP_031668.3, NP_001106862.1, NP_001139345.1, NP_006130.1, NP_000725.1 Source data are provided with this paper.

## Code availability

The code used to develop the design algorithms and perform analyses in this study is publicly available and has been deposited on GitHub at https://github.com/HoelzelLab/IEO_AI_Binder_cancer_surface_2025, under MIT license. The specific version of the code associated with this publication is archived in Zenodo and is accessible via doi.org/10.5281/zenodo.21395089. The code used for the HM9 minibinder variant generation has been deposited in Zenodo and is accessible via doi.org/10.5281/zenodo.19167586. To use the code, users must install dependencies separately. The code is designed with the assumption that these dependencies are installed in distinct Conda environments. When utilizing these tools, users have to cite each one individually and review and adhere to their respective licenses. RFdiffusion: https://github.com/RosettaCommons/RFdiffusion; Biopython: https://biopython.org; ProteinMPNN/AF2-initial-guess: https://github.com/nrbennet/dl_binder_design; Chai-1: https://github.com/chaidiscovery/chai-lab; Boltz-1: https://github.com/jwohlwend/boltz; ColabFold: https://github.com/sokrypton/ColabFold.
