# Supplementary material for: AI-enabled discovery and biochemical optimization of minibinders targeting cancer cell-surface proteins
Source: Nat Commun. 2026 Aug 20;17:8736. doi: 10.1038/s41467-026-76760-5 (PMC13494018; doi:10.1038/s41467-026-76760-5)
Supplement: Supplementary file 1 [file 41467_2026_76760_MOESM15_ESM.pdf]

nature portfolio

Peer Review File

AI-enabled discovery and biochemical optimization of minibinders targeting cancer cell-surface proteins

Corresponding Author: Professor Michael Hölzel

This file contains all reviewer reports in order by version, followed by all author rebuttals in order by version.

Version 0:

Reviewer comments:

Reviewer #1

(Remarks to the Author)
This study designed binders that bind to cancer cell surface proteins using state-of-the-art de novo design tools. By utilizing limited computational resources, the authors provided a workflow that allows a wide range of non-expert researchers to attempt AI-driven protein design. They performed de novo design for three antigens and conducted experimental screening, discovering that the success rate varied depending on the antigen. The obtained binders were then assembled and applied to the detection of cells displaying target proteins.

This is impressive work and will likely be beneficial for wet-lab researchers. However, if the paper's stance is to propose a binder design method, the basic AI-design workflow doesn't appear to be much different from previous works(e.g., ref. 13). It would be better to highlight more clearly what differentiates this method from existing approaches.

(page 3, line 16)
What does "scalable pipeline" refer to? Does it mean applicable to various target molecules?

(page 3, line 21)
What changes when GPU performance is limited? For example, does it affect the number of sequences that can be proposed, or the length of the amino acid sequence?

(page 3, Results, first section (Pipeline for AI-binder design and functional screening))
(i) The objective of this study is to develop an "accessible and scalable pipeline to design small AI-binders." Within the workflow developed by the authors, the steps of backbone design (RF diffusion), side-chain design (proteinMPNN), and filtering by pAE are the same as in previously reported studies (ref6, 13). Therefore, the novelty in the design part appears to be in the Filter scripts (size, #helices, #sheets). If so, could you provide data to show that the newly introduced Filter scripts have advanced de novo design?

(ii) This study will likely answer the question of how non-expert researchers in informatics science can obtain AI-designed binders. From this point of view, the design of length and the selection of Hotspots should be described in a more detailed manner.
- What variations were used for designing different lengths?
- How were Hotspots determined? How were combinations of Hotspots considered?
- How many amino acid sequences were generated by pMPNN for one poly-glycine scaffold proposed by RF diffusion?

(page 5, line 25-27)
Figure 2f only shows the results for PD-L1. Are the data for CD276 and VTCN1 available elsewhere?
Relatedly, is it correct that DNA from the 2.49% of cells within the square in Figure 2f was extracted and subjected to the NGS analysis? If so, please provide a figure like Figure 2f for Figures 2h and 2i and indicate from which gate the cells were recovered.

(Figure 2j)
Seems to be a small number of plots, does it contain plots for 1000 sequences? Are they filtered in some way?

(Figure 2k)
For example, PD-L1 has 40000 designs, but the histogram shows n=500. How should this be interpreted?

(page6, line10-11)
What determined “the most effective hotspot”?

(page6, line28-29)
What are “non-conservative interface mutations”? Why did the authors choose one or two residues among the multiple residues located at the interface between AI-binders and PD-L1? How did the authors choose the four binders in Figure 3c?

(page7, line9-10)
How did the authors optimize the Alanine patches? Since generative AI often suggests biased amino acid sequences, it would be beneficial to describe such findings for unlocking the broader potential of AI-based protein design.

(page7, line11-18)
Supplementary Figure 3 shows that the yield is improved by optimizing the Alanine patch. And for BN_1358 and BN_1358_AlaOpti, the change in fluorescence signal intensity due to DSF results is large, and the physical properties appear to be better for BN_1358_AlaOpti (BN_1358 may be a naturally denatured protein). Do the authors not think that this is the result of optimization of Alanine patches?

(page10, line3-4, Figure 5a)
The rescoring process seems a bit complicated. Boltz works with only amino acid sequences as input, but why do the authors have to go through Chai-1 ESM to enter Boltz-1? Also, why do the authors have to go back to Chai-1 MSA after entering Boltz?

(page10, line24-28)
The authors state that Chai-1 is superior to pAE by comparing Group A, B, and C, respectively, but if they focus on each model and evaluate its performance, shouldn't they compare A+B and B+C?

(page 11, line 15-20)
What would happen if strong-binding Binders (MiB, HM9) were added to this plot? If the positive control results are the same for AF3 and Chai-1 ESM, then it can be said that common results are obtained at least for strongly binding Binders. If so, couldn't ΔipTM be used as an indicator other than pAE for protein design?
Also, Figure 6h and 6i include sequences that showed no difference in binding affinity in experiments. What would happen if the graph was limited only to sequences where binding affinity clearly decreased due to mutation? This might make Chai-1 appear to represent the experimental results better than AF3.

(page 11, line 23-25)
What are the advantages of detection reagents using AI-binders compared to those using conventional antibodies?

(page 12, line 22-26)
Among the anti-CD257 binders, 616_606 seems to be the most promising, so why was a quattrobinder not created?

Minor point

(Supplementary Figure 2a)
What are FSC-A, SSC-A, and FSC-H?

(Method)
Several subscripts that should be present are shown in normal size.
Example: page 18, line 10: MgCl2

(Remarks on code availability)

Reviewer #2

(Remarks to the Author)
This is a unique and compelling manuscript that presents a workflow bridging artificial intelligence (AI)-based protein design and experimental validation across both cell–molecule and molecule–molecule interactions, with a particular focus on immunoglobulin superfamily (IgSF) cell surface proteins involved in immune checkpoint inhibition (ICI). AI-driven protein design is a rapidly evolving and inherently challenging field with significant potential for novel therapeutic applications. From the perspective of a cancer biologist and IgSF researcher, I would like to raise the following points that should be clarified or expanded upon to strengthen the manuscript.

1. N-Glycosylation and IgSF Protein Diversity
It is well established that more than half of IgSF proteins exhibit structural and functional diversity due to N-glycosylation, particularly within their variable Ig-like loops. In fact, a potential N-glycosylation motif (N-X-S/T) is present within the Ig-like

V-type loop of PD-L1, conserved across human and mouse orthologs (Fig. 3g). In this study, AI-designed binder proteins were expressed in E. coli for SPR analysis, in HEK293 cells for flow cytometry, and in T cells for CAR-T applications—systems that differ substantially in their capacity for post-translational modifications, especially glycosylation. These differences may influence binding activity and could account for variability observed across experimental systems. The authors are strongly encouraged to examine and discuss potential differences in N-glycosylation among representative AI binders, and to address how such modifications might affect molecular interactions involving the IgSF proteins studied.

2. SPR Methodology and Data Presentation
The authors performed surface plasmon resonance (SPR) analysis using Biacore™, providing robust and quantitative assessments of protein–protein interactions. The data presented in Figures 4h–j and Supplementary Figures S3a–c are convincing and support the study's conclusions. However, given the importance of using a series of five concentrations of AI binders for interpreting kinetic parameters, the exact concentration ranges should be clearly stated not only in the Materials and Methods section but also in the main figures or their legends to ensure clarity and reproducibility. Additionally, it would be important to explicitly mention that the protein fragments used for SPR analysis were produced in E. coli, which lacks N-glycosylation machinery. This contextual detail is essential given the topic of this study.

3. Target-Dependent Efficacy of RFdiffusion
The authors demonstrate that the RFdiffusion model generated numerous candidate AI binders for PD-L1, but relatively few for CD276 and VTCN1. Based on these observations, they conclude in the Abstract that RFdiffusion exhibits target-dependent efficacy in generating high-quality designs. While I agree with this data-driven and neutral conclusion, the manuscript would benefit from further mechanistic interpretation. PD-L1 and PD-1 form a well-characterized, high-affinity interaction that is central to ICI therapy, whereas definitive binding partners for CD276 and VTCN1 remain controversial. Several have been proposed—e.g., BTLA and TREM-like receptors for CD276, and TLT-2 for VTCN1—but their physiological relevance is less well established. Additionally, ALPHA (Amplified Luminescent Proximity Homogeneous Assay) and other data have shown that the PD-L1–PD-1 and CTLA4–CD80/CD86 interactions are substantially stronger than many other IgSF interactions, although ALPHA is less quantitative than SPR (see: Verschueren et al., Cell 2020, 182(2):329–344.e19; Wojtowicz et al., Cell 2020, 182(4):1027–1043.e17). In light of these differences, I strongly encourage the authors to provide a hypothesis or commentary on why RFdiffusion may exhibit higher efficacy for PD-L1 than for CD276 or VTCN1. Such a discussion would substantially enrich the mechanistic insight and broaden the impact of the paper.

(Remarks on code availability)
I have reviewed the code, but the contents and total volume are essentially same as the manuscript that was submitted to Nature Communications in this time, thank you.

Reviewer #3

(Remarks to the Author)
This study incorporates de novo AI-designed protein binders into second-generation CAR-T cells targeting PD-L1 and CD276. The authors show antigen-specific T cell activation in vitro, including against endogenously expressed targets. The work supports using AI binders in CAR design, but several key gaps remain.

Major Concerns
1. The most critical missing piece is the absence of in vivo validation. The paper includes solid in vitro cytokine data but never tests whether these CAR-T cells actually work in vivo. There's no evidence for expansion, persistence, trafficking, or tumor control. A basic xenograft model with PD-L1$^+$ or CD276$^+$ tumors in NSG mice would have been enough to test specificity and therapeutic potential. Right now, there's no way to know whether these CARs can eliminate tumors or avoid off-target toxicity.

2. Not all CAR constructs expressed well on the surface. MiB and HM9 in particular showed poor trafficking, probably due to binder misfolding or structural instability. This is a problem that needs deeper analysis. The authors should compare the primary and predicted tertiary structures of well- and poorly-expressed binders, and consider redesigning the hinge or linker. Quantitative surface expression should be shown. If binders fail to fold properly, they shouldn't be used in CAR format.

3. All functional validation is based on intracellular cytokine staining (IFN-γ, TNF-α). That's a good start, but it's not enough. There is no direct quantification of killing, so we don't know if these CARs can actually kill tumor cells. That's a basic requirement.

4. The data show that only high-affinity binders triggered CAR activation against endogenous CD276. Lower-affinity designs only worked on overexpressed antigen. This reflects a classic problem in CAR design that affinity must be high enough for sensitivity, but not so high that it drives off-tumor toxicity. Authors should further discuss the clinical implications of this finding, and how further to optimize their model to ensure inclusion of high-affinity binders.

5. Both PD-L1 and CD276 are expressed on some normal tissues. This raises the possibility of on-target/off-tumor toxicity. The authors should acknowledge this and propose next steps.

6. Finally, PD-L1 CARs could be functionally impacted by PD-1/PD-L1 signaling. The authors should briefly mention whether PD-1 was expressed in their model system or whether reverse signaling was considered.

Minor Concerns
• Provide amino acid/nucleotide sequences of binders in Supplementary Tables.
• Clarify HA-tag language ("CAR surface expression via HA-tag").
• Ensure consistent terminology ("CAR T cells" vs "CAR-T cells") and specify primary cells when applicable.
• State what error bars represent (likely mean ± SEM) and what the two-way ANOVA compared.
• State co-culture duration and E:T ratio for ICS and activation assays in the figure legends.
• Clarify murine vs human CAR construct differences (species-specific domains, same binder, RQR8 epitope detection in both).
• Note rationale of costimulatory domain choice (CD28ζ).
• Briefly cite other non-antibody binders used in CAR T cells (nanobodies, DARPins) to place work in broader context, and note how AI-designed binders compare (smaller, epitope-specific, but require empirical validation for CAR expression/affinity).

(Remarks on code availability)

Version 1:

Reviewer comments:

Reviewer #1

(Remarks to the Author)
The authors addressed all my comments. They provided new analyses and significant textual revisions to satisfy my concerns.

(Remarks on code availability)

Reviewer #2

(Remarks to the Author)
The authors have revised the manuscript in accordance with the reviewer's suggestions and comments. Reviewer 2 has mainly focused on evaluating the authors' responses to the three major comments previously raised and is now satisfied with the appropriateness and quality of the revisions.
The prediction that potential N-glycosylation sites are located on the side opposite to the interacting interface between PD-1 and PD-L1, as well as other IgSF proteins, is particularly interesting (Comment 1). Reviewer 2 was also impressed by the observation that asparagine (N) residues are underrepresented, whereas the N-X-S/T glycosylation motif itself is not (Comment 2). Finally, Reviewer 2 appreciates the authors for adding further discussion regarding the possible biological significance of the efficiency of RFdiffusion-based prediction (Comment 3; p. 26, line 612; references 37 and 38).
At this stage, Reviewer 2 has no further comments on this potentially interesting and well-organized manuscript.

(Remarks on code availability)
I have checked the preprint and recognized that the content is essentially the same as the original manuscript.

Reviewer #3

(Remarks to the Author)
The authors have substantially revised their manuscript in response to three reviewers comments. They added new data on CAR surface expression and biochemical optimization, clarified methodological details and corrected errors. However, several replies remain superficial or speculative, and the study still suffers from several shortcomings
1-The authors now say they built an accessible binder-design pipeline, but the only tweak they really describe is a filter that dumps designs with fewer than three helices. They don't provide any data that this filter actually improves success. all it does is save computing time. Without evidence that their workflow produces better binders than existing methods, it's hard to see the value of this work.
2-They limit all their scaffolds to 70–88 amino acids. That might be convenient for ordering DNA oligos, but there is no justification for why other lengths weren't explored. As someone who has seen CAR surface expression hinge on binder size and stability, I find the narrow length window troubling.
3-When the first round of design didn't yield many CD276 or VTCN1 binders, the authors simply picked the best hotspot as the one that produced more low-pAE designs. There is no structural rationale for why these hotspots should be good or bad. Without understanding why a particular interface works, it's hard to believe the approach will generalize. In the second round of design they got additional hits for CD276 and few candidate VTCN1 minibinders, and when they tested those individually the binding patterns were much weaker than for PD-L1. That's a long way from proving the method works broadly.
4-The discussion around alanine patch optimization is speculative. in the rebuttal the authors admit they can only guess that their best binder forms dimers to explain its melting behaviour. Likewise, the isoelectric-point optimisation that improves HM9-based CARs is shown only for one scaffold. They present this as a general solution, but there is no data that adjusting pI will help other binders, and the basic variants still showed off-target killing.

5-Despite adding lots of in vitro data, the authors still provide no animal data. They acknowledge this in the discussion and lean on another group’s mouse study. For a platform that is supposed to point toward therapeutic CAR-T cells, the absence of even a simple xenograft experiment is a major gap.
6-All of the binders were expressed in E. coli, which has no N-glycosylation machinery. The authors note that none of the binders contains a canonical N-glycosylation motif and that asparagine is under-represented, but they never actually test whether glycosylation of the target affects binding or whether the binders fold and function properly in mammalian cells. This is a huge gap if you are aiming for therapeutic use.

(Remarks on code availability)

Version 2:

Reviewer comments:

Reviewer #3

(Remarks to the Author)
I appreciate the authors thoughtful responses and the substantial additional work added to the revised manuscript. I also appreciate the clearer framing of the three helix filter as a workflow efficiency and prioritization step, not as evidence that the designs are intrinsically superior. I still believe that in vivo validation would be important, especially before drawing broader therapeutic conclusions from the CAR T data. However, I do not want to continue holding the manuscript on this point if the editors are comfortable with the current scope and the stated limitations. Overall, I am satisfied that the major concerns have been addressed and I have no further substantive comments.

(Remarks on code availability)

Open Access This Peer Review File is licensed under a Creative Commons Attribution 4.0 International License, which permits use, sharing, adaptation, distribution and reproduction in any medium or format, as long as you give appropriate credit to the original author(s) and the source, provide a link to the Creative Commons license, and indicate if changes were made.
In cases where reviewers are anonymous, credit should be given to 'Anonymous Referee' and the source.
The images or other third party material in this Peer Review File are included in the article’s Creative Commons license, unless indicated otherwise in a credit line to the material. If material is not included in the article’s Creative Commons license and your intended use is not permitted by statutory regulation or exceeds the permitted use, you will need to obtain permission directly from the copyright holder.
To view a copy of this license, visit https://creativecommons.org/licenses/by/4.0/

Point-to-point reply regarding revised manuscript NCOMMS-25-38814

Title of revised manuscript:

AI-enabled discovery and biochemical optimization of minibinders targeting cancer cell-surface proteins

Previous title: Development of AI-designed protein binders for detection and targeting of cancer cell surface proteins

General remarks to all reviewers:

We thank the reviewers for their constructive and insightful feedback, which has helped to substantially improve our manuscript. A key point raised, in particular by Reviewers 2 and 3, concerns how de novo designed proteins behave in cellular contexts, exemplified here by the observation that some minibinder-based CAR constructs showed limited surface expression. We fully agree that this represents a fundamental aspect that must be addressed in greater depth to advance AI-designed minibinders toward functional applications such as CAR-T cell therapies.

In response, we have undertaken a comprehensive and systematic analysis of the determinants governing CAR surface expression, generating substantial new experimental data (new main Figures 7 and 8, and Supplementary Figure 10). Specifically, we implemented a computational genetic algorithm–based optimization strategy in which the binding interface was preserved while non-interface residues were iteratively diversified, enabling the screening of thousands of variants across a broad physicochemical space. These studies identify the isoelectric point of minibinders as a key determinant of surface accessibility and demonstrate that biochemical properties beyond the binding interface can be rationally optimized to improve trafficking, activity, and target-selective tumor cell killing. Importantly, this establishes that suboptimal performance of individual constructs is not an inherent limitation of the scaffold but can be overcome through targeted design strategies.

We believe that these new findings significantly strengthen the novelty and timeliness of our work, as they provide a conceptual framework for optimizing de novo designed minibinders in complex cellular systems. In light of these substantial additions, we have also restructured and refined the focus of the manuscript to better reflect the expanded scope and impact of the study.

Our revised manuscript is therefore now entitled: "AI-enabled discovery and biochemical optimization of minibinders targeting cancer cell-surface proteins"

For your convenience, we list changes and corrections:

In the marked-up manuscript, revised text passages addressing referee questions are highlighted in yellow. Text passages describing completely new data are highlighted in cyan.

Main figures
-Figure 4, revised figure 5 from previous version
-Figure 5, revised figure 7 from previous version
-Figure 6, revised figure 4 from previous version

-Figure 7 and figure 8, new data, role of minibinder pI for surface expression and function

Supplementary figures
-Supplementary figure 1, revised supplementary figure 1 from previous version with new panels a and b
-Supplementary figure 3, revised supplementary figure 7 from previous version
-Supplementary figure 4, supplementary figure 3 from previous version
-Supplementary figure 5, revised supplementary figure 4 from previous version
-Supplementary figure 6, contains revised panels from main figure 6 of previous version
-Supplementary figure 7, revised data from previous supplementary figure 8
-Supplementary figure 8, previous supplementary figure 5
- Supplementary figure 9, revised supplementary figure 6 from previous version
-Supplementary figure 10, new data related to new main figures 7 and 8

Supplementary tables:
-Supplementary table 10 (new), for convenience, list all amino acid sequences, reverse translated cDNA sequences and order DNA oligo sequences as well as original pdb or cif file names of minibinders studied in greater detail in our work. In addition asparagines (N) and, if present, canonical N-X-S/T glycosylation sites are highlighted.

-In the previous version, we noted that in Supplementary Table 7, the sequence and AF3 ipTM scores of the PD-L1 minibinder BN_401 were erroneously duplicated from BN_1028. This has been corrected in the revised version, including in the corresponding plots in Supplementary Figure 6d,e.

-We corrected the labeling of a cell line (A375 melanoma instead of SK-Mel-28). All cell lines used in this study were re-authenticated by short tandem repeat (STR) profiling.

-For consistency with study by Xia et al. Nat. Biomed. Eng 8, 1634–1650 (2024), we adjusted the name of CD276 minibinder MiB to mb.

Reviewers' comments:

Reviewer #1 (Remarks to the Author):

This study designed binders that bind to cancer cell surface proteins using state-of-the-art de novo design tools. By utilizing limited computational resources, the authors provided a workflow that allows a wide range of non-expert researchers to attempt AI-driven protein design. They performed de novo design for three antigens and conducted experimental screening, discovering that the success rate varied depending on the antigen. The obtained binders were then assembled and applied to the detection of cells displaying target proteins.

This is impressive work and will likely be beneficial for wet-lab researchers. However, if the paper's stance is to propose a binder design method, the basic AI-design workflow doesn't appear to be much different from previous works(e.g., ref. 13). It would be better to highlight more clearly what differentiates this method from existing approaches.

We thank the reviewer for the very positive evaluation of our work.

The central focus of our study is the practical application of protein design methods and the development of AI-designed minibinders for downstream use. This focus is further underscored by the new data on CAR surface expression and functionality (Figs. 7–8 and Supplementary Fig. 10).

In line with this application-oriented perspective, we introduced additional secondary-structure-based filtering to remove, at an early stage, designs with only limited prospects for experimental success, such as unstable double-helical folds. Our experimental results also enabled us to benchmark and validate several models for the in silico selection of binders. We therefore believe that the importance of our study lies in the unbiased and independent experimental evaluation of the workflow, together with the establishment of a comprehensive experimental strategy that can be readily adopted by the community. Moreover, the new data on the biochemical determinants of CAR surface expression and functionality substantially strengthen the novelty and translational relevance of our work.

(page 3, line 16)
What does "scalable pipeline" refer to? Does it mean applicable to various target molecules?

Our use of the term scalable refers primarily to wet-lab validation. Although the computational pipeline is likewise scalable, our main point was that mammalian surface display enables parallel experimental screening of thousands of binders. In our view, the key bottleneck in the field is often not computational design, but experimental validation. This platform therefore offers an efficient strategy to identify candidates with favorable properties for mammalian expression and downstream applications, including chimeric antigen receptors in human T cells.

We now clarified this within the next (now p3, line 78): "Here, we developed an accessible pipeline for designing small artificial protein binders (AI minibinders) against three cancer surface targets of the B7-H family and established a scalable experimental workflow for their validation.", and in the abstract we say "…scalable experimental workflow…".

(page 3, line 21)
What changes when GPU performance is limited? For example, does it affect the number of sequences that can be proposed, or the length of the amino acid sequence?

Limited GPU performance does not modify the produced results but the speed at which the designs are generated and filtered in silico. Users with limited GPU performance might be limited in the number of designs that they can generate within a predefined time frame. The most limiting factor might be the GPU memory. If a larger target chain is provided to the pipeline the memory consumption and runtime increases. Therefore, it is advisable to input a truncated target if the target structure allows for clean truncation (e.g. splitting by structurally distinct domains). This strategy was also proposed by Watson et al.

(page 3, Results, first section (Pipeline for AI-binder design and functional screening))
(i) The objective of this study is to develop an "accessible and scalable pipeline to design small AI-binders." Within the workflow developed by the authors, the steps of backbone design (RF diffusion), side-chain design (proteinMPNN), and filtering by pAE are the same as in previously reported studies (ref6, 13). Therefore, the novelty in the design part appears to be in the Filter scripts (size, #helices, #sheets). If so, could you provide data to show that the newly introduced Filter scripts have advanced de novo design?

The newly introduced filter script does not improve or extend the de novo design algorithm itself. Backbone generation by RFdiffusion, sequence design by ProteinMPNN, and AlphaFold-based scoring remain conceptually unchanged. Instead, our filtering step was introduced to improve the efficiency of the pipeline by excluding, at an early stage, designs with only two helical segments, which our initial test expression data suggested were often unstable and therefore less likely to succeed experimentally. This preselection enriches for predicted three-helical folds before ProteinMPNN design and AlphaFold pAE scoring, both of which are computationally time-consuming steps. Thus, the main contribution of this filtering step is not the generation of more advanced de novo designs, but a more efficient prioritization of candidates with a higher predicted probability of stability and downstream success.
We clarify this in the text (now p4 line 116): "*Only the designs that passed this selection step were fed into the subsequent stages of our pipeline, thereby saving time in amino acid sequence design with ProteinMPNN and pAE interaction scoring with AF2. We were confident that the resulting designs should have a better chance to work in real-world applications.*"

(ii) This study will likely answer the question of how non-expert researchers in informatics science can obtain AI-designed binders. From this point of view, the design of length and the selection of Hotspots should be described in a more detailed manner.

- What variations were used for designing different lengths?
Please see answer below.

- How were Hotspots determined? How were combinations of Hotspots considered?

As described by Watson et al., RFdiffusion is particularly effective when binder design can be guided toward well-defined interaction hotspots, such as patches of hydrophobic amino acids that provide favorable interfaces for stable molecular recognition.

For PD-L1 minibinders, we clarified hotspot selection as follows (now p6 line 151): "PD-L1 was included as a benchmarking target, since Watson et al. previously designed AI-minibinders against PD-L1 using RFdiffusion7, enabling us to select a patch of hydrophobic amino acids at the binding interface of their validated PD-L1 minibinders as hotspot for our design process. Furthermore, this hotspot was located opposite to an evolutionary glycosylation site (N-M-T, position 35 in PD-L1 Q9NZQ7-1) avoiding potential interferences by glycosylation."

For CD276 and VTCN1 we clarify (p7 line 158): *"In contrast, the design of candidate binders against CD276 and VTCN1 was less efficient, despite testing multiple hotspots (Supplementary Table 1). These hotspots were defined by distinct combinations of hydrophobic amino acids, which may form favorable interaction patches that usually increase the likelihood of successful minibinder design by RFdiffusion, and were intentionally positioned opposite glycosylation sites to minimize potential interference."*

- How many amino acid sequences were generated by pMPNN for one poly-glycine scaffold proposed by RF diffusion?

We now clarified this at the beginning of the results section (now p4 line 98): "Following the methodology developed by Watson et al.7, we generated poly-glycine scaffolds using RFdiffusion (length between 70 and 88 amino acids), then applied ProteinMPNN (PMPNN)13,14 to design amino-acid side chains (two variants per scaffold), and finally evaluated each sequence with the AlphaFold2 (AF2) initial-guess algorithm[14] (Fig. 1a)."

(page 5, line 25-27)
Figure 2f only shows the results for PD-L1. Are the data for CD276 and VTCN1 available elsewhere?

The FACS plots with gates are now shown in Supplementary Figure 1a,b. Figure panel call out now p7. Line 169: "*We detected a 2.4% positive PD-L1-binding population, compared with below 1% for both CD276 and VTCN1 (Fig. 2f, Supplementary Fig. 1a, b).*."

Relatedly, is it correct that DNA from the 2.49% of cells within the square in Figure 2f was extracted and subjected to the NGS analysis?

Yes, this is correct.

If so, please provide a figure like Figure 2f for Figures 2h and 2i and indicate from which gate the cells were recovered.

The gates are shown in Supplementary Figure 1a,b. (see above).

(Figure 2j)
Seems to be a small number of plots, does it contain plots for 1000 sequences? Are they filtered in some way?
Sequences are not filtered. However, all sequences without enrichment by the panning overlap. This leads to the impression of less points, although all 1000 are shown. A conclusion is that the phage display is more stringent than the mammalian cell surface display.

(Figure 2k)
For example, PD-L1 has 40000 designs, but the histogram shows n=500. How should this be interpreted?

The histograms show only the selected binders (n=500, top candidates) included in the pool from 40,000 designs in total.

To clarify that, we adjusted the legend of Figure 2: *"**k** Histogram showing the distribution of pAE interaction scores for designs selected into the improved pools (n = 500 or 750) from the total initial design sets (n = 40,000, 90,000, or 75,000)".*

(page6, line10-11)
What determined "the most effective hotspot"?

Most effective hotspots were defined by the number of successful results with same settings but different hotspot positions.
We now clarify this in the text (p7 line 183): "*For the second round of computational design, we selected those hotspots for CD276 and VTCN1 identified in the first screening pool (Supplementary Fig. 1e, f; Supplementary Tables 4–6) that yielded the highest number of designs with pAE scores below 5*"

(page6, line28-29)
What are "non-conservative interface mutations"? Why did the authors choose one or two residues among the multiple residues located at the interface between AI-binders and PD-L1? How did the authors choose the four binders in Figure 3c?

Non-conservative mutations at the binding interface disrupt binding, whereas conservative substitutions are tolerated. In the text, we now clarify our strategy in greater detail (p9 line 219):
"To confirm that the designed interfaces formed as predicted, we introduced mutations at the binding interface of our PD-L1 minibinders and tested whether these alterations would disrupt the interaction with their target. Specifically, non-conservative substitutions were introduced by replacing predicted key hydrophobic residues with bulky polar amino acids, expected to induce steric clashes and disrupt hydrophobic interactions critical for binding."

The selection of residues for interface mutagenesis was guided by the predicted complexes and the expected introduction of steric clashes. We focused on single-point mutations, as disruption of binding by an individual substitution provides more direct evidence for the

functional relevance of specific interface residues, whereas multiple mutations carry a higher risk of perturbing the overall scaffold.

(page7, line9-10)
How did the authors optimize the Alanine patches? Since generative AI often suggests biased amino acid sequences, it would be beneficial to describe such findings for unlocking the broader potential of AI-based protein design.

We replaced these poly-Alanine patches with more diverse polar residues, most commonly alanine to serine substitutions, and additionally introduced residues with longer polar side chains (e.g., lysine or aspartate) while balancing charge. This strategy reduced sequence bias and improved physicochemical properties without affecting the binding interface. These experiments served as a proof-of concept how the non-binding interface can be adjusted.

(page7, line11-18)
Supplementary Figure 3 shows that the yield is improved by optimizing the Alanine patch. And for BN_1358 and BN_1358_AlaOpti, the change in fluorescence signal intensity due to DSF results is large, and the physical properties appear to be better for BN_1358_AlaOpti (BN_1358 may be a naturally denatured protein). Do the authors not think that this is the result of optimization of Alanine patches?

There is clearly a large change in the melting temperature between the two variants of BN_1358. We agree that this is due to the "optimization" of the Alanine patches, but we can only speculate what might be the molecular reason for this. The gelfiltration in panel b) shows that BN_1358 elutes at a smaller elution volume than BN_1358_AlaOpti. This points towards dimer/multimer formation, which might stabilize the AI-binder and lead to a higher thermal stability. However, both variants of the AI-binder were tested in SPR experiments and showed very clear binding to human PD-L1 (see Fig. 3j, Supplementary Fig. 4, 5). For this reason we do not believe that BN_1358 is a naturally denatured protein.

(page10, line3-4, Figure 5a)
The rescoring process seems a bit complicated. Boltz works with only amino acid sequences as input, but why do the authors have to go through Chai-1 ESM to enter Boltz-1? Also, why do the authors have to go back to Chai-1 MSA after entering Boltz?

We apologize that our previous diagram was confusing. The key point is whether rescoring requires prior multiple sequence alignment (MSA) or not. We decided to create a revised version that focuses more on the type of input than the exact piping of the data for simplicity. We hope the new diagram illustrates better the different requirements of the models and improves readability.

(page10, line24-28)
The authors state that Chai-1 is superior to pAE by comparing Group A, B, and C, respectively, but if they focus on each model and evaluate its performance, shouldn't they

compare A+B and B+C?

The aim of this analysis was to assess model performance in cases of disagreement. B shows that both models are capable to correctly predict the majority of top binders, indicated by a high confidence score. A and C illustrate cases where the models disagree and one model indicated high confidence while the other reported lower confidence. Comparing these groups, we showed that a high Chai-1 ipTM score seems to be more reliable in these cases than a low AF2 pAE score. However, we believe that both models are good tools to select the most promising candidates in silico and users might even consider running both models if their computational resources allow for that.

(page 11, line 15-20)
What would happen if strong-binding Binders (MiB, HM9) were added to this plot? If the positive control results are the same for AF3 and Chai-1 ESM, then it can be said that common results are obtained at least for strongly binding Binders. If so, couldn't ΔipTM be used as an indicator other than pAE for protein design?

The question is related to previous Figure 6h, i; in the revised manuscript Supplementary Figure 6d, e.

We introduced ΔipTM as the change in ipTM upon introducing non-conservative mutations at the binding interface and therefore ΔipTM represents a relative measure of how sensitive a predicted complex is to perturbations. Importantly, this metric requires a reference structure (i.e., the original binder–target complex) and evaluates how deviations from this reference affect model confidence.
In contrast, the design pipeline operates in a *de novo* setting, where such a reference is not available and candidates must be ranked based on absolute confidence metrics. For this purpose, scores such as ipTM or pAE interaction are appropriate, as they directly reflect the predicted quality of a given interface without requiring prior knowledge.
Consequently, while ΔipTM is informative for assessing the robustness and specificity of a given interface and for identifying deleterious mutations, it is not suitable as a standalone metric for initial candidate selection or ranking in protein design.

Also, Figure 6h and 6i include sequences that showed no difference in binding affinity in experiments. What would happen if the graph was limited only to sequences where binding affinity clearly decreased due to mutation? This might make Chai-1 appear to represent the experimental results better than AF3.

We agree that Chai-1 appears more consistent with the experimental data in this instance, we caution that the limited number of tested interface mutations precludes drawing definitive conclusions.

Additional remark: In the previous version, we noted that in Supplementary Table 7, the sequence and AF3 ipTM scores of the PD-L1 minibinder BN_401 were erroneously duplicated from BN_1028. This has been corrected in the revised version, including in the corresponding plots in Supplementary Figure 6d,e. The conclusions remain unchanged.

(page 11, line 23-25)
What are the advantages of detection reagents using AI-binders compared to those using conventional antibodies?

We now discuss the potential advantages of AI-binders in greater detail but also address the caveats, which we also encountered in our study (now p29, line 694):
"…, AI-designed protein binders offer an attractive opportunity, as their sequences and structural models are openly accessible and can be iteratively improved by the research community. They can be produced cost-effectively at high yield in microbial systems, and their small size enables rational design with defined binding interfaces. Moreover, their stability, including resistance to thermal stress, may facilitate improved shelf-life and handling. Together, these properties suggest that minibinders could serve as useful alternatives for applications such as flow cytometry. Our PD-L1 quattrobinder data provide proof of principle that AI-designed minibinders can perform on par with conventional antibodies in flow cytometry. However, the variability observed across targets, including limitations in specificity or sensitivity in certain settings, underscores the importance of stringent validation and further refinement."

(page 12, line 22-26)
Among the anti-CD257 binders, 616_606 seems to be the most promising, so why was a quattrobinder not created?

Indeed, we tried it but the CD276 binder 616_606 produced very poorly in *E.coli* and thus we could not purify sufficient amounts for biotinylation and quattrobinder assembly.

Minor point

(Supplementary Figure 2a)
What are FSC-A, SSC-A, and FSC-H?

FSC-A (forward scatter area) reflects cell size, SSC-A (side scatter area) indicates internal complexity or granularity, and FSC-H (forward scatter height) represents the signal peak used for doublet discrimination.

(Method)
Several subscripts that should be present are shown in normal size.
Example: page 18, line 10: MgCl2

We have corrected this in the revised version and also carefully checked subscripts throughout the manuscript.

Reviewer #2 (Remarks to the Author):

This is a unique and compelling manuscript that presents a workflow bridging artificial

intelligence (AI)-based protein design and experimental validation across both cell–molecule and molecule–molecule interactions, with a particular focus on immunoglobulin superfamily (IgSF) cell surface proteins involved in immune checkpoint inhibition (ICI). AI-driven protein design is a rapidly evolving and inherently challenging field with significant potential for novel therapeutic applications. From the perspective of a cancer biologist and IgSF researcher, I would like to raise the following points that should be clarified or expanded upon to strengthen the manuscript.

We thank the reviewer for the very positive evaluation of our work.

1. N-Glycosylation and IgSF Protein Diversity
It is well established that more than half of IgSF proteins exhibit structural and functional diversity due to N-glycosylation, particularly within their variable Ig-like loops. In fact, a potential N-glycosylation motif (N-X-S/T) is present within the Ig-like V-type loop of PD-L1, conserved across human and mouse orthologs (Fig. 3g).

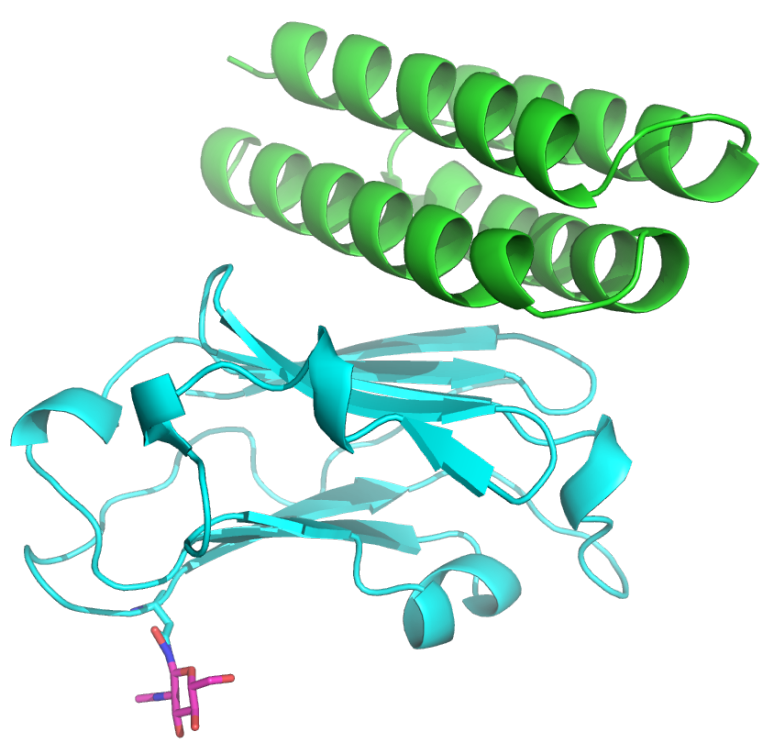

Indeed, we considered glycosylation sites in PD-L1, CD276, and VTCN1 as potential caveats during the initial design process. The AF3 model shows BN_286 (green) in complex with the glycosylated (magenta) Ig-like V-type loop of PD-L1 (cyan).

Therefore, the hydrophobic residues used as hotspots for the RFdiffusion design process are located opposite the evolutionarily conserved glycosylation motif N-X-T (Homo sapiens, position 35 in Q9NZQ7-1). Accordingly, all PD-L1 minibinders generated in this study (as well as those reported by Watson et al.) bind opposite the glycosylation site.

We now clarify this in our revised manuscript (now p6, line 151):
"…PD-L1 was included as a benchmarking target, since Watson et al. previously designed AI-minibinders against PD-L1 using RFdiffusion7, enabling us to select a patch of hydrophobic amino acids at the binding interface of their validated PD-L1 minibinders as hotspot for our design process. Furthermore, this hotspot was located opposite to an evolutionary glycosylation site (N-M-T, position 35 in PD-L1 Q9NZQ7-1) avoiding potential interferences by glycosylation. …"

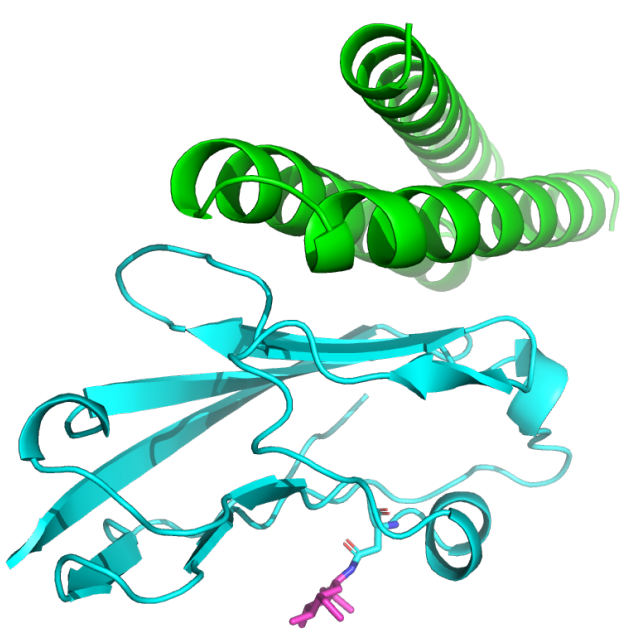

We applied the same strategy for the design of AI-minibinders against CD276 and VTCN1, selecting hotspots that do not overlap with documented glycosylation sites.

To illustrate this, the AF3 model shows BN_486-1519 (green) in complex with the glycosylated (magenta) Ig-like V-type loop of CD276 (cyan). The glycosylation site N-A-S-corresponds to residue 104 of CD276 Q5ZPR3-1.

We also clarify this in our revised manuscript (p7, line 160):
"…In contrast, the design of candidate binders against CD276 and VTCN1 was less efficient, despite testing multiple hotspots (Supplementary Table 1). These hotspots were defined by distinct combinations of hydrophobic amino acids, which may form favorable interaction patches that usually increase the likelihood of successful minibinder design by RFdiffusion, and were intentionally positioned opposite glycosylation sites to minimize potential interference. …"

In this study, AI-designed binder proteins were expressed in E. coli for SPR analysis, in HEK293 cells for flow cytometry, and in T cells for CAR-T applications—systems that differ substantially in their capacity for post-translational modifications, especially glycosylation. These differences may influence binding activity and could account for variability observed across experimental systems. The authors are strongly encouraged to examine and discuss potential differences in N-glycosylation among representative AI binders, and to address how such modifications might affect molecular interactions involving the IgSF proteins studied.

As suggested, we examined the minibinders, that we focused on in greater detail in our study, for the presence of N-glycosylation motifs N-X-S/T (Supplementary table 10).

Target	class	protein sequence	N-X-S/T
PD-L1	BN_1339	AEAWLAEIREIVSRAVEAAQAGDVETLEAAVEEARALAERAPPEVRPAAENAAAFNQLLLELTKERLAEKA	no
PD-L1	BN_869	SEEAQAAVRFARNLLEVARLRAEEDPARGRAIAANLEVAARTLAEKHAGLPEAEELRRLAAEAAALAAELA	no
PD-L1	BN_286	SNEEKAEDLRFMARAVLGVGATASAAETAAFLNDVAAAAARLDPEAGAAVAAIGAAAAASGDAKAAAKAAAAVLEAAAEEFL	no
PD-L1	BN_1318	SAAAAAAARGEAAVASLTATGAAIVAAAKKDPSEAASMVRAVKNLGQAYANRVPPEYAAKVLAVAATYAAQIAAIM	no
PD-L1	BN_1265	SENEEAAKALTRAAEAAARYLAEKYGELAKEAAKLIAKAAEADAETAVKYLEEAVKLLDEAGKKTGATKETDALKAMAEKAAELKKA	no
PD-L1	BN_1136	SAAAAARARTAAANAAALAKKMLEAGVSPENARALVEAAAARLTPEAGAALRAEGEALVAEYAARREAEAA	no
PD-L1	BN_482	SESEEWIKEITDAANQAVALAKAGKTAEAYALLDATAAKAAAKSPSRGALAKGVADAMRVYIDKYAAKKA	no
PD-L1	BN_1481	SSDEDYAALVEGMALASQTYAQARALIAATGAAGGAQARALAQAAAAQLRAAGAKYPSRAAQYDALADGLVRTVTAAAA	no
PD-L1	BN_1493	AAESEALRQQAIDLAQKAAEIAADDENAEEVKARIDAAIAHLQAGDYAAAAADLLAAAKLASPEARGYILKALLKAMEAA	no
PD-L1	BN_416	SEEAARILAETLGTISAQTAAAKAAAAAGDVEAARAAAAAAARAAAEEGAKRLEELGNKSGAEALRSIGAATAAHIKALAAKAAA	yes
PD-L1	BN_321	GSNFEKAYYAGEAYSYAYAAVKEAKALVKALGDAAKPLLDEAEALLKKIEEVLKNGNLEEAKKMLEEAKALLEKVKAA	no
PD-L1	BN_14	AAAVAAAIEAAMRAAMPSDPAKAAELAALIAAKGLAGAKEAARKAMELFREDPELGKQAFAGVDAARAILAAA	no
PD-L1	BN_895	MTPEAKSAFAAAQSLADAIKLGAKEGLLTPETRAALVEKLRAAAAEAAALDPAIAPQAAALAADAEAAAA	no
PD-L1	BN_573	SAEQAALEAKAKEAALKAWEAAKQYKKTGYQEFKDAAKEYAYAAIAYFRGDTAAGKKYRDAADKLWAKAQAIAAA	no
PD-L1	BN_863	SRAERLIEEAERLAKEAKEAAKRVADSPVAPRAKALADATEAFAKGLKAAAAAGGSMEDQEALLRAARNTAEAAKILAEEAEAARAA	no
PD-L1	BN_1034	DQAFTDAAAAAKALAAIPKYKALGDSLAARVAALAAAAAAARAAGAADPATAAEDAAAAAALAAAAKNIGDFAKAMLKAIEEDE	no
PD-L1	BN_1358	AAAESAALAAALAALRKTEVGPFARQYIIYRLLAQVAEAKGDEETAKRAKAKAEELAKAYPEAAAAADAALALAQAALAA	no
PD-L1	Watson_76	MYEVVIEGEKSVAEFIKLIAEQLGAEAEVEGDKAVIRTERREDAERLAEAAKRFGAEAEVRE	no
PD-L1	Watson_5	EEDLEKLVELARKALEHIKRAAEYSKEDESKMAEELAKAVALASEISKIDVEVAKLVLKAVNAVMKGDRLEEGVKLAEEALKRAEEKK	no
CD276	BN_616_2162	MLTPEQLQASINAIKAATRKAVEMARAGNEESAEKLLEVARDIAEALLKTDPEAGKEALKIVEEGEEEARKAAEEVRA	no
CD276	BN_619_2329	SALTKKIEEKATKLVEEAAKTTPEEARKLATEALEIAKQSPEALSALAVELLKAAAKATPEVAAVLREVAAQAMRTAIEIARA	no
CD276	BN_616_606	SPLAQAQADLLRRTLAALRRSVERAEELAREDPEAGAARLRALAQSAEVVLEAARGLAAQDPALGAETVAAAREVLERAREAEERAR	no
CD276	BN_486_1519	DEVAKLVDETAAAARAAVAAAARTAAAGTPEQSAALSSLMVATLQAVGDAYRALTDPAEAKAAAEKARALAEELAARTAEIQA	no
CD276	BN_486_1043	AAEKEAEAARLERLMQAYVAAAMAGAARVTGNEATAAAQLAKARALDPEVAGELEAAVLADPQAALAEALERARAAEERLRALREE	no
CD276	BN_488_2450	AEERERLTEKARLALEAAESLVAEAERTSPERAAALARSALRFAAEAERYAKEAGDEEIAARAREVRARAEAIL	no
CD276	612_1410	MEEIRALAEEALATAEAELATMRAAQEAGDVGTAVDALRVLFRTANEAIRRLRELGGPEEYIERLEAARREAQAILAATARRAIAQA	no
CD276	614_884	MAVSDTLATNLATAERLLQRGIELAKTDPQAGIAQLRAAEILAQGTAAAAAAAGDTATAARAQAVAERARQAIEEV	no
CD276	619_766	MLGAEWARQQARRTAERAAELLAADPAAGAAAFARARTIVELAKGIDPEVGKAAQAEVEAIEAQAKAAAAAA	no
CD276	621_606	DVAEVLVRTAQAVARQAQAAAALSPEEALAVAQANQQTVAVLLAAAKAAGSAEAVAAAQQAQATVAAVIAAAQARLAA	no
CD276	BN_1028	AAAIAATKAELAKGKALLAKAKAAPAAAWNEALAAELRAAANALNRAATALLAAGGDTALAVEATETAVELLKAAGEINRA	no
CD276	BN_401	MEKVEKAKKGLEQATELAKAMVPEKAEEFEKIRKEGVEEAKKAAGDPEKAAEILAKARNEILRNGIKAAMAAAKAAA	no
CD276	HM9	DEEWEFRILERTAKALIRQNDRRMLKVLLESVEYFLRKTGDPRARELFERIKRFLD	no
CD276	mb	DEERELRILERTAKALIRLNDRRLLKVLLESLEYFLRKTGDPRARELFERIKRFLD	no
CD276	HM9_1_0-197	DEEWEFGILHRTAKALIRQNDRRMLKVLLESVEYFLRETGDPRARELFERIKIFLD	no
CD276	HM9_1_14-10	DEEWEFGILHRTAKALIRQNDRRMLKVLLESVEYFLRETGDPRAHELFERIKRFLD	no
CD276	HM9_1_33-45	DEEWEHRILERTAEALIRQNDRRMLKVLLESVQYEYRKTKDPRARELFEMISNHLD	no
CD276	HM9_1_39-19	DEEWEFGILHRTAKALIRQNDRRMLKVLLESVEYFLIETGDPRARELFERIKIFLD	no
CD276	HM9_1_39-4	DEEWEFGILHRTAKALIRQNDRRMLKVLLESVEYFLRETGDPRARELFERIKYFLD	no
CD276	HM9_1_40-28	DEEWEFGILHRTAKALIRQNDRRMLKVLLESVEYFLRETGDPRARELCERIKIFLD	no
CD276	HM9_10_31-15	DEAWEFRILERTAKALIRQNDHRMLKVLCESVEYFLRKTTDPRATQLFERHCRFLD	no
CD276	HM9_10_50-38	DEEWEFMILERTAKALIRQNDHRMLKVLCESVEYFLRKTTDPRAMQLFERHCRALD	no
CD276	HM9_13_50-30	DECWEFTILERTAKALIRQNDRRMLKVLQESVEYFLRKTGDPRARELFERIKRFLD	no
CD276	HM9_14_37-6	DEQWEFRILERTYKALIRQNDRRMLKVLAESVEYFLEKTGDPRLRELLVRINRFLD	no
CD276	HM9_15_18-12	DAEWEFSILERTAKALIRQNDRRMLKVLLESVEYFLEKTGDPRARELFDRIKRFLP	no
CD276	HM9_18_45-33	DEDWEFRILERTAKALCRQNDRRMLKVLLESVEYAMIKTGDPRARELFQRIKRFLD	no
CD276	HM9_2_1-17	DEQWEFRILERTAKALVRQNDRRMLQVLLESVEYFLDKTGDPRARELFERIKRFLD	no
CD276	HM9_20_1-31	DEEWETNILERTAKALIRQNDRRMLKVLLESVEYFLRKTGDPRARELFERIKRFLD	no
CD276	HM9_3_35-17	DEEWEFRILERTAQALARQNDRRMLQVLGESVEYFLRKTGDPRARELFERIKRFLD	no
CD276	HM9_3_39-26	DEEWEFRILERTAKALIRQNDRRMLKVLSESVEYFLRETGDPRARELFERMKRFLD	no
CD276	HM9_9_16-32	DEEWEFRILERTAKALIRQNDRRMLKVLLESVEYELRKTGDPRARELFERIKYFLD	no
VTCN1	599_284	AEVEAFKAAAEKYYKKAKEYFEKAKKTGSKSLYLKGASYAELALHNLENAAKAAAAKDPAEGAKLQKELKETKEELDKLYEEARKAAV	no
VTCN1	597_63	GEAKAEELIKKAKEALREFAETGSLEALDKAEEYAEEISKFTPEVRAKAAEELEKVGEEGSPAARAVAKNLADYLK	no
VTCN1	600_1872	HSEEELKLLERMREIRKRAKEAVKLLKELGEKELGEKYAKEIADLLYQSIDDPENQEKYVEEAEAKAEELEKLAEEAKKKAEE	no
VTCN1	600_1035	EKLEEAEKERDKAVDKLAALVAKALGPEAGAKARAAAKKGLAALKAVVAEAAKTSGLTAELAKALHEVYQATAEAELLKKK	no
VTCN1	601_1497	SMEEEEQRRRAIEELKRLMELADKSPSEIAKATREAVAATPLYAERIRKVGTAMMFNPEEGKKLAEEAIKELE	no

Only one PD-L1 minibinder, BN_416, contained a N-K-S motif, but BN_416 was not central to our experimental workup and we only reported data on cell surface expression in mammalian cells (Fig. 3b).

In addition, we analyzed the frequency of asparagine (N) across the binder pool (improved pool with 2000 minibinders) and observed a marked depletion, with an overall frequency of 1.48% (2,351 out of 159,046 residues). This is significantly lower than expected under a uniform amino acid distribution (5%; binomial test, $p \ll 0.001$), with the global amino acid composition also deviating from uniformity (chi-square test, $p \approx 0$). Moreover, N frequency is substantially reduced compared to the reported median frequency in the human proteome (~4.3% - 4.4%), indicating a pronounced depletion of N in the designed sequences.

We also analyzed the frequency of N-glycosylation motifs (N–X–S/T) across the binder pool and observed 109 motifs across 155,046 possible tripeptide windows (frequency 0.000703). This was slightly lower than expected based on the observed amino acid composition (expected 119.3 motifs), corresponding to a modest reduction (fold change 0.91); however, this difference was not statistically significant (binomial test, $p = 0.38$). Consistently, a permutation-based analysis preserving sequence composition yielded a similar expected motif frequency (mean 120.8 motifs; $z = -1.12$, $p = 0.27$), indicating no significant deviation from expectation.

In summary, RFdiffusion-based scaffold design combined with ProteinMPNN-based sequence (side chain) design results in a strong and statistically significant underrepresentation of asparagine (N). In contrast, the frequency of N–X–S/T glycosylation motifs is not significantly reduced beyond what would be expected based on the underlying amino acid composition. Nevertheless, such motifs remain less frequent than in the human proteome.

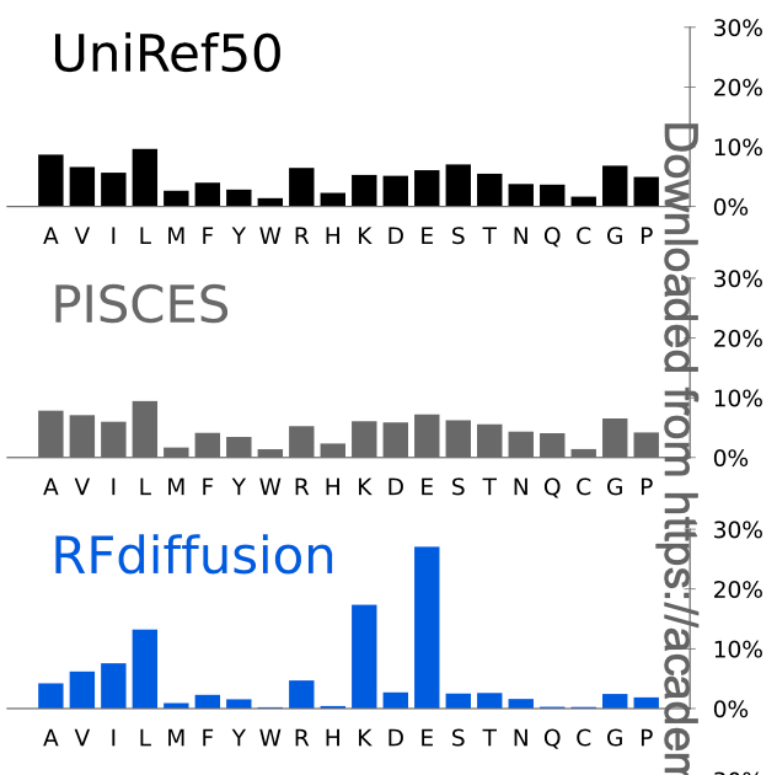

Downloaded from https://academ

This is consistent with a recent benchmarking study comparing various models (image shows part of figure 2 from the cited preprint). UniRef50 is a non-redundant protein sequence dataset derived from UniProt.

Barnett et al., Benchmarking generative AI protein models, Genomics, Proteomics & Bioinformatics (2026), DOI: 10.1093/gpbjnl/qzag014.

We now report on these analyses in our revised manuscript and discuss glycosylation as an important aspect to consider when optimizing AI-minibinders for applications, in particular in the case of discrepant results between bacterial and mammalian expression systems (now p10, line 252): "… We immobilized a fragment of human PD-L1 containing the extracellular domain purified from HEK293 cells to the sensor surface and injected concentration series of the minibinders BN_286, BN_482, and BN_1358 purified from E. coli. Of note, E. coli lacks the N-glycosylation machinery, which may lead to differences, however, none of the purified PD-L1 minibinders contained a canonical N-X-S/T N-glycosylation motif (Supplementary Table 2). Analyzing the entire pool of minibinders revealed a marked underrepresentation of asparagines compared to the normal human proteome (1.48% vs. 4.3% – 4.4%; binomial test, $p \ll 0.001$) in line with recent analyses benchmarking various protein design

algorithms22, thus minibinders designed by RFdiffusion exhibit an underrepresentation of potential N-X-S/T N-glycosylation motifs. …"

2. SPR Methodology and Data Presentation
The authors performed surface plasmon resonance (SPR) analysis using Biacore™, providing robust and quantitative assessments of protein–protein interactions. The data presented in Figures 4h–j and Supplementary Figures S3a–c are convincing and support the study's conclusions. However, given the importance of using a series of five concentrations of AI binders for interpreting kinetic parameters, the exact concentration ranges should be clearly stated not only in the Materials and Methods section but also in the main figures or their legends to ensure clarity and reproducibility.

As suggested we added the information to the legends as follows:

(Figure 3): "…**h–j** SPR (surface plasmon resonance) sensorgrams showing raw binding data (green) and fitted curves (black) used to determine binding affinities (KD) of PD-L1 minibinders BN_286, BN_482, and BN_1358 binding to human PD-L1. Five-fold concentration series were used ranging from 1.6 nM to 5,000 nM (BN_286), 0.32 nM to 1,000 nM (BN_482), 0.064 nM to 200 nM (BN_1358). …"

(Supplementary Figure 5): "… **a-c** SPR (surface plasmon resonance) sensorgrams showing raw binding data (colored dots) and fitted curves (black) used to determine binding affinities (KD) of poly-Ala patch optimized (AlaOpti) PD-L1 AI-minibinders BN_286, BN_482, and BN_1358. Five-fold concentration series were used ranging from 6 nM to 5,000 nM (BN_286 AlaOpti), 0.064 nM to 200 nM (BN_482 AlaOpti and BN_1358 AlaOpti). …"

Additionally, it would be important to explicitly mention that the protein fragments used for SPR analysis were produced in E. coli, which lacks N-glycosylation machinery. This contextual detail is essential given the topic of this study.

As requested we now mention the source of the proteins/protein-fragments, bacterial versus mammalian, used for SPR analyses within the text of the revised manuscript, in addition to the methods and supplementary tables (now p10, line 252): *"… We immobilized a fragment of human PD-L1 containing the extracellular domain purified from HEK293 cells to the sensor surface and injected concentration series of the minibinders BN_286, BN_482, and BN_1358 purified from E. coli. Of note, E. coli lacks the N-glycosylation machinery, which may lead to differences, however, none of the purified PD-L1 minibinders contained a canonical N-X-S/T N-glycosylation motif (Supplementary Table 2). …"*

3. Target-Dependent Efficacy of RFdiffusion
The authors demonstrate that the RFdiffusion model generated numerous candidate AI binders for PD-L1, but relatively few for CD276 and VTCN1. Based on these observations, they conclude in the Abstract that RFdiffusion exhibits target-dependent efficacy in generating high-quality designs. While I agree with this data-driven and neutral conclusion, the manuscript would benefit from further mechanistic interpretation. PD-L1 and PD-1 form a well-characterized, high-affinity interaction that is central to ICI therapy, whereas definitive binding partners for CD276 and VTCN1 remain controversial. Several have been proposed—e.g., BTLA and TREM-like receptors for CD276, and TLT-2 for VTCN1—but their

physiological relevance is less well established. Additionally, ALPHA (Amplified Luminescent Proximity Homogeneous Assay) and other data have shown that the PD-L1–PD-1 and CTLA4–CD80/CD86 interactions are substantially stronger than many other IgSF interactions, although ALPHA is less quantitative than SPR (see: Verschueren et al., Cell 2020, 182(2):329–344.e19; Wojtowicz et al., Cell 2020, 182(4):1027–1043.e17). In light of these differences, I strongly encourage the authors to provide a hypothesis or commentary on why RFdiffusion may exhibit higher efficacy for PD-L1 than for CD276 or VTCN1. Such a discussion would substantially enrich the mechanistic insight and broaden the impact of the paper.

We thank the reviewer for this insight and now provide a detailed section in the discussion also citing the suggested references (now p26, line 612, references 37 and 38):

"… In our hands, RFdiffusion-based design was markedly more efficient for PD-L1 than for the related B7 family members CD276 and VTCN1. Given that RFdiffusion performs best when targeting hydrophobic hotspot patches, one possible explanation is that PD-L1 presents a more prominent and accessible hydrophobic binding interface for minibinders compared to CD276 and VTCN1, based on available structures and models. Notably, the PD-L1–PD-1 interaction is substantially stronger than most other immunoglobulin superfamily (IgSF) interactions, while natural ligands for CD276 and VTCN1 remain controversial[37,38]. In this context, the observed differences in design efficiency may reflect intrinsic properties of PD-L1, including an interface optimized for high-affinity interaction with PD-1, particularly as the binding interface of our minibinders overlaps with the PD-1 interaction site. …"

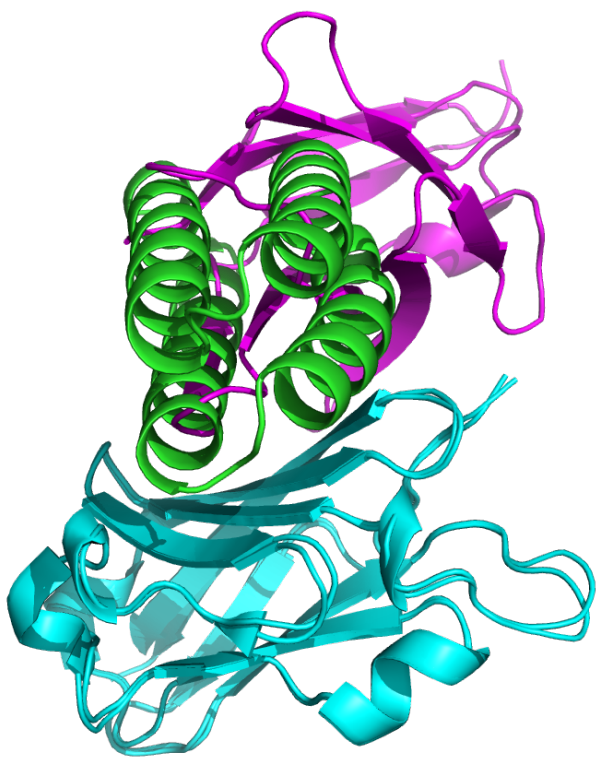

To support our discussion by structural modelling, the binding interface of PD-L1 Binder BN_286 (green, as an example) and PD-L1 (cyan) overlaps with the binding interface of PD-1 (magenta) and PD-L1. PDB:3BIK was used for the PD-L1-PD-1 complex.

Reviewer #2 (Remarks on code availability):

I have reviewed the code, but the contents and total volume are essentially same as the manuscript that was submitted to Nature Communications in this time, thank you.

In addition, HM9 variant designs (cif files) and genetic algorithm code are available in Zenodo: https://doi.org/10.5281/zenodo.19167586.

Zenodo link for review process:

https://zenodo.org/records/19167586?preview=1&token=eyJhbGciOiJIUzUxMiJ9.eyJpZCI6ImE4NzVkOTMzLWNhMzItNDZjMy1hODg4LTRjOGIxOTZiZjUwMCIsImRhdGEiOnt9LCJyYW5kb20iOiJjZmZlMzRiZmQzM2U2ZGFhNGNiYTQ5OGE4YmFjNDUwMCJ9.wJ-

ktUG9-Z-EIhAvb6tUfBwVuxUAXlDZDr1uu0WXionmTQihaqCzJBWOVqynE7QGYfBt0CHu_a9Wr9JMyQY0HQ

The pdb files of PD-L1, CD276, VTCN1 AI-minibinder pools (pilot and improved) and of minibinders characterized in greater detail are available in Zenodo:

https://doi.org/10.5281/zenodo.19512851.

Zenodo link for review process:

https://zenodo.org/records/19512851?preview=1&token=eyJhbGciOiJIUzUxMiJ9.eyJpZCI6IjBjODkxMDBmLWNkZjQtNDNiZi04YmE3LTVjNjgyNWQyYjMzYyIsImRhdGEiOnt9LCJyYW5kb20iOiIzMTkwYTk5ZjNjMWRmYTNmNTMyNmFjZDQzNTY2ZDZiZCJ9.jG4hO2gQ_odRl81XAD5rlamorleHXRWxQrnNPA6gZfLXS3pJfKBcU3dCCBSZ2WOS6V9PmkqhLAJ05gDzZyxolw

This information has been added to the corresponding methods section.

Reviewer #3 (Remarks to the Author):

This study incorporates de novo AI-designed protein binders into second-generation CAR-T cells targeting PD-L1 and CD276. The authors show antigen-specific T cell activation in vitro, including against endogenously expressed targets. The work supports using AI binders in CAR design, but several key gaps remain.

Major Concerns

1. The most critical missing piece is the absence of in vivo validation. The paper includes solid in vitro cytokine data but never tests whether these CAR-T cells actually work in vivo. There's no evidence for expansion, persistence, trafficking, or tumor control. A basic xenograft model with PD-L1$^+$ or CD276$^+$ tumors in NSG mice would have been enough to test specificity and therapeutic potential. Right now, there's no way to know whether these CARs can eliminate tumors or avoid off-target toxicity.

We would like to point out that proof-of-concept for minibinder-based CAR-T cell therapy in vivo has been already established by Xia et al., who demonstrated the activity of EGFR- and CD276-targeting minibinder CAR-T cells in immunodeficient mouse models (Nat. Biomed. Eng. 8, 1634–1650, 2024). The HM9 and mbCD276 minibinders used in our study were derived from this work, which we therefore cited several times in the manuscript. We recognize that the relevance of this prior in vivo evidence may not have been sufficiently clear in the previous version, and we have now emphasized this point more explicitly in the revised discussion section (now p.28 line 659-663): *"A limitation of our study is that we do not provide in vivo validation of the minibinder-based CAR-T cells. However, proof-of-concept that de novo-designed minibinders can serve as CAR recognition domains in vivo has already been established by Xia et al., who demonstrated the activity of EGFR- and CD276-targeting minibinder (HM9) CAR-T cells in xenograft models in immunodeficient mice[27]"*.

Therefore, we have also added a dedicated section to the discussion in which we clearly acknowledge the absence of in vivo validation as a limitation of our study and outline key next steps for further development. In particular, we now highlight the need for rigorous studies in syngeneic models to address minibinder immunogenicity and its impact on CAR-T cell persistence, as de novo-designed minibinders are artificial protein scaffolds that may elicit immune responses in immunocompetent mice (now p28 line 663-681).

We would also like to emphasize that the main objective of our study was to establish a scalable framework for de novo minibinder discovery and rigorous downstream validation, including assessment of target selectivity and demonstration of functional applicability across different formats. In this context, quattrobinders and CAR-T cells served as proof-of-concept formats illustrating potential downstream applications of the platform. In the revised manuscript, we have additionally included a substantial new body of work on the biochemical optimization of minibinders for improved CAR surface expression, directly addressing the concern raised in comment #2.

2. Not all CAR constructs expressed well on the surface. MiB and HM9 in particular showed poor trafficking, probably due to binder misfolding or structural instability. This is a problem that needs deeper analysis. The authors should compare the primary and predicted tertiary structures of well- and poorly-expressed binders, and consider redesigning the hinge or linker. Quantitative surface expression should be shown. If binders fail to fold properly, they shouldn't be used in CAR format.

We agree that suboptimal CAR surface expression is an important issue and that defining its underlying determinants is critical.

We therefore addressed this point in depth by adding substantial new experimental data (now p22 line 500 to p26 line 599; new main Figures 7 and 8; new Supplementary Figure 10).

Our analyses identify minibinder isoelectric point (pI) as a key determinant of CAR surface expression. Using a computational genetic algorithm-based optimization strategy, we generated and screened thousands of variants and defined a pI window (6.5–8.5) that supports robust CAR display. Importantly, these optimized variants preserved binding while improving functional activity and, notably, enhanced discrimination between target-positive and target-negative cells. In this way, optimization improved target selectivity while reducing undesired activity toward negative control cells. Together, these results show that poor surface expression of certain minibinders is not an intrinsic limitation of the scaffold itself, but can be overcome by systematic biochemical optimization outside the binding interface.

We believe that these new experimental data significantly strengthen the novelty of our study and underscore its importance for the broader bioengineering community, as there is substantial interest to further develop minibinders for CAR-T cell therapy.

3. All functional validation is based on intracellular cytokine staining (IFN-γ, TNF-α). That's a good start, but it's not enough. There is no direct quantification of killing, so we don't know if these CARs can actually kill tumor cells. That's a basic requirement.

As requested we included tumor cell killing assays (kinetics) by human CAR-T cells in our new figure 8g, h that also demonstrates how biochemical properties can also influence target-selective tumor cell killing.

4. The data show that only high-affinity binders triggered CAR activation against endogenous CD276. Lower-affinity designs only worked on overexpressed antigen. This reflects a classic problem in CAR design that affinity must be high enough for sensitivity, but not so high that it drives off-tumor toxicity. Authors should further discuss the clinical implications of this finding, and how further to optimize their model to ensure inclusion of high-affinity binders.

We now critically discuss these points in the new section p28 663-681.

Importantly, our new data demonstrate that optimization of physicochemical properties outside the binding interface can have a major impact on CAR surface expression, activity, and target-selective tumor cell killing. This provides a powerful methodology to address these challenges, enabling systematic tuning of CAR performance beyond affinity alone.

5. Both PD-L1 and CD276 are expressed on some normal tissues. This raises the possibility of on-target/off-tumor toxicity. The authors should acknowledge this and propose next steps.

PD-L1 was included primarily as a proof-of-concept and benchmarking target, as minibinders against PD-L1 have previously been described (e.g., Watson et al., Nature 2023) and we used these binders as benchmarks for our PD-L1 minibinders.

We do not consider PD-L1 as a good CAR-T target due to its broad expression on normal tissues, particularly immune cells, and, to our knowledge, there are currently no active clinical CAR-T programs targeting PD-L1.
In contrast, CD276 represents a promising therapeutic target and is being actively pursued in multiple modalities, including CAR-T cells, ADCs, and T cell engagers. Considerations regarding on-target/off-tumor expression are now discussed in the manuscript in the new section p28 line 663-681.

6. Finally, PD-L1 CARs could be functionally impacted by PD-1/PD-L1 signaling. The authors should briefly mention whether PD-1 was expressed in their model system or whether reverse signaling was considered.

D-L1 was expressed by both primary murine and human T cells. In addition, we made the intriguing observation that murine CAR-T cells expressing human–mouse cross-reactive PD-L1 minibinder CARs appeared to undergo fratricide and activation during expansion, consistent with the known induction of PD-L1 on T cells under activation and inflammatory conditions.

In our hands, retroviral transduction of murine T cells typically results in CAR expression in approximately 30–50% of CD8+ T cells, as measured by RQR8 positivity, but does not approach 100%. Unexpectedly, cultures transduced with the cross-reactive PD-L1 minibinder CARs BN_1358 and BN_482 were almost uniformly RQR8-positive, whereas BN_286 CAR-T cells showed the expected transduction efficiency of approximately 30% (Supplementary Fig. 8b). Notably, BN_1358 and BN_482 bind both human and mouse PD-L1, whereas BN_286 is

highly selective for human PD-L1 (Fig. 3e,f). We therefore hypothesize that BN_1358 and BN_482 CAR-T cells eliminate untransduced T cells within the culture, resulting in an apparent enrichment of CAR-positive cells, while also engaging each other through activation-induced PD-L1 expression. This sustained reciprocal activation may promote functional exhaustion, consistent with the reduced cytokine response of BN_1358 and BN_482 CAR-T cells upon co-culture with human PD-L1-positive target cells compared with the human-selective BN_286 CAR (Supplementary Fig. 8d).

Together, this observation further supports our rationale to use PD-L1 primarily as a proof-of-concept and benchmarking target to evaluate technical aspects of minibinder design and to compare against previously described PD-L1 minibinders, rather than as a therapeutic target for CAR-T cell development.

Minor Concerns

• Provide amino acid/nucleotide sequences of binders in Supplementary Tables.

All protein and amino acid sequences of minibinders are provided in Supplementary Tables. For convenience, the ones used and characterized in greater detail are provided also in separate table.

• Clarify HA-tag language ("CAR surface expression via HA-tag").

We specify the HA-tag language where applicable, e.g. p18, line 429"…*The human PD-L1 reactive AI-minibinders BN_1358, BN_482, and BN_286 were cloned into a second-generation CAR expression construct with an additional HA-tag for standardized detection and quantification of CAR surface expression, and the RQR8 peptide separated by a P2A cleavage site (Supplementary Fig. 8a).…*"

• Ensure consistent terminology ("CAR T cells" vs "CAR-T cells") and specify primary cells when applicable.

We now use CAR-T consistently throughout the manuscript and figures. Primary cells (human versus mouse) are specified.

• State what error bars represent (likely mean ± SEM) and what the two-way ANOVA compared.

We carefully checked all legends and specify what error bars represent. The two-way ANOVA was applied to evaluate overall differences across the indicated groups and conditions. This was followed by pairwise comparisons with multiple testing correction as stated in the legends, and for clarity, only statistically significant differences are displayed. We also describe this now in the methods section "Statistical analysis".

• State co-culture duration and E:T ratio for ICS and activation assays in the figure legends.

E:T ratios are specified in the corresponding legends.

• Clarify murine vs human CAR construct differences (species-specific domains, same binder, RQR8 epitope detection in both).

As described in the methods section "Generation of AI-minibinder CAR constructs", both murine and human share similar functional designs but with largely species-specific domains (CD8a leader, Trans-Membrane, CD28 Costim and CD3z). The shared domains are the AI binder – HA tag and RQR8 epitope. This setup allows common reagents (QBEND antibody, HA antibody) to be used to phenotype the transduced CAR products, without introducing cross-specifies domains into domains involved in the translation of antigen detection into cellular response.

• Note rationale of costimulatory domain choice (CD28ζ).

The selection rationale of CD28 as the costimulatory domains was two-fold. Firstly, it is still a clinically relevant design with FDA-approved CAR products (Yescarta and Tecartus) incorporating a CD28 co-stimulatory domain. Secondly, CD28 costimulatory CAR have shown increased short-term cytotoxicity and cytokine section compared to 4-1BB costimulatory CAR. This increased short-term activation suited the in-vitro assessment of our binders, where we aimed to identify antigen specific activation, rather than focus on any increased proliferation or persistence. Additionally, it has been shown that CD28 costimulatory CAR have a reduced affinity threshold for efficient activation, which was important given the difficulty in predicting the potentially affinity range of our AI binders. This is addressed on p19, line 435: *"CD28 was selected as the costimulatory domain because it is clinically validated (e.g., in Yescarta and Tecartus) and promotes stronger short-term cytotoxicity and cytokine secretion compared to 4-1BB32,33. We reasoned that this profile is well suited for in vitro assessment of antigen-specific activation and provides a lower activation threshold, accommodating the broad affinity range of AI-designed minibinders."*

• Briefly cite other non-antibody binders used in CAR T cells (nanobodies, DARPins) to place work in broader context, and note how AI-designed binders compare (smaller, epitope-specific, but require empirical validation for CAR expression/affinity).

We now discuss nanobodies and DARPins as further single domain recognition domains for CARs, including a brief comparison, and already approved products, and we added references to support our discussion on p28, line 683 *"Alternative binding formats such as camelide-derived variable domains of heavy-chain only antibodies (VHH, also known as nanobodies) or DARPins (Designed Ankyrin Repeat Protein) have also been explored for CAR-T cell engineering[34,43,44], offering smaller size compared to conventional scFvs, although they are generally larger than de novo minibinders. On the other hand, established humanization strategies for nanobodies may provide advantages in terms of immunogenicity, whereas the immunogenic potential of minibinders will need to be carefully evaluated in future studies. Ciltacabtagene autoleucel is a VHH-based CAR-T cell therapy approved for the treatment of adult patients with relapsed and refractory multiple myeloma targeting B-cell maturation antigen[45]."*

REVIEWER COMMENTS

Reviewer #1 (Remarks to the Author):

The authors addressed all my comments. They provided new analyses and significant textual revisions to satisfy my concerns.

We are grateful to Reviewer #1 for the positive assessment of our revised manuscript.

Reviewer #2 (Remarks to the Author):

The authors have revised the manuscript in accordance with the reviewer's suggestions and comments. Reviewer 2 has mainly focused on evaluating the authors' responses to the three major comments previously raised and is now satisfied with the appropriateness and quality of the revisions.
The prediction that potential N-glycosylation sites are located on the side opposite to the interacting interface between PD-1 and PD-L1, as well as other IgSF proteins, is particularly interesting (Comment 1). Reviewer 2 was also impressed by the observation that asparagine (N) residues are underrepresented, whereas the N-X-S/T glycosylation motif itself is not (Comment 2). Finally, Reviewer 2 appreciates the authors for adding further discussion regarding the possible biological significance of the efficiency of RFdiffusion-based prediction (Comment 3; p. 26, line 612; references 37 and 38).
At this stage, Reviewer 2 has no further comments on this potentially interesting and well-organized manuscript.

We are grateful to Reviewer #2 for the positive assessment of our revised manuscript.

Reviewer #2 (Remarks on code availability):

I have checked the preprint and recognized that the content is essentially the same as the original manuscript.

Reviewer #3 (Remarks to the Author):

The authors have substantially revised their manuscript in response to three reviewers comments. They added new data on CAR surface expression and biochemical optimization, clarified methodological details and corrected errors. However, several replies remain superficial or speculative, and the study still suffers from several shortcomings

1-The authors now say they built an accessible binder-design pipeline, but the only tweak they really describe is a filter that dumps designs with fewer than three helices. They don't provide any data that this filter actually improves success. all it does is save computing time. Without evidence that their workflow produces better binders than existing methods, it's hard to see the value of this work.

We respectfully disagree with this interpretation. Firstly, our 'accessible binder-design pipeline' does not only entail the computational part, but also the presented methods to efficiently screen and validate binders in the wetlab. As such, our manuscript contains many guidelines and methodological tweaks that we think are of interest for other experimental scientists who want to employ protein design in their research. For example, we would like to emphasize that, beyond the initial computational design strategy, we also present a follow-up approach to fine-tune the biochemical properties of minibinders and further optimize their functional performance by engineering the non-binding interface. Thus, our contribution to computational design comprises two complementary

components: a modified strategy for minibinder generation and a subsequent optimization framework for improving biochemical and functional properties (Main Figs. 7–8, Supplementary Fig. 10). Importantly, in all cases, our designs were extensively characterized for binding selectivity using isogenic pairs of wild-type and knockout cells.

As to the three-helix filter: Saving computation time is central to scaling up computational design pipelines and is particularly relevant for wet-lab-focused researchers with limited access to HPC resources, for example when designing minibinders against proteins of interest on local workstations. By filtering out de novo designed proteins with fewer than three helices at an early stage, downstream ProteinMPNN and AF2 initial-guess steps can be omitted for two-helical designs, thereby reducing computational cost. In our experience, depending on the target, this filter can reduce computation time by approximately half or even more. This is particularly relevant when the overall yield of a design campaign is intrinsically low, as observed for several targets. Thus, our approach does not aim to improve design quality per se, but rather to increase the efficiency of the workflow in terms of GPU hours required to obtain a sufficient number of designs with a high likelihood experimental of success. This applies both to binding and to structural stability, the latter being a prerequisite for productive target engagement. For clarity, we now added the following sentence to the Results section:

"… Thus, rather than improving design quality *per se*, our structural filtering step increases workflow efficiency by reducing the GPU hours needed to obtain experimentally promising designs, which is particularly relevant for scaling design campaigns and for researchers with limited HPC access. …"

From a structural perspective, it is well documented in the literature that proteins consisting of three or more alpha helices have superior stability to less structed proteins due to their possibility of forming hydrophobic cores[1–4]. Proteins composed of only two helices have a more limited capacity to form extensive hydrophobic cores, which increases the risk of intrinsic instability. This was consistent with our observations in pilot experiments. Before implementing this structural filter, we assessed the expression efficiency of minibinders. **Figure 1a** (next page) shows representative examples of minibinder designs with a high proportion (~75%) of two helical minibinders, as obtained in our pilot design campaigns without structural filtering. **Figure 1b** shows the limitation of a 2-helical minibinder to form a comprehensive hydrophobic core, in comparison to a 3-helical minibinder. These minibinders were expressed in human HEK293T cells as secreted proteins fused to a Fc tag, and protein production was analyzed by Western blot. We observed absence of protein expression in 5 out of 7 (~70%) minibinders with two helices. In contrast, absence of protein expression was rare for the majority of minibinders with three or more helices, but expression level can be variable (**Figure 1c**, next page). Based on these findings and the literature, we reasoned that incorporating a structural filter into the design pipeline would help prioritize designed proteins with a higher probability of being efficiently produced for experimental validation. This does not exclude the possibility that two-helical proteins can also be stable; however, our data suggest that their likelihood of robust cellular production is substantially lower, in line with the literature.

We discuss these aspects in more detail in the revised manuscript and have included the following references to support our statement regarding the importance of hydrophobic cores in helical proteins:
"…Nevertheless, we noticed that some of the designs had a much more compact fold, similar to affibodies[15], with three or four short alpha helices that were predicted to form stable alpha-helical bundles with an extensive hydrophopic core based on recent literature[16-18] …"

16 Ravichandran, A. et al. Structure-guided engineering of protein stability through core hydrophobicity. Protein Sci. 34, e70360 (2025).
17 Listov, D., Goverde, C. A., Correia, B. E. & Fleishman, S. J. Opportunities and challenges in design and optimization of protein function. Nat. Rev. Mol. Cell Biol. 25, 639–653 (2024).

18 Leng, X. et al. De novo designed 3-helix bundle peptides and proteins with controlled topology and stability. Chem. Sci. 16, 18632–18641 (2025).

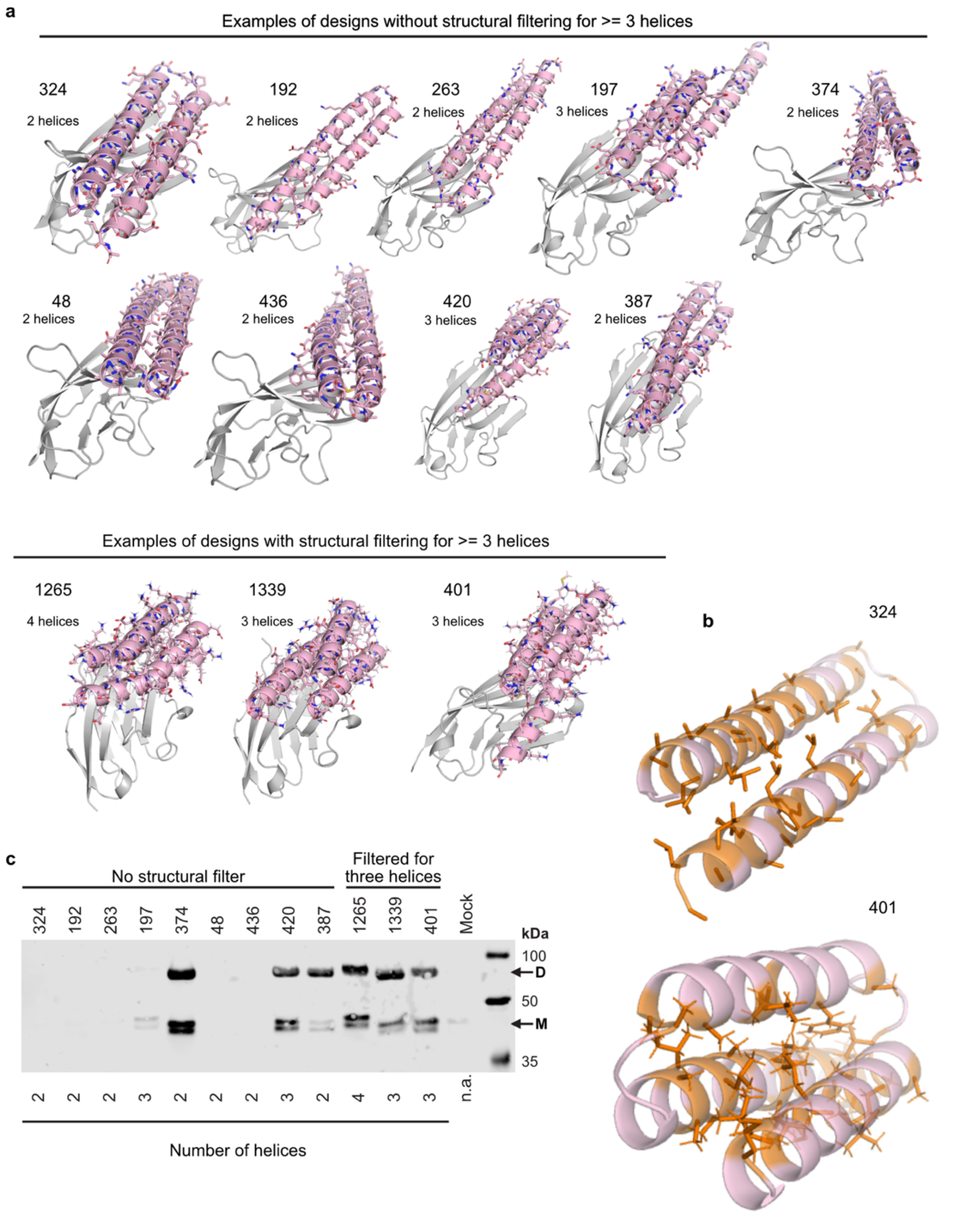

Figure 1: Structural influences on minibinder expression.
a) Representative examples of designs obtained without filtering for three or more helical minibinders (upper panels). Lower panels: a few examples of three and four helical minbinders obtained from designs with structural filtering. b) Representative examples of an unstable 2-helical (324) minibinder (helices in a parallel configuration) and stable 3-helical (401) minibinder (helices in a bundle configuration) showing hydrophobic residues in orange. The 3-helical minibinder can form a hydrophobic core. c) Western Blot staining using an antibody against rabbit-Fc. Binders appear as monomers (M) and dimers (D).

2-They limit all their scaffolds to 70–88 amino acids. That might be convenient for ordering DNA oligos, but there is no justification for why other lengths weren't explored. As someone who has seen CAR surface expression hinge on binder size and stability, I find the narrow length window troubling.

First, we emphasize that the initial scope of our study was the design and characterization of minibinders, reflecting a practical field report from a laboratory without prior experience in protein design. We developed minibinders for different applications, including their use as flow cytometry detection reagents in the form of quattrobinders, and additionally explored CAR constructs as a complementary tool to assess minibinder target selectivity in a cellular context. In response to the reviewers' comments, we expanded our CAR analyses and demonstrated how biochemical optimization of minibinders affects both target selectivity and cell surface expression. Accordingly, because the primary focus of our study was on AI-designed minibinders in general, rather than on CAR applications specifically, we restricted our designs to a size range typically used for minibinders.

Second, our central goal was to develop a pooled mammalian cell surface display platform for minibinder identification. This approach not only enables screening for whether a de novo minibinder binds its intended target, but also provides information on whether the minibinder is efficiently expressed and trafficked to the surface of mammalian cells. This is relevant for CAR applications, but also for other settings in which minibinders are used in mammalian cells, and recombinant production in general. The majority of minibinders screened by the mammalian cell surface display were stable and well expressed on the surface. The HM9 CD276 binder was obtained through iterative screening using yeast cell surface display[5], which may explain its poor surface expression in mammalian cells.
DNA oligo pools are the most suitable, cost-effective, and labor-efficient approach to generate libraries covering thousands of designs. However, the maximum oligo length available for large-scale pools imposed a practical size constraint. We therefore selected the largest oligo length available at the time, 300 bp, which accommodated minibinder designs of up to 88 amino acids. More recently, 350 bp oligo pools have become available, which would accommodate designs of up to 104 amino acids.

Third, with regard to CAR applications, nanobodies (VHHs), which are approximately 120 amino acids in length, represent established single-domain binders used in CAR formats; notably, ciltacabtagene autoleucel is an approved VHH-based CAR-T cell product. VHHs are therefore conceptually comparable to minibinders, as both are structurally compact and typically stable binding domains. To compare minibinders against this established binder class, we searched for available VHH structures bound to Ig-like V-type domains of surface receptors, including PD-L1, CD276, CTLA-4, and VSIG4, and measured the binding interface area (Å²). We then performed the same analysis for the minibinders targeting the Ig-like V-type domains of PD-L1, CD276, and VTCN1 described in our study, and compared their target interface areas with those of VHHs directed against Ig-like V-type domains. Despite their shorter amino acid length, the minibinders formed significantly larger interfaces with their respective targets (two-sided test, $p = 1.11 \times 10^{-6}$; **Figure 2**). Thus, their small size does not appear to limit their ability to establish extensive and relevant target interactions.

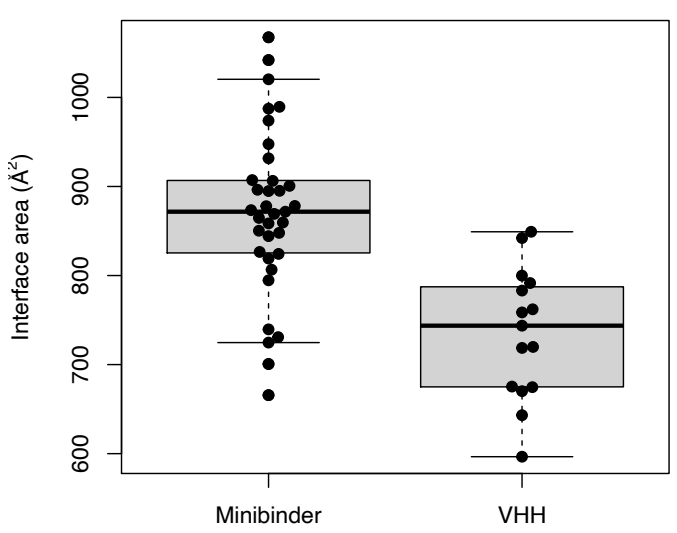

Figure 2. Comparison of interface area of minibinders with VHH directed against IgV-like domains. PDB ids of VHH-target structures: 5e5m, 5iml, 5jds, 6rpj, 6rqm, 7czd, 8aok, 8aom, 8ew6, 9ezh, 9ezi, 9ezu, 9ezv, 9ezw, 9lme.

We added the following section to our Discussion: "…Interestingly, comparing the binding interface area of 15 different VHHs with IgV-like domains (PDB IDs: 5e5m, 5iml, 5jds, 6rpj, 6rqm, 7czd, 8aok, 8aom, 8ew6, 9ezh, 9ezi, 9ezu, 9ezv, 9ezw, 9lme showed that minibinders reported in our study may be capable of forming binding interfaces that are comparable to, or even larger than, those of VHHs despite their smaller size (mean VHH: 735 $Å^2 \pm 73$; mean minibinder: 871 $Å^2 \pm 93$; two-sided t-test, 1.11×10^{-6}). …"

3-When the first round of design didn't yield many CD276 or VTCN1 binders, the authors simply picked the best hotspot as the one that produced more low-pAE designs. There is no structural rationale for why these hotspots should be good or bad. Without understanding why a particular interface works, it's hard to believe the approach will generalize. In the second round of design they got additional hits for CD276 and few candidate VTCN1 minibinders, and when they tested those individually the binding patterns were much weaker than for PD-L1. That's a long way from proving the method works broadly.

The structural rationale for selecting hotspots in the RFdiffusion design pipeline was to prioritize hydrophobic residues, as protein–protein interfaces are driven by hydrophobic interactions. Moreover, RFdiffusion has been reported to model interactions involving hydrophobic residues particularly well. This was described by Watson et al.[6] in the initial RFdiffusion report and is also reflected in the recommendations provided in the RFdiffusion github documentation.

Accordingly, our hotspot selection strategy followed these established recommendations from: https://github.com/RosettaCommons/RFdiffusion

"**Selecting a Target Site**
Not every site on a target protein is a good candidate for binder design. For a site to be an attractive candidate for binding it should have >~3 hydrophobic residues for the binder to interact with. Binding to charged polar sites is still quite hard. Binding to sites with glycans close to them is also hard since they often become ordered upon binding and you will take an energetic hit for that. Historically, binder design has also avoided unstructured loops, it is not clear if this is still a requirement as RFdiffusion has been used to bind unstructured peptides which share a lot in common with unstructured loops." [*text copied from the github repository*]

We now clarify this in the revised manuscript:
"… These hotspots were defined as distinct combinations of hydrophobic amino acids that may form favorable interaction patches and thereby increase the likelihood of successful minibinder design, in line with the recommendations of the RFdiffusion developers[7] (https://github.com/RosettaCommons/RFdiffusion). Furthermore, hotspots were intentionally positioned opposite glycosylation sites to minimize potential interference. …"

This does not imply that every computational design campaign will yield highly potent binders with equal efficiency. Nevertheless, Watson et al.[6] also demonstrated across additional targets, including TrkA, InsR, and IL-7R, that this hotspot-selection strategy can be effective and can increase the efficiency of obtaining functional designs.

4 (i)-The discussion around alanine patch optimization is speculative. in the rebuttal the authors admit they can only guess that their best binder forms dimers to explain its melting behaviour.

We agree with the referee that biased amino acid compositions are a common feature of artificially designed proteins and that understanding their consequences is of interest to the protein design community. For this reason, we experimentally investigated this aspect in greater detail and to the best of our knowledge, this study provides one of the first experimental examinations of this issue.

In the previous review round, the referee asked for our interpretation of the differences observed between BN_1358 and BN_1358_AlaOpti. We believe that the explanation provided at the time represents a reasonable interpretation of the experimental data currently available, rather than a "guess".
It is true that we cannot establish the precise molecular mechanism underlying the observed differences. However, we respectfully suggest that a definitive mechanistic dissection of the role of these alanine-rich regions would be very clearly beyond the scope of the present study. Such an investigation would require a dedicated follow-up effort, including systematic mutagenesis of the alanine patch, detailed biophysical characterization, and complementary structural studies. We believe that these experiments would constitute a substantial research project in their own right.

We hope that the referee agrees that the primary goal of the present work is not to establish the molecular mechanism of alanine-patch effects and we think that the level of analysis presented here is appropriate for the scope and objectives of the manuscript.

4 (ii)-Likewise, the isoelectric-point optimisation that improves HM9-based CARs is shown only for one scaffold. They present this as a general solution, but there is no data that adjusting pI will help other binders, and the basic variants still showed off-target killing.

With regard to the isoelectric point (pI), we re-analyzed our two minibinder pools targeting PD-L1, CD276, and VTCN1 that were designed with RFdiffusion. Using Fisher's exact test, enriched (functional) binders showed a trend toward more acidic pI values (odds ratio = 1.97, p = 0.096). Although this association did not reach statistical significance, the observed trend is consistent with our findings from the HM9 CD276 pI-adjustment screen. Of note, the RFdiffusion-designed pool was strongly biased toward acidic pI values, thereby limiting a precise assessment of pI-dependent enrichment. Consequently, the pI range of 6.5 to 8.5, which was most informative in the HM9 optimization screen, was underrepresented in the initial design pool.

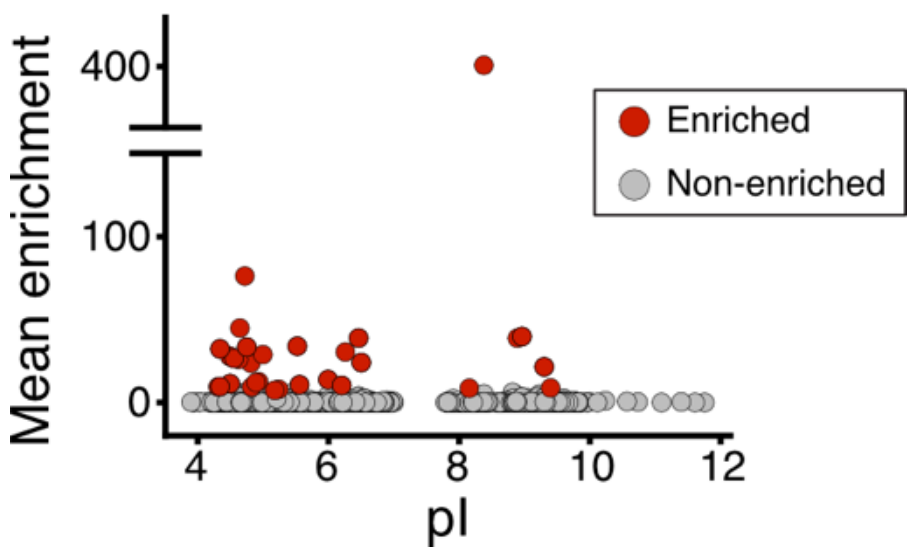

We now comment on this observation in the Discussion: "…Of note, re-analyzing our PD-L1, CD276 and VCTN1 minibinder pools designed with RFdiffusion, we observed a bias towards acidic pI values (pI < 7, 2.3 times for frequent) and also trend towards functional binding (odds ratio = 1.97, p = 0.096, Fisher's exact test). …"

Indeed, as correctly pointed out by the referee, the observation that HM9 variants with a more basic pI showed increased off-target killing represents an important finding of our study. Conversely, lowering the pI increased the selectivity window, with more acidic variants showing reduced off-target activity while maintaining target-dependent killing (Figure 8g, h). These data suggest that pI is an important biochemical parameter that can influence target selectivity and should therefore be considered when designing and optimizing minibinders for selective binding to surface molecules. To our knowledge, this represents the most comprehensive approach to date for systematically assessing

the impact of the non-binding interface configuration, based on screening approximately 6,000 computationally designed minibinder variants with a preserved target-binding interface.

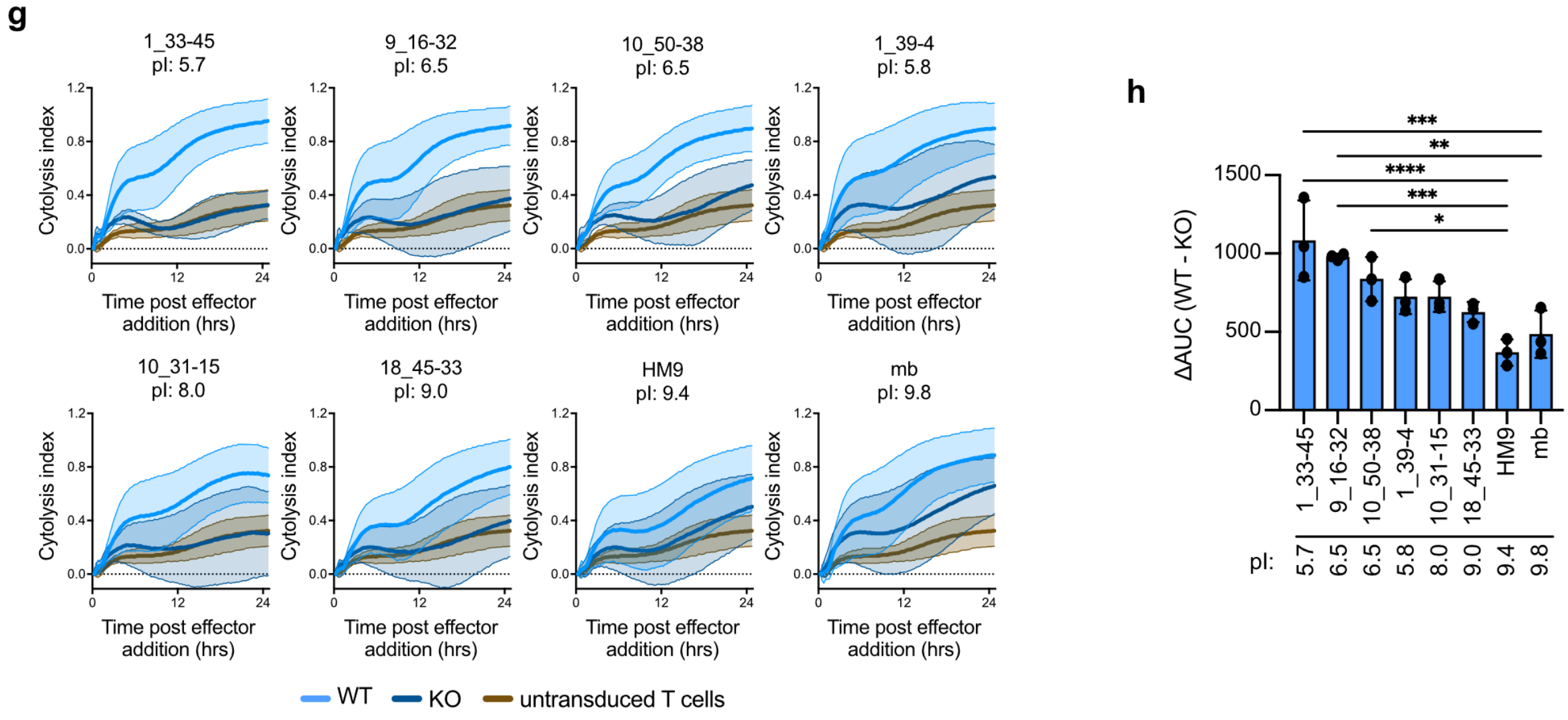

5-Despite adding lots of in vitro data, the authors still provide no animal data. They acknowledge this in the discussion and lean on another group's mouse study. For a platform that is supposed to point toward therapeutic CAR-T cells, the absence of even a simple xenograft experiment is a major gap.

As also outlined in our response to comment #2, from the outset, the primary scope of our work has been to broadly address the design, identification, and biochemical and cellular characterization of AI-designed minibinders. CAR constructs were included as a cellular format to assess minibinder behavior in a mammalian cell context, including intracellular trafficking, cell surface expression, and target selectivity. During the revision process, we expanded the CAR-related analyses, in particular to address questions regarding intracellular trafficking and cell surface expression of minibinder proteins.

In this regard, our study provides important mechanistic and practical insights and potential caveats that are directly relevant to the use of minibinders in cellular applications for many researchers. In particular, our data show that biochemical properties such as the isoelectric point can substantially influence CAR surface expression, target selectivity, and nonspecific activation (e.g. with basic pI values). Thus, rather than presenting minibinder-based CARs already as optimized therapeutic candidates, our work highlights important parameters and potential caveats that should be considered when adapting AI-designed binders to CAR formats or other mammalian cell-based applications. Together, these findings provide important practical and conceptual insights for the growing community of researchers exploring the opportunities of de novo designed proteins.

We added the following statement to Discussion after addressing the limitation of in vivo studies to clarify the scope of our work: "…Thus, rather than presenting our minibinder-based CARs already as optimized therapeutic candidates, our work highlights important parameters and potential caveats that should be considered when adapting AI-designed binders to CAR formats or other mammalian cell-based applications. …"

6-All of the binders were expressed in E. coli, which has no N-glycosylation machinery. The authors note that none of the binders contains a canonical N-glycosylation motif and that asparagine is under-represented, but they never actually test whether glycosylation of the target affects binding or whether the binders fold and function properly in mammalian cells. This is a huge gap if you are aiming for therapeutic use.

We respectfully disagree with these statements, as they do not accurately reflect the experimental design described in our study.

First, expression of minibinders in E. coli was performed exclusively for applications in which bacterial production is appropriate, namely for the generation of recombinant quattrobinders and for surface plasmon resonance (SPR) measurements, as stated in the manuscript. In these contexts, bacterial expression is unlikely to impose substantial limitations, as the designed minibinders themselves do not contain canonical N-glycosylation motifs and therefore do not require eukaryotic glycosylation for maturation or function.

Second, all target proteins used throughout this study were produced in mammalian systems, either as endogenous or overexpressed membrane-bound surface proteins on mammalian cells, or as recombinant Fc-tagged proteins expressed in HEK293 cells. Thus, minibinder binding was assessed against target proteins carrying mammalian post-translational modifications, including glycosylation. We also used CRISPR-Cas9 generated isogenic pairs of wild-type and knockout mammalian cancer cells being proficient or deficient, respectively, for the target proteins including PD-L1 or CD276. In our view, this represents the most relevant and stringent setting to assess minibinder selectivity. By contrast, testing artificially non-glycosylated human target proteins would be less reflective of the physiological target context addressed in our study. To emphasize this point, we added the following passage to the Discussion:

"… Based on our experience, CRISPR-Cas9-engineered isogenic pairs of target-proficient wild-type and target-deficient knockout cells should be incorporated into every minibinder validation campaign. Ideally, multiple independent isogenic cell line pairs should be used, as we observed cases in which a minibinder showed clean target-dependent staining in one pair but nonspecific background staining in another. …"

For further clarification, we have carefully revised the manuscript, including the main text, figure legends, methods section, and supplemental material to specify the species and production system used for each protein were appropriate.

For example, we now state in the main text:

"…Human HEK293T cells were transduced with the minibinder library, stained with human PD-L1, CD276, or VTCN1 Fc-fusion proteins produced in human HEK293 cells, and sorted by FACS (n = 3). …"

For example, we now state in the legend text to main figure 2:

Figure 2. *De novo* design and experimental screening of AI-minibinders targeting B7-H family proteins.

A–c Cartoons of the PD-L1 (B7-H1) crystal structure (PDB: 4zqk), CD276 (B7-H3) AF2 model, and VTCN1 (B7-H4) crystal structure (PDB: 4gos). **d** Boxplots with individual data points summarizing AF2 pAE interaction scores of indicated design runs. The total number of designs per target is indicated as well as the percentage of designs with low (good) scores. **e** Histogram depicting the distribution of pAE interaction scores of the best designs used for the pilot pools. **f** Representative scatter plot illustrating a subpopulation of HEK293T cells expressing minibinders from the pilot pool binding to human PD-L1 Fc-fusion protein (produced in HEK293 cells). **g–i** Scatter plots showing enriched AI-minibinders (marked in indicated colors) for PD-L1, CD276, and VTCN1 identified by NGS analysis of the respective subpopulation binding the human target protein (Fc-fusion proteins produced in HEK293 cells). **j** NGS analysis of panning by phage display showing enriched PD-L1 AI-minibinders. Minibinders also identified by mammalian cell-surface display are marked in green, binders only identified by phage display in blue, and positive controls in orange. **k** Histogram showing the distribution of pAE interaction scores for designs selected into the improved pools (n = 500 or 750) from the total initial design sets (n = 40,000, 90,000, or 75,000). **l–o** Scatter plots showing enriched AI-minibinders from the improved pool for CD276, VTCN1, and

PD-L1 identified by NGS analysis of the respective subpopulation binding the human or murine target protein (Fc-fusion proteins produced in HEK293 cells).

And, for example, in supplemental table 9:

**Protein**	**Vendor**	**Catalogue number**	**Used for**
Human PD-L1 (llama Fc-Tag) and human PD-L1 ECD Produced in HEK293 cells	Nanobody Core Facility Bonn	–	Screen of pilot pool and validation (SPR)
Human PD-L1 (human Fc-Tag) Produced in HEK293 cells	ACROBiosystems	PD1-H5258	Screen of pilot pool<br>Screen of improved pool<br>Validation of both pools
Mouse PD-L1 (human Fc-Tag) Produced in HEK293 cells	ACROBiosystems	PD1-M5251	Screens of both pools<br>Validation of both pools
Human CD276 (2Ig) (human Fc-Tag) Produced in HEK293 cells	ACROBiosystems<br>Antibodies Online	B73-H5253<br>ABIN7273959	Screen of pilot pool<br>Screen of improved pool<br>Validation of both pools
Human CD276 (4Ig) (human Fc-Tag) Produced in HEK293 cells	ACROBiosystems	B7B-H5258	Validation of both pools
Mouse CD276 (llama Fc-Tag Produced in HEK293 cells)	Nanobody Core Facility Bonn	–	Screen of pilot pool
Mouse CD276 (human Fc-Tag) Produced in HEK293 cells	ACROBiosystems	B73-M5255	Validation of both pools
Human VTCN1 (llama Fc-Tag) Produced in HEK293 cells	Nanobody Core Facility Bonn	–	Screen of pilot pool
Human VTCN1 (human Fc-Tag) Produced in HEK293 cells	ACROBiosystems	B74-H5256	Screen and validation of improved pool

Third, proper folding, surface presentation, and functionality of the minibinders in a mammalian cellular environment were already assessed during the screening stage through our pooled

mammalian cell surface display system. In this setting, minibinders must be expressed in mammalian cells, traffic through the secretory pathway to the plasma membrane, and bind their cognate target in order to be enriched. Conversely, designs with impaired folding, inefficient surface presentation, or reduced target binding in mammalian cells are expected to be depleted during selection. We consider this a key advantage of our screening strategy over alternative display technologies, such as phage display or yeast display, which do not directly evaluate binder performance in a mammalian cellular context. Of note, the original CD276 HM9 binder was identified through iterative yeast cell surface display and showed poor surface expression in mammalian cells in our hands.

References

1. Nick Pace, C., Scholtz, J. M. & Grimsley, G. R. Forces stabilizing proteins. *FEBS Lett.* **588**, 2177–2184 (2014).
2. Ravichandran, A. *et al.* Structure-guided engineering of protein stability through core hydrophobicity. *Protein Sci.* **34**, e70360 (2025).
3. Listov, D., Goverde, C. A., Correia, B. E. & Fleishman, S. J. Opportunities and challenges in design and optimization of protein function. *Nat. Rev. Mol. Cell Biol.* **25**, 639–653 (2024).
4. Leng, X. *et al. De novo* designed 3-helix bundle peptides and proteins with controlled topology and stability. *Chem. Sci.* **16**, 18632–18641 (2025).
5. Xia, Z. *et al.* Targeting overexpressed antigens in glioblastoma via CAR T cells with computationally designed high-affinity protein binders. *Nat. Biomed. Eng.* https://doi.org/10.1038/s41551-024-01258-8 (2024) doi:10.1038/s41551-024-01258-8.
6. Watson, J. L. *et al.* De novo design of protein structure and function with RFdiffusion. *Nature* **620**, 1089–1100 (2023).
