# Supplementary material for: AI-enabled discovery and biochemical optimization of minibinders targeting cancer cell-surface proteins
Source: Nat Commun. 2026 Aug 20;17:8736. doi: 10.1038/s41467-026-76760-5 (PMC13494018; doi:10.1038/s41467-026-76760-5)
Supplement: Supplementary file 2 [file 41467_2026_76760_MOESM1_ESM.pdf]

Supplementary Figures to the manuscript

AI-enabled discovery and biochemical optimization of minibinders targeting cancer cell-surface proteins

Bianca Broske*, Benjamin A. McEnroe*, Sophie C. Frechen*, Tim N. Kempchen*, Caroline I. Fandrey*, …, Stephan Menzel#, Tobias Bald#, Jonathan L. Schmid-Burgk#, Gregor Hagelueken#,&, Michael Hölzel#,&

*these authors contributed equally
#these authors jointly supervised this work
&Correspondence: michael.hoelzel@ukbonn.de, hagelueken@uni-bonn.de

Supplemental Figure 1

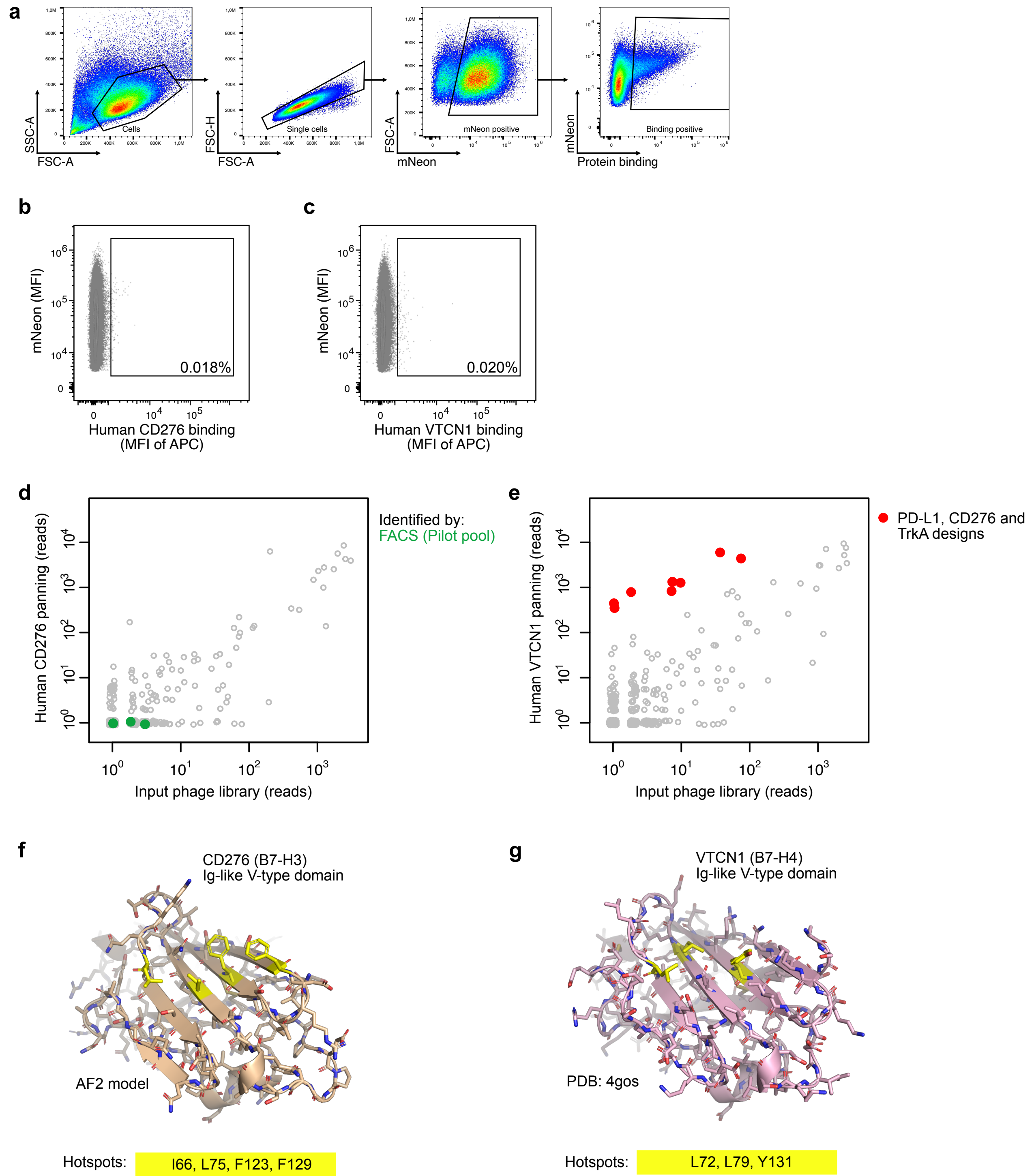

Supplementary Figure 1. CD276 and VTCN1 phage display and hotspot selection for improved pool design.
a Representative FACS plots showing gating strategy for sorting minibinders using the mammalian cell surface display as presented in Fig. 2f and Supplementary Fig. 1b–c, 10d. **b–c** Representative scatter plots illustrating subpopulations of transduced HEK293T cells expressing binders of the pilot pool binding to human CD276 or VTCN1 Fc-fusion protein (produced in HEK293 cells). **d** Scatter plot summarizing the NGS results from phage display and panning with human CD276 protein. **e** As described in c but for panning with human VTCN1 protein (produced in HEK293 cells). Modest enriched candidates were identified as off-target binders. **f** AF2 model of human CD276 structure with side chains. Selected hotspot residues for RFdiffusion design pipeline highlighted in yellow as used for design of the improved pool. **g** As in e, but crystal structure of VTCN1 (PDB: 4gos).

Supplementary Figure 2

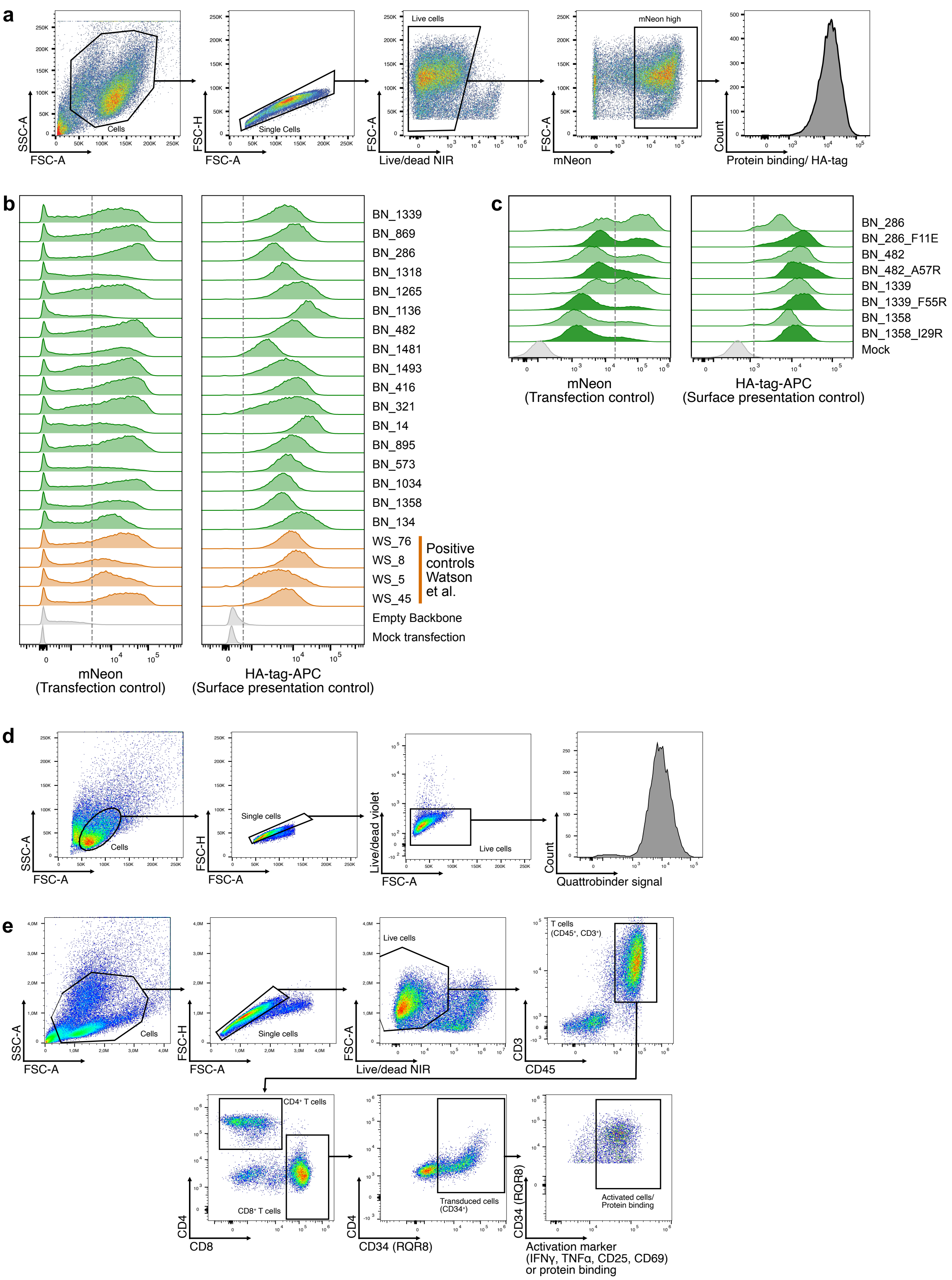

Supplementary Figure 2. Gating strategies for flow cytometry experiments

a Representative FACS plots showing gating strategy for single binder validation using the mammalian cell surface display as presented in Fig. 3b–f, Supplementary Fig. 3a–d, 9d. **b** Flow cytometric analyses of experiments as described in b showing proportions of HEK293T cells expressing PD-L1 AI-minibinders by mNeon positivity and cell-surface expression of AI-minibinders by HA-tag signal. **c** As described in c but for PD-L1 AI-minibinder interface mutants. **d** Representative FACS plots showing gating strategy from flow cytometric stainings with quattrobinders as presented in Fig. 5b–i, Supplementary Fig. 8a–e. **e** Representative FACS plots showing gating strategy used in CAR-T cell co-culture experiments as presented in Fig. 6b–f, 8e–f, Supplementary Fig. 8b–f, 9b–c, 10e.

Supplementary Figure 3

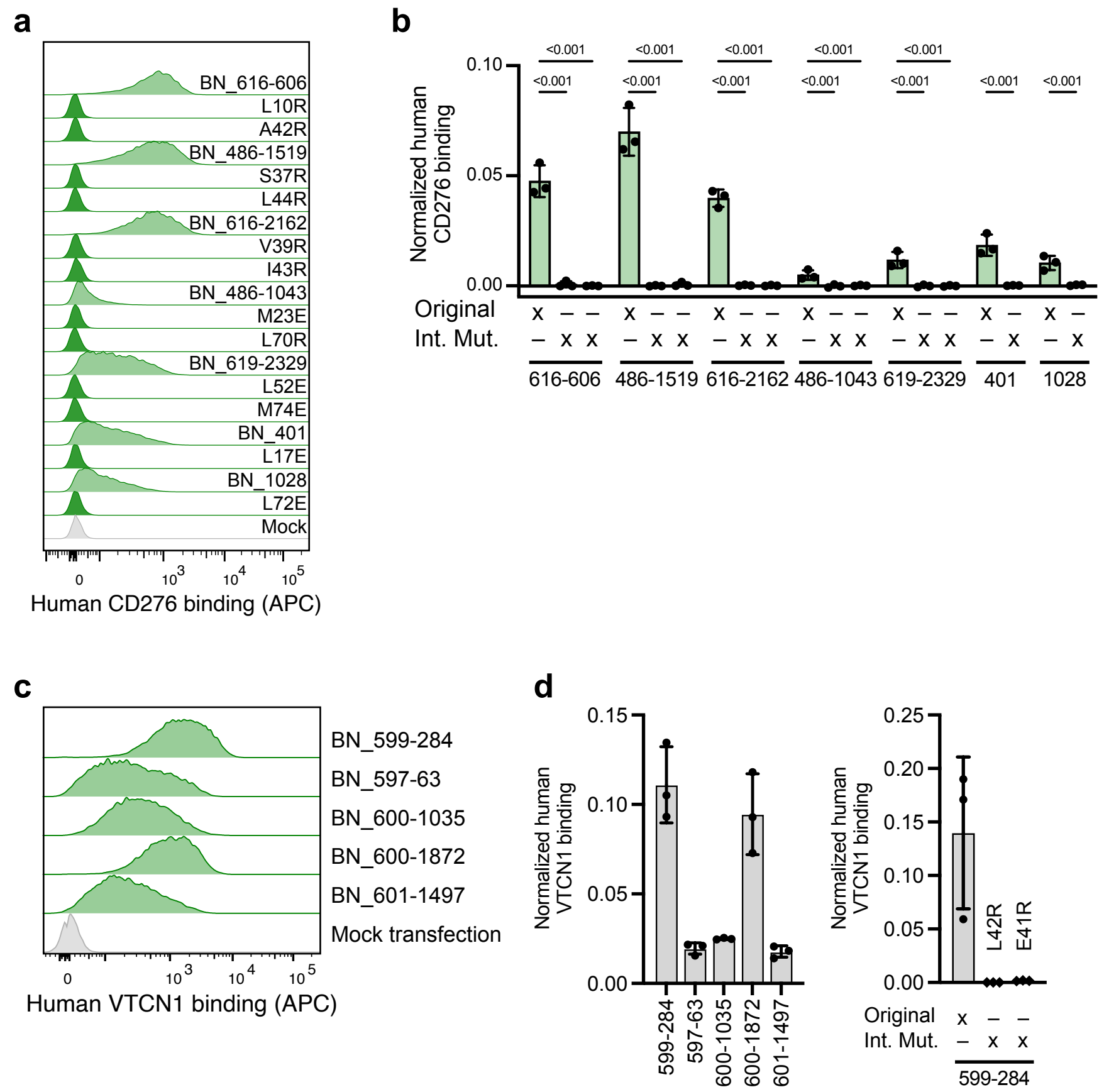

Supplementary Figure 3. CD276 and VTCN1 binder validation.
a Flow cytometric analyses evaluating the binding by HEK293T cell surface display of individual CD276 binders and corresponding interface mutants to human CD276 Fc-fusion proteins (produced in HEK293 cells). **b** Quantification of data shown in a from biological triplicates (n = 3 experiments). Geometric mean fluorescence intensity (MFI) of binding is normalized to HA-tag signal (cell-surface expression of AI-minibinders). **c** Flow cytometric analyses evaluating the binding of individual VTCN1 binders to human VTCN1 Fc-fusion proteins (produced in HEK293 cells) by HEK293T cell-surface display. **d** Quantification of data shown in c from biological triplicates (n = 3 experiments) including the analyses of VTCN1 interface mutants. MFI of binding is normalized to HA-tag signal. Exact p-values are given, non-significant not shown; two-way ANOVA with Šídák's multiple comparisons test. Error bars indicate mean SD.

Supplemental Figure 4

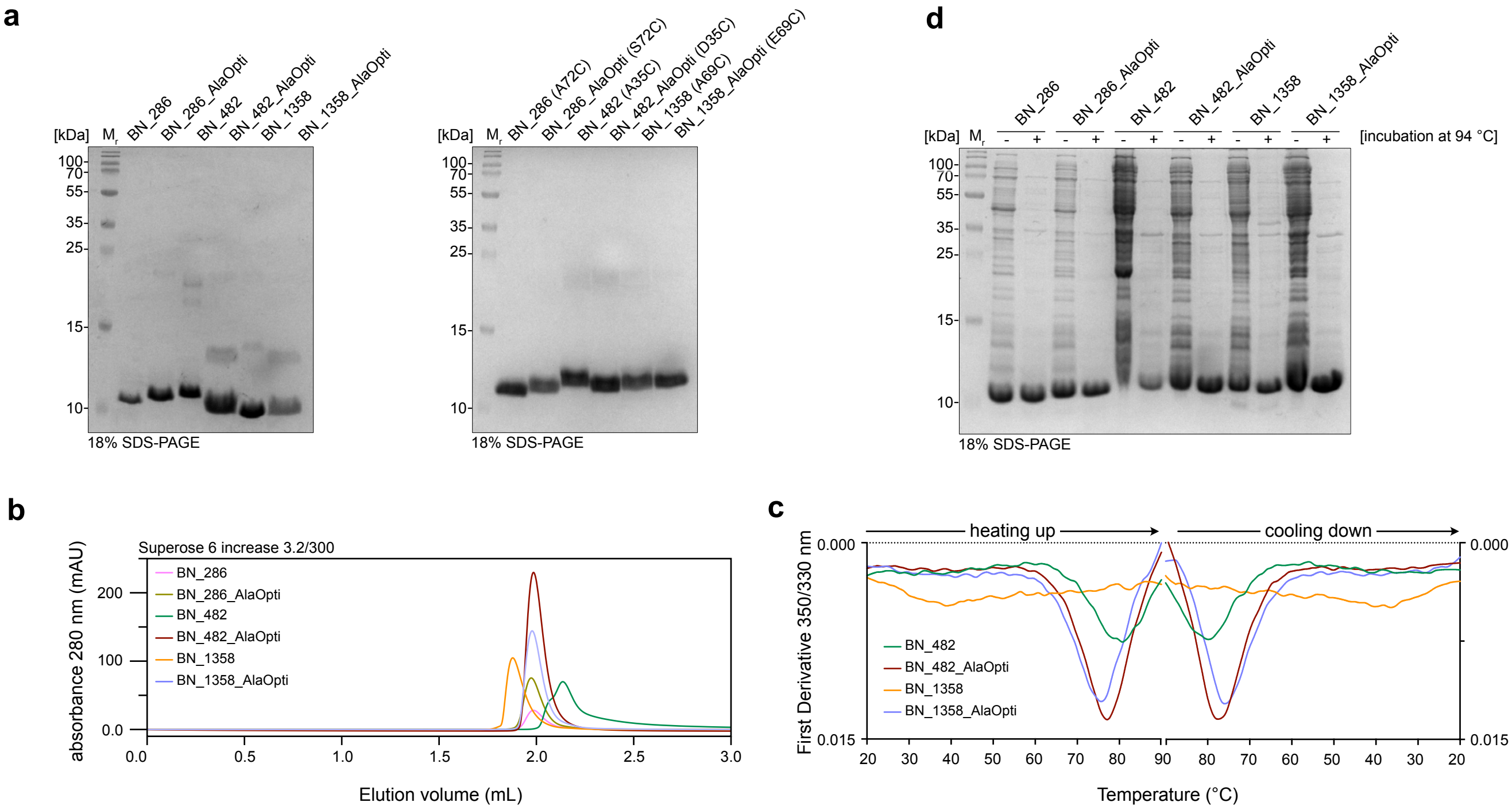

Supplementary Figure 4. Biochemical analysis of AI-minibinder expression and analysis of the thermal stability.
a Coomassie-stained SDS-PAGE of PD-L1 specific AI-minibinders (left panel) or PD-L1 specific AI-minibinders containing single-cysteine mutations (right panel) purified from E. coli. **b** Superpositions of analytical gel filtration runs of selected PD-L1 specific AI-minibinders using a Superose 6 3.2/300 column. **c** Nano differential scanning fluorometry (nanoDSF) experiments of selected PD-L1 specific AI-minibinders. The y-axis shows the first derivative of the corresponding melting curve. The two panels show the unfolding (left) and refolding (right) stages of the experiment. **d** Coomassie-stained SDS-PAGE of E. coli lysates after the overexpression of AI-minibinders with (+) and without (-) incubation at 94 °C for 20 min. The boiled samples were centrifuged and the cleared supernatant was loaded onto the gel.

Supplementary Figure 5

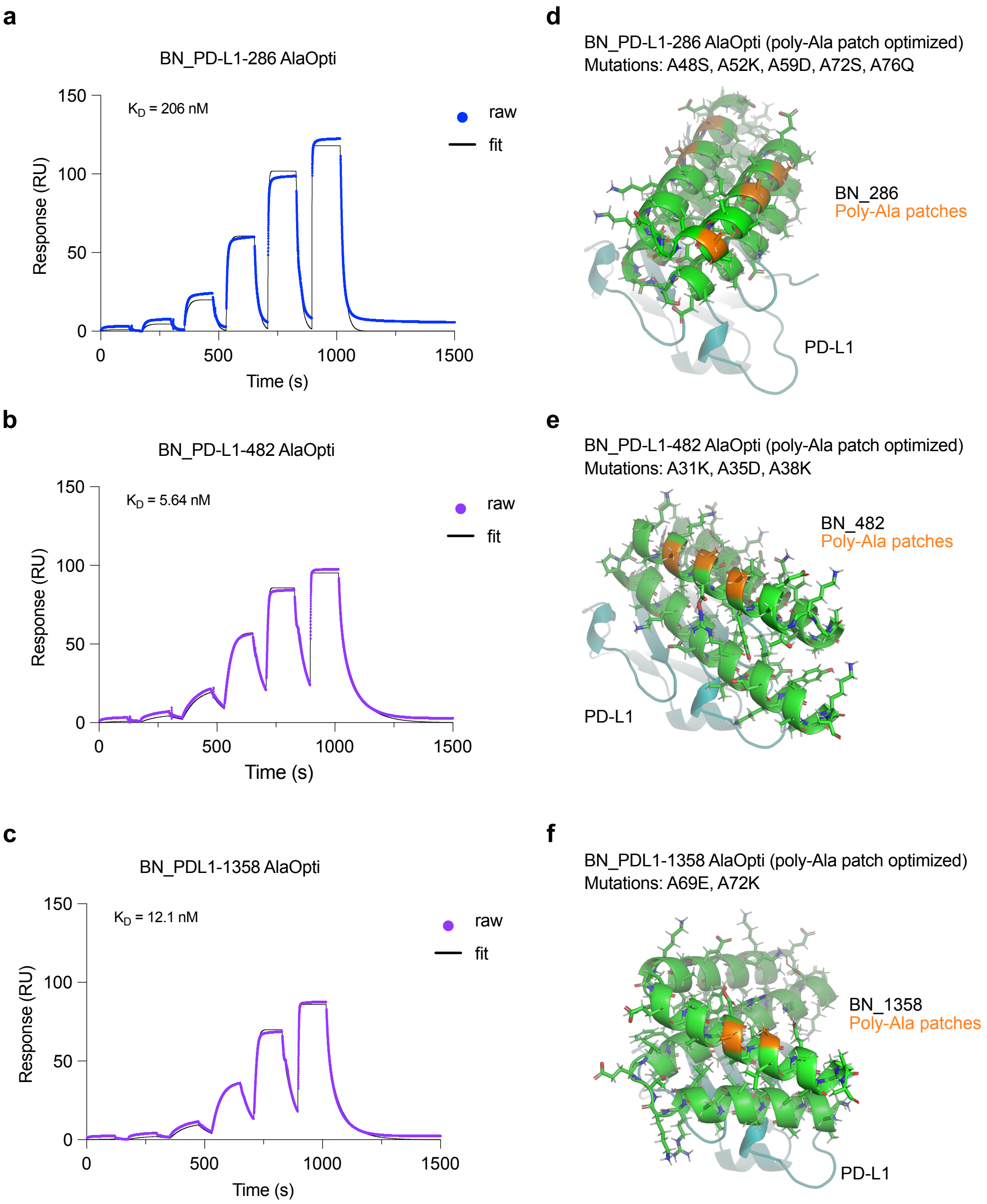

Supplementary Figure 5. Binding affinity measurements of PD-L1 AI-minibinders with optimization of poly-alanine patches.
a-c SPR (surface plasmon resonance) sensorgrams showing raw binding data (colored dots) and fitted curves (black) used to determine binding affinities (K_D) of poly-Ala patch optimized (AlaOpti) PD-L1 AI-minibinders BN_286, BN_482, and BN_1358 purified from E. coli. Five-fold concentration series were used ranging from 6 nM to 5,000 nM (BN_286 AlaOpti), 0.064 nM to 200 nM (BN_482 AlaOpti and BN_1358 AlaOpti). **d-f** Cartoon representation with side chains as predicted by AF2 model in RFdiffusion pipeline showing poly-Ala patches of BN_286, BN_482, and BN_1358 and the introduced mutations to generate AlaOpti variant.

Supplemental Figure 6

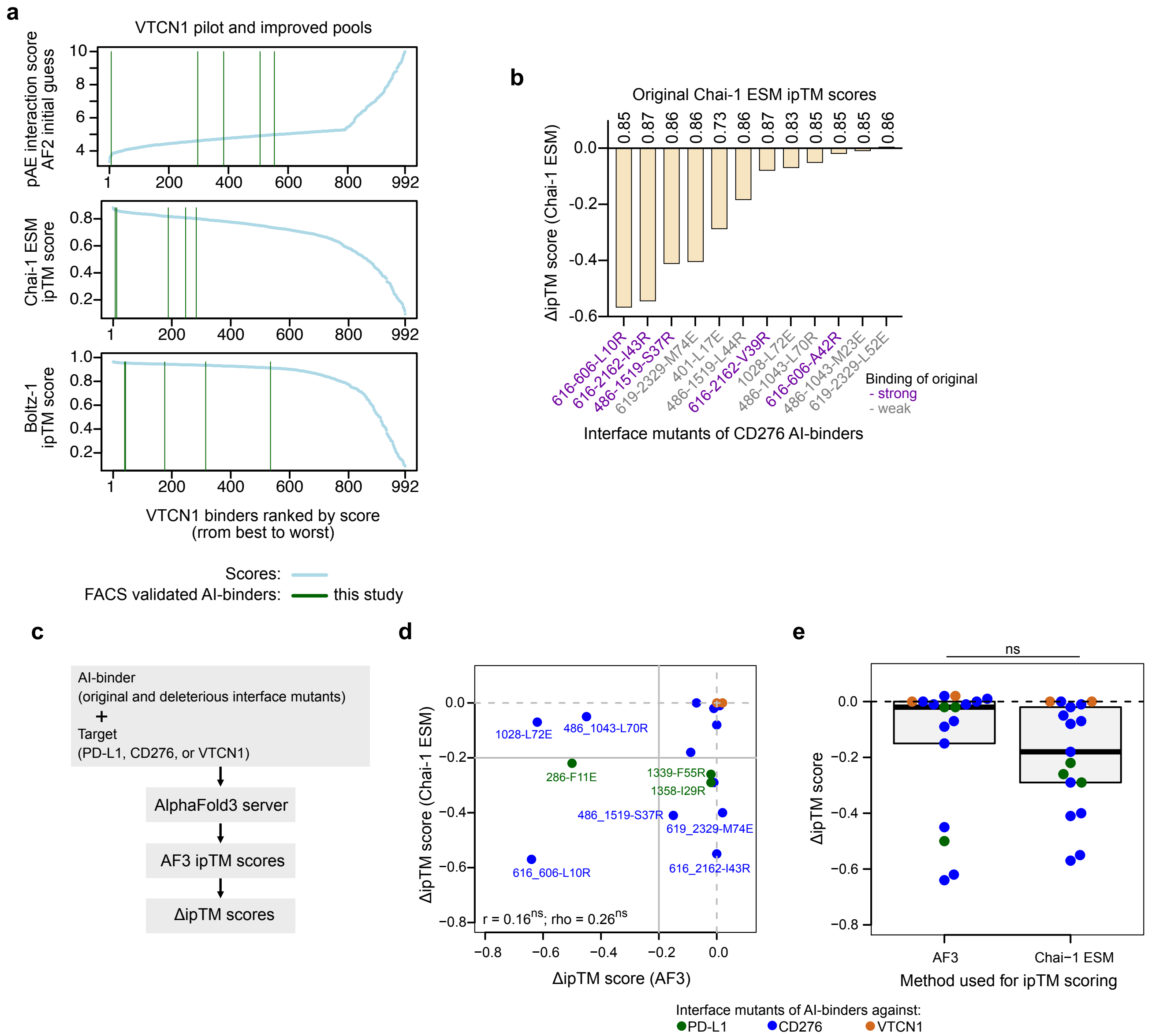

Supplementary Figure 6. Computational rescoring of VTCN1 minibinders, CD276 minibinder interface mutants and comparison to AF3.
a Enrichment plots visualizing correlation between depicted interaction scores and experimental success. AI-minibinders from indicated pools ranked from left to right by interaction scores (best to worst). Colored vertical lines indicate functional AI-minibinders from our study (green). **b** Change in Chai-1 ESM ipTM scores caused by interface mutations in CD276 AI-minibinders. **c** Outline of strategy to determine AlphaFold3 (AF3) ipTM scores and corresponding ΔipTM scores. **d** Scatter plot showing relationship between AF3 and Chai-1 ESM ΔipTM scores. r, Pearson's correlation value; rho, Spearman's rank correlation value; ns, non-significant. **e** Boxplot comparing AF3 and Chai-1 ESM ΔipTM scores. Two-sided Wilcoxon-test; ns, non-significant.

Supplemental Figure 7

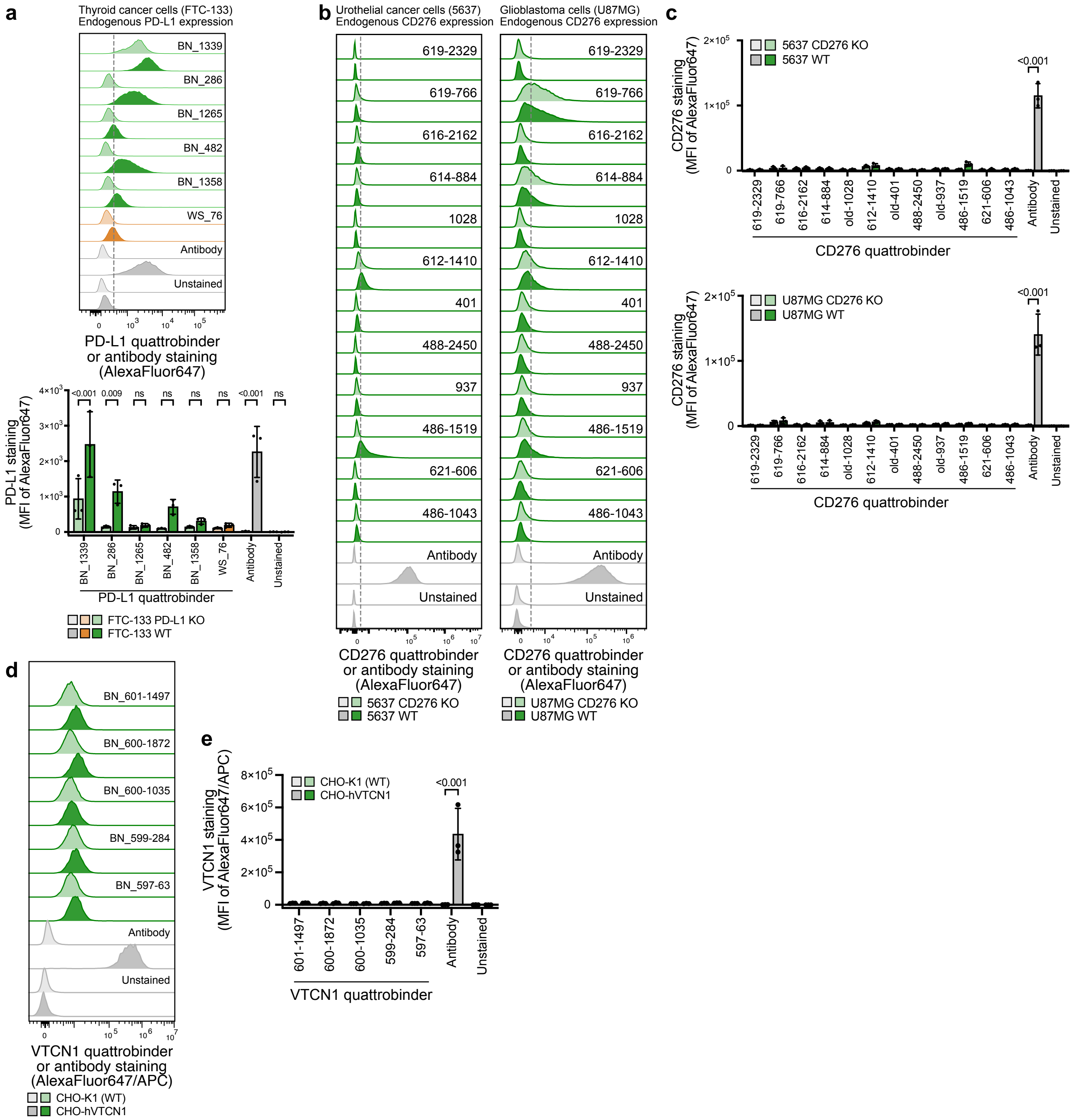

Supplementary Figure 7. Evaluation of CD276 and VTCN1 quattrobinders.
a Representative FACS histograms showing staining with AF647-conjugated PD-L1 quattrobinders (assembled from biotinylated minibinders purified from E.coli) compared to an AF647-conjugated anti-PD-L1 antibody of thyroid cancer FTC-133 wild-type (WT) cells and CRISPR-Cas9-engineered PD-L1 knockout (KO) cells. Quantification of flow cytometry data by geometric mean fluorescence intensity (MFI), performed in biological triplicates (n = 3 experiments). **b** Representative flow cytometry histograms showing staining with AF647-conjugated CD276 quattrobinders (assembled from biotinylated minibinders purified from E.coli) compared to an AF647-conjugated anti-CD276 antibody of CD276 wild-type (WT) and CRISPR-Cas9-engineered CD276 knockout (KO) cancer cells (human 5637 urothelial cancer cells; human U87MG glioblastoma cells). **c** Quantification of experiments described in b by MFI, performed in biological triplicates (n = 3 experiments). **d** Representative flow cytometry histograms showing staining with AF647-conjugated VTCN1 quattrobinders (assembled from biotinylated minibinders purified from E.coli) compared to an APC-conjugated anti-VTCN1 antibody of human VTCN1 overexpressed on Chinese hamster ovarian (CHO) cells (CHO-hVTCN1) and wild-type CHO cells (CHO-K1 (WT)). **e** Quantification of experiment described in d, performed in biological triplicates (n = 3 experiments). Exact p-values are given, non-significant not shown; two-way ANOVA with Šídák's multiple comparisons test. Error bars indicate mean SD.

Supplementary Figure 8

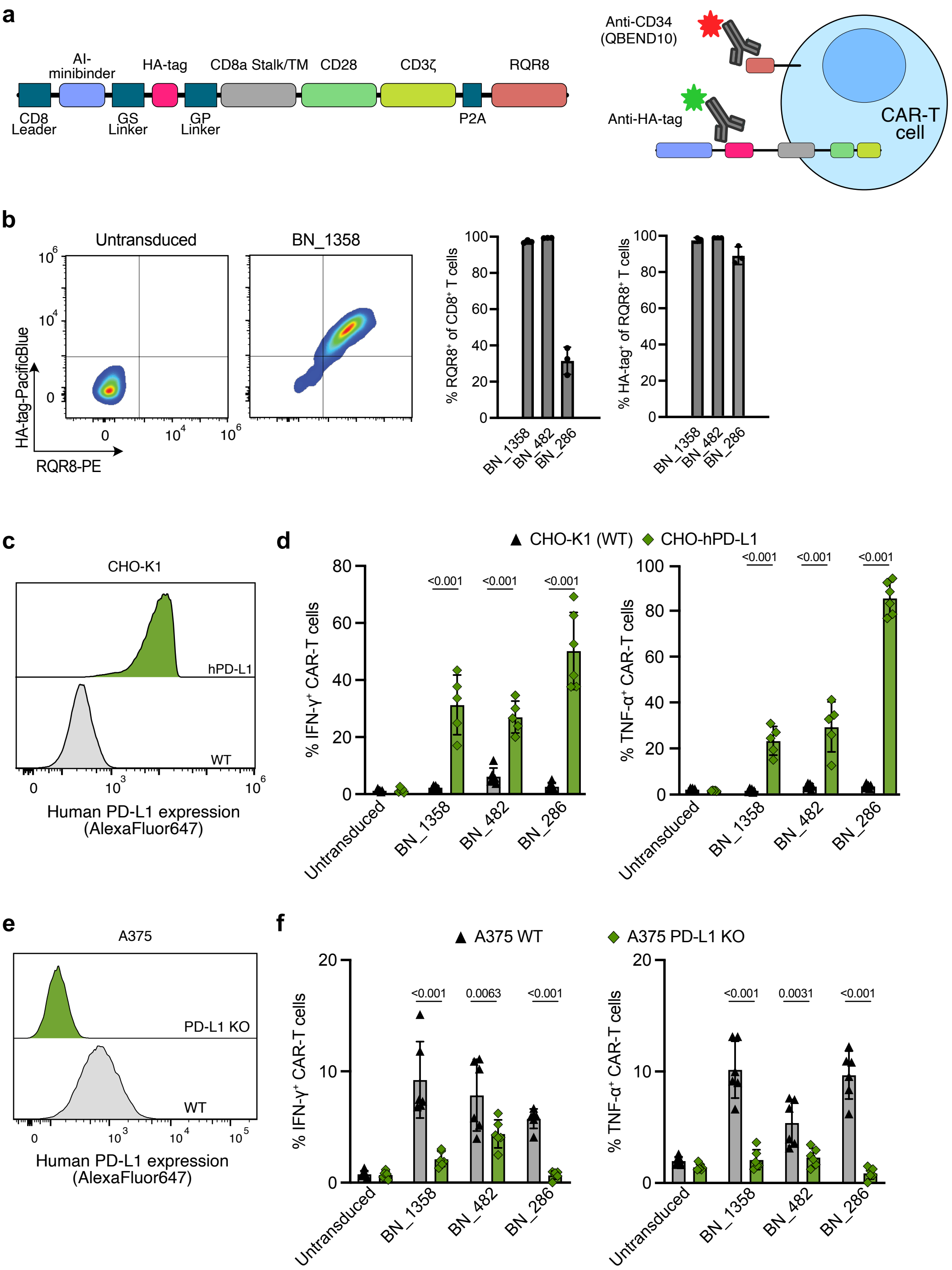

Supplementary Figure 8. PD-L1 AI-minibinder CAR-T cells specifically recognize endogenous PD-L1 expressed on cancer cells. a Outline of deployed second-generation CAR construct and flow cytometric strategy to assess transduction efficiency (RQR8 via anti-CD34 antibody) and CAR cell surface expression (HA-tag) on murine T cells. Cartoon generated using Affinity Publisher 2. **b** Representative flow cytometric plots showing successful transduction and cell surface expression of PD-L1 AI-minibinder CAR (BN_1358) on murine T cells and corresponding quantification (n = 3) with three PD-L1 AI-minibinders. **c** Representative flow cytometry histograms showing overexpression of human PD-L1 on CHO-K1 cells transduced with a construct encoding for human PD-L1 (hPD-L1) versus untransduced control CHO-K1 cells (WT). **d** Activation of PD-L1 AI-minibinder murine CAR-T cells exposed to CHO-hPD-L1 cells or CHO-K1 control cells (1:1 E:T ratio) determined by flow cytometric analyses of intracellular cytokine (IFN-γ, TNF-α) expression (n = 3). **e** Representative flow cytometry histograms showing PD-L1 surface expression on human A375 melanoma wild-type (WT) cells and CRISPR-Cas9 engineered A375 PD-L1 knockout (KO) cells. **f** Activation of PD-L1 AI-minibinder murine CAR-T cells exposed to A375 WT cells and A375 PD-L1 KO cells (1:1 E:T ratio) determined by flow cytometric analyses of intracellular cytokine (IFN-γ, TNF-α) expression (n = 3). Exact p-values are given, non-significant not shown; two-way ANOVA with Šídák's multiple comparisons test. Error bars indicate mean SD.

Supplementary Figure 9

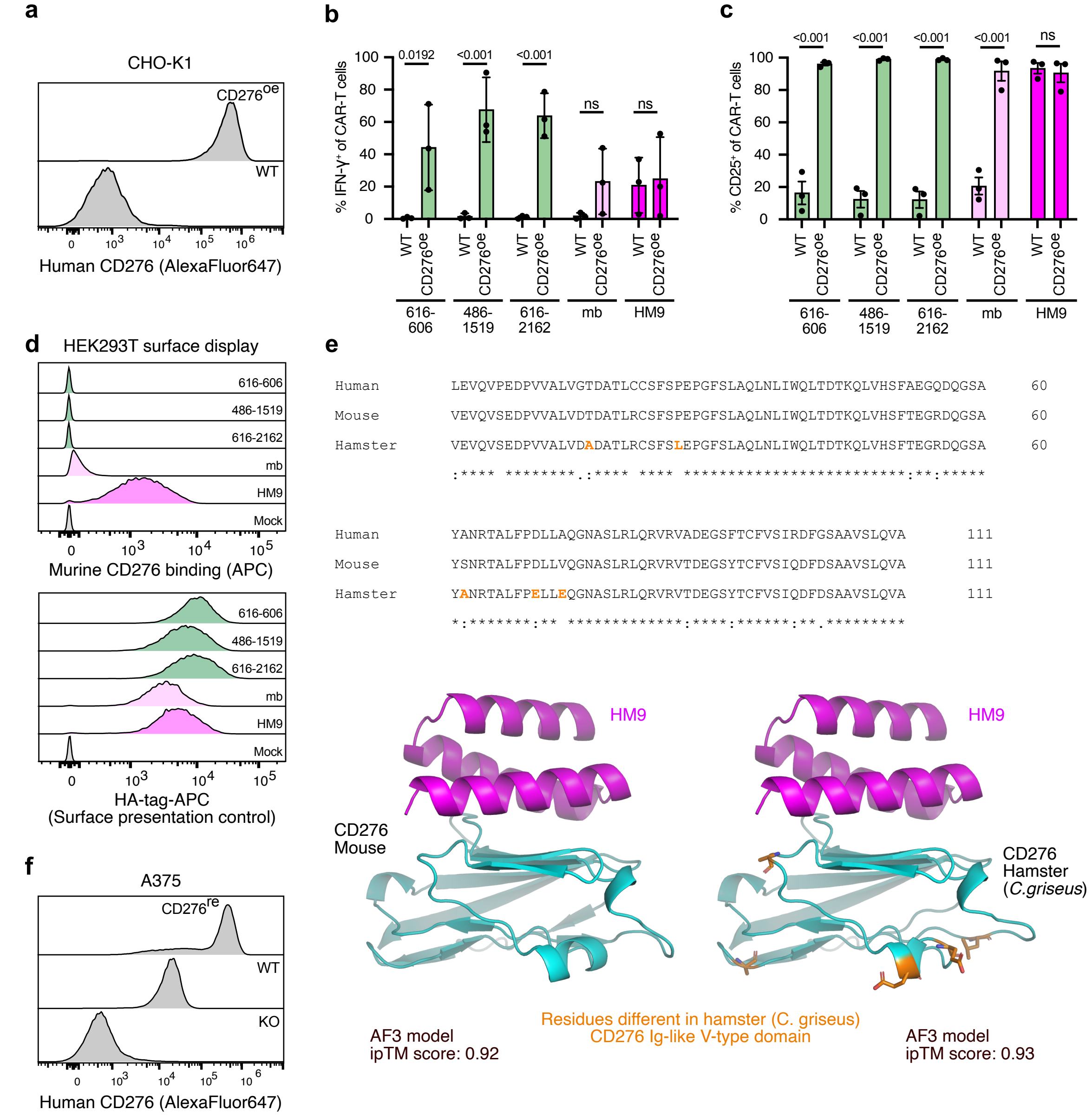

Supplementary Figure 9. CD276 AI-minibinder CAR-T cells recognize overexpressed CD276 on CHO cells and show different profiles of species cross-reactivity.
a Representative flow cytometry histograms showing overexpression of human CD276 on CHO-K1 cells transduced with a construct encoding for human CD276 (CD276^{oe}) versus untransduced control CHO-K1 cells (WT). **b** Activation of CD276 AI-minibinder human CAR-T cells exposed to CHO cells expressing human CD276 (CD276^{oe}) or control cells (1:1 E:T ratio) determined by flow cytometric analyses of intracellular cytokine (IFN-γ) expression. Binders in green were from this study, binders in pink (HM9, mb) were from Xia et al.[30]. Quantification of experiments performed in biological triplicates (n = 3 PBMC donors). **c** Same as described in b but for induced CD25 surface expression on activated human CAR-T cells (n = 3 PBMC donors). **d** Representative flow cytometry histograms showing surface presentation of CD276 AI-minibinders (HA-tag signal) on transfected HEK293T cells and binding to murine CD276. **e** Protein sequence alignment of the Ig-like V-type domain of CD276 from human, mouse, and hamster (C. griseus). Below, cartoon representations of AF3 models showing the HM9 CD276 AI-minibinder in complex with murine (left) and hamster (right) CD276. Residues differing between murine and hamster CD276 are highlighted in orange. AF3 ipTM scores are indicated. **f** Flow cytometry histograms showing endogenous CD276 surface expression on human A375 melanoma cells (WT), CRISPR-Cas9 engineered A375 CD276 knockout (KO) cells, and human CD276 overexpressed on A375 CD276 KO cells. Exact p-values are given; two-way ANOVA with Šídák's multiple comparisons test. Error bars indicate mean SD.

Supplementary Figure 10

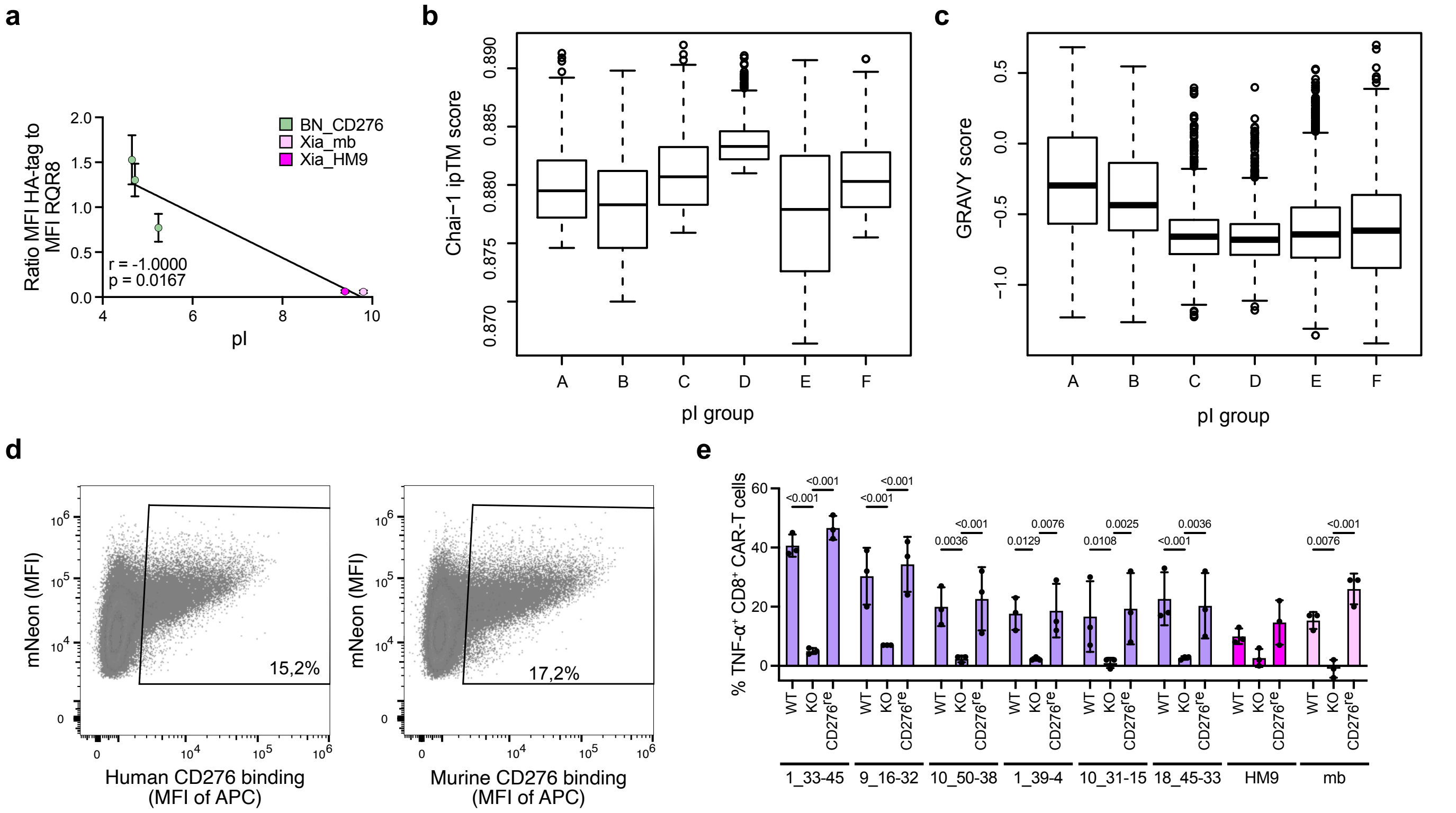

Supplementary Figure 10. Library design of HM9-derived CD276 AI-minibinders and their application as CAR-T cells.

a AI-minibinders expressed as CARs on transduced human T cells were assessed for expression (RQR8) and surface presentation (HA-tag) by flow cytometric analysis of the respective geometric mean fluorescence intensity (MFI). The ratio of surface presentation to expression is plotted against the isoelectric point (pI). Binders in green (BN_CD276) originate from this study, binders in pink (HM9, mb) originate from Xia et al.[30]. **b, c** Chai-1 ipTM scores and GRAVY scores (< 0 indicates hydrophobicity) by isoelectric point (pI) groups from the HM9 variant CD276 minibinder pool. **d** Representative scatter plots illustrating subpopulations of transduced HEK293T cells expressing binders of the HM9 variant CD276 minibinder pool binding to human or murine CD276 Fc-fusion proteins (purified from HEK293 cells). **e** Activation of CD276 AI-minibinder human CAR-T cells exposed to variants of A375 cells determined by flow cytometric analyses of intracellular cytokine (TNF-α) expression (n = 3 PBMC donors). A375 melanoma cells endogenously expressing CD276 (WT), CRISPR-Cas9-engineered CD276 knockout (KO), or reconstituted CD276 KO (CD276^{re}). Exact p-values are given, non-significant not shown; two-way ANOVA with Šídák's multiple comparisons test. Error bars indicate mean SD.
