# Supplementary material for: AI-enabled discovery and biochemical optimization of minibinders targeting cancer cell-surface proteins
Source: Nat Commun. 2026 Aug 20;17:8736. doi: 10.1038/s41467-026-76760-5 (PMC13494018; doi:10.1038/s41467-026-76760-5)
Supplement: Supplementary file 3 [file 41467_2026_76760_MOESM11_ESM.docx]

**Supplementary Table Y. List of Fc fusion proteins either commercially available or provided by core facility.**

| **Protein** | **Vendor** | **Catalogue number** | **Used for** |
| --- | --- | --- | --- |
| Human PD-L1 (llama Fc-Tag) and human PD-L1 ECD | Nanobody Core Facility Bonn | – | Screen of pilot pool and validation (SPR) |
| Human PD-L1 (human Fc-Tag) | ACROBiosystems | PD1-H5258 | Screen of pilot pool  Screen of improved pool  Validation of both pools |
| Mouse PD-L1 (human Fc-Tag) | ACROBiosystems | PD1-M5251 | Screens of both pools  Validation of both pools |
| Human CD276 (2Ig) (human Fc-Tag) | ACROBiosystems  Antibodies Online | B73-H5253  ABIN7273959 | Screen of pilot pool  Screen of improved pool  Validation of both pools |
| Human CD276 (4Ig) (human Fc-Tag) | ACROBiosystems | B7B-H5258 | Validation of both pools |
| Mouse CD276 (llama Fc-Tag) | Nanobody Core Facility Bonn | – | Screen of pilot pool |
| Mouse CD276 (human Fc-Tag) | ACROBiosystems | B73-M5255 | Validation of both pools |
| Human VTCN1 (llama Fc-Tag) | Nanobody Core Facility Bonn | – | Screen of pilot pool |
| Human VTCN1 (human Fc-Tag) | ACROBiosystems | B74-H5256 | Screen and validation of improved pool |
