# Supplementary material for: AI-enabled discovery and biochemical optimization of minibinders targeting cancer cell-surface proteins
Source: Nat Commun. 2026 Aug 20;17:8736. doi: 10.1038/s41467-026-76760-5 (PMC13494018; doi:10.1038/s41467-026-76760-5)
Supplement: Supplementary file 4 [file 41467_2026_76760_MOESM2_ESM.docx]

**Description of additional supplementary data**

**Supplementary Data 1: Pilot pool target proteins and hotspot definition**

The table lists the proteins and corresponding hotspot residues used as target protein input for the design runs generating the minibinder ‘pilot pool’.

**Supplementary Data 2: Minibinder designs in the pilot pool**

The table lists all binder sequences within the minibinder ‘pilot pool’ with their targeted proteins (with remark if minibinders were generated by the authors (Bonn) or the Baker lab (Baker)).

**Supplementary Data 3: Design statistics of minibinders in the pilot pool**

The table lists all binders within the minibinder ‘pilot pool’ with their prediction scores.

**Supplementary Data 4: Improved pool target proteins and hotspot definition**

The table lists the proteins and corresponding hotspot residues used as target protein input for the design runs generating the minibinder ‘improved pool’.

**Supplementary Data 5: Minibinder designs in the improved pool**

The table lists all binder sequences within the minibinder ‘improved pool’ with their targeted proteins.

**Supplementary Data 6: pAE interactions scores of minibinders in the improved pool**

The table lists all binders within the minibinder ‘improved pool’ with their pAE interaction scores.

**Supplementary Data 7: Change of ipTM for interface mutants**

The table lists minibinders with their original sequences (WT) and matching interface mutants. AlphaFold3 (AF3) and Chai-1 generated ipTM scores are listed with the calculated differences of the interface mutants to their corresponding WT minibinders.

**Supplementary Data 8: Primer and cDNA sequences**

The table lists primer sequences used for oligo library amplification and barcoding PCRs for NGS. cDNA sequences were used to generate overexpression and re-expressing cell lines.

**Supplementary Data 9: Fc fusion proteins**

The table lists the proteins that were used for the minibinder screens and validation experiments.

**Supplementary Data 10: Detailed parameters from SPR data**

The table lists additional details from SPR experiments including k_D_ [M], R_max_ [RU], affinity Chi^2^ [RU^2^], and the standard errors for steady state affinity and 1:1 binding kinetic measurements.

**Supplementary Data 11: Minibinder designs in the HM9 pool**

The table lists all binder sequences within the minibinder ‘HM9 pool’ with their ipTM scores and selected biochemical properties.

**Supplementary Data 12: Glycosylation sites in validated minibinders**

The table lists all minibinders that were validated individually with asparagine marked in red and glycosylation motifs marked in blue.
